# Maturation of Induced Pluripotent Stem Cell Derived Hepatocytes by 3D-Culture

**DOI:** 10.1371/journal.pone.0086372

**Published:** 2014-01-22

**Authors:** Richard L. Gieseck III, Nicholas R. F. Hannan, Roque Bort, Neil A. Hanley, Rosemary A. L. Drake, Grant W. W. Cameron, Thomas A. Wynn, Ludovic Vallier

**Affiliations:** 1 Wellcome Trust–Medical Research Council Stem Cell Institute, Anne McLaren Laboratory for Regenerative Medicine, Department of Surgery, University of Cambridge, Cambridge, United Kingdom; 2 Immunopathogenesis Section, Laboratory of Parasitic Diseases, National Institute of Allergy and Infectious Diseases, National Institutes of Health, Bethesda, Maryland, United States of America; 3 Unidad de Hepatología Experimental, Centro de Investigación Biomédica en Red de Enfermedades Hepáticas y Digestivas, Instituto de Investigación Sanitaria La Fe, Valencia, Spain; 4 Centre for Endocrinology and Diabetes, Institute of Human Development, Faculty of Medical & Human Sciences, Manchester Academic Health Sciences Centre, University of Manchester, Manchester, United Kingdom; 5 TAP Biosystems, Royston, United Kingdom; 6 Wellcome Trust Sanger Institute, Hinxton, United Kingdom; University of Newcastle upon Tyne, United Kingdom

## Abstract

Induced pluripotent stem cell derived hepatocytes (IPSC-Heps) have the potential to reduce the demand for a dwindling number of primary cells used in applications ranging from therapeutic cell infusions to *in vitro* toxicology studies. However, current differentiation protocols and culture methods produce cells with reduced functionality and fetal-like properties compared to adult hepatocytes. We report a culture method for the maturation of IPSC-Heps using 3-Dimensional (3D) collagen matrices compatible with high throughput screening. This culture method significantly increases functional maturation of IPSC-Heps towards an adult phenotype when compared to conventional 2D systems. Additionally, this approach spontaneously results in the presence of polarized structures necessary for drug metabolism and improves functional longevity to over 75 days. Overall, this research reveals a method to shift the phenotype of existing IPSC-Heps towards primary adult hepatocytes allowing such cells to be a more relevant replacement for the current primary standard.

## Introduction

With uses in drug screening, toxicology studies, cell-based therapies, and *in vitro* disease modeling, primary human hepatocytes (PHHs) are in high demand. However, lack of sufficient organ donors, poor longevity *in vitro*, and difficulties with dedifferentiation [1], [2] have led researchers to seek alternative sources to bridge the gap between clinical demands and cell availability. Hepatocyte-like cells generated from hIPSCs (IPSC-Heps) have shown great promise to satisfy this need by providing an inexhaustible source of cells that mirror the genotype of the donor[3]–[6]. Nevertheless, current differentiation protocols and culture conditions often produce cells with a fetal identity and suboptimal functionality compared to that of PHHs.

Several studies have demonstrated that culturing isolated PHHs in a 3D format averts many effects of dedifferentiation and can partially reverse this process in cells that have been cultured for short term in monolayer (2-Dimensional conditions) [7]. Such 3D cultures have been shown to return the function of several xenobiotic metabolizing enzymes to *in vivo* levels [8], [9], reestablish cellular polarization and canalicular structure [9], [10], and maintain other liver specific functions such as albumin secretion, glycogen synthesis, and lipid storage [7], [9]. Additionally, it has been established that the presence and maintenance of cell-cell junctions is critical to preservation of the mature hepatic phenotype [11]. However, 3D culture systems currently available are often unwieldy and overly complex, leading to poor reproducibility and restricting use to a few labs with highly specialized equipment. Such methods, often based upon embryoid body differentiation, are not compatible with high throughput screening and remain difficult to apply to IPSC-Heps, which require long term, reproducible culture for functional differentiation and subsequent application in research and industry.

Based on these findings, we hypothesized that the phenotypic profile of IPSC-Heps could be shifted towards PHHs by transferring IPSC-Heps, which were fully differentiated in 2D, into a 3D culture system. Furthermore, we hypothesized that the maintenance of cell-cell junctions during the transfer procedure would be vital to the preservation and maturation of the hepatic phenotype. To test this, we conducted a direct comparison of IPSC-Heps cultured on traditional 2D tissue culture plastic and within the Real Architecture for 3D Tissues (RAFT) system.

This 3D culture matrix is based upon the concept of concentrating a cell-seeded collagen hydrogel by removing interstitial fluid [12], [13] and allows for easily reproducible, type-I collagen based, 3D cultures in a 96-well format. A neutralized collagen solution is mixed with cells and subsequently is heated to induce fibrillogenesis and encapsulate the cells *in situ* (**Figure S1a**). A biocompatible absorber is placed on top of the collagen hydrogel in order to remove fluid and collapse the construct to physiological collagen densities. The low level of variability between wells and plates, and the ability to easily control cell and matrix density to produce physiologically relevant constructs, made the RAFT system an ideal choice over traditional collagen sandwich models. The single component, defined nature of the construct made the system superior to Matrigel and other ECM-cytokine mixtures, which often yield high batch to batch variations and can confound differentiation procedures. Additionally, the 96-well format and the lack of need for complex, specialized equipment was perfect for high throughput analyses.

In order to analyze the effects of this 3D culture system on IPSC-Hep maturation, three IPSC lines were differentiated for 25 days towards the hepatic lineage using a common 2-D differentiation protocol (**Figure S1**) [3]. At this time, cells were split into three sample groups and further differentiated for 10 or 20 days. Sample groups consisted of: 1) 2D control; 2) 3D culture in which the cells were transferred to the RAFT matrix as small epithelial clumps with cell-cell junctions intact (**Figure S1b/c**); 3) 3D culture in which the cells were completely dissociated, disrupting the existing cell-cell junctions before transfer to the RAFT matrix (**Figure S1c**). The three sample groups allowed us to simultaneously probe the effects of 3D culture, maintenance of cell-cell junctions, and culture time on the maturation of IPSC-Heps.

## Materials and Methods

### Ethics Statement

Human iPS cell derivation and culture: Ethics for the iPSC lines used in this study were approved under Addenbrooke’s Hospital reference no. 08/H0311/201; R&D no. A091485. Additional information can be found elsewhere [5]. Adult Hepatocytes: Liver samples were obtained in agreement with the rules of the hospital's (Hospital La Fe, Valencia) ethic’s committee (CEIC, Comite Etico de Investigación Clínica; approval number 2009/00111). Fetal Hepatocytes: Human fetal tissue sample collection was approved by NorthWest Ethics Committee (13/NW/0205). Additional information can be found elsewhere [14]. Written informed consent from the donor or the next of kin was obtained for use of all samples used within this study.

### hIPSC Maintenance

Tissue culture plastic (Corning) coated with porcine gelatin (1 g/L; sigma) dissolved in water for embryo transfer (Sigma) for 30 minutes was pre-conditioned with MEF medium consisting of Advanced DMEM/F-12 (Invitrogen), 10% FBS (Biosera), 1% 200 mM L-glutamine (Invitrogen), 1% penicillin/streptomycin (10,000 U/mL; Invitrogen), and 0.0007% *β*-mercaptoethanol (Sigma) for at least 12 hours prior to plating IPSC colonies. hIPSCs were maintained feeder-free at 37°C, 5% CO_2_, 20% O_2_ in chemically-defined, serum-free IPSC maintenance medium (CDM-PVA) consisting 0.5 g of PVA (Sigma) dissolved in 250 mL of DMEM/F-12, GlutaMAX (Invitrogen), 250 mL of IMDM (Invitrogen), 5 mL of chemically defined lipid concentrate (Invitrogen), 20 µL of thioglycerol ≥97% (Sigma), 350 µL of insulin (10 mg/mL; Roche), 250 µL of transferrin (30 mg/mL; Roche) and 5 mL of penicillin/streptomycin (10,000 U/mL; Invitrogen) supplemented with Activin A (10 ng/mL; R&D) and FGF2 (12 ng/mL; R&D) [3]. Media was changed daily and cells were passaged every 5–7 days using a 1∶1 mixture of collagenase IV (400 mL of Advanced DMEM/F-12, 100 mL of KnockOut Serum Replacement (Invitrogen), 5 mL of 200 mM L-glutamine, 3.5 µL of *β*-mercaptoethanol (14.3 M), and 500 mg of collagenase type IV (Invitrogen)) and dispase II (500 mg of dispase II (Invitrogen) dissolved in 500 ml of Advanced DMEM/F12) [3]. The three lines utilized in this study were BBHX8 [5], Line-B7 [4], [5] (Referred to as BOB7 RM in this study), Line-B5 [4] (Referred to as BOB5 SC in this study).

### Differentiation Protocols

#### 2D common progenitor

IPSC lines were split (day 0) and maintained for 48 hrs in CDM-PVA supplemented with Activin A and FGF2 (media was changed daily for all subsequent steps, and cells were differentiated at 37°C, 5% CO_2_, 5% O_2_, unless stated otherwise). On days 2–3, cells were differentiated in CDM-PVA supplemented with Activin A (100 ng/mL), FGF2 (80 ng/mL), BMP4 (10 ng/mL; R&D), 10 µM LY-294002 (Promega), and 3 µM Stemolecule CHIR99021 (StemGent). On day 4, cells were differentiated in CDM-PVA supplemented with Activin A (100 ng/mL), FGF2 (80 ng/mL), BMP4 (10 ng/mL; R&D), and 10 µM LY-294002. On day 5, cells were differentiated in RPMI Medium (RPMI 1640 Medium, GlutaMAX (Invitrogen), 2% B-27 Serum-Free Supplement (50X) (Invitrogen), 1% MEM Non-Essential Amino Acids Solution (100X) (Invitrogen), 1% penicillin/streptomycin) supplemented with Activin A (100 ng/mL) and FGF2 (80 ng/mL). On day 6, cells were expanded in RPMI medium supplemented with Activin A (50 ng/mL). On day 7, cells were split using Cell Dissociation Buffer (Enzyme-free, Hank's; Invitrogen) and were plated in gelatin-coated, MEF media conditioned 6-well plates at a density of 105,000 cells/cm^2^ in RPMI+Activin A (50 ng/mL)+Y-27632 2HCl (10 µM Selleckchem) [15]. Cells were maintained in RPMI+Activin A (50 ng/mL) on days 8–9. From day 10 onward, cells were matured in Hepatozyme-SFM (Invitrogen) supplemented with 1% 200 mM L-glutamine, 1% penicillin/streptomycin, 2% MEM Non-Essential Amino Acids Solution (100X), 2% chemically defined lipid concentrate, 0.14% insulin, 0.28% transferrin, hepatocyte growth factor (50 ng/mL, Peprotech), and oncostatin M (10 ng/mL, R&D) with media changed every other day.

#### 3D-Single cell culture

Cultures designated for 3D single cell culture followed the 2D common progenitor protocol described above until day 25. At day 25, media was removed, wells were washed with DPBS (Invitrogen), and 1 mL of Cell Dissociation Buffer pre-warmed to 37°C was placed in each well. The plates were incubated at 37°C, 5% CO_2_, 5% O_2_ for 15 minutes (at which time half of the wells were subjected to the clump culture protocol below) or 45 minutes, until cells dispersed as single cells. Cells were pelleted and washed twice with Hepatozyme-SFM. Cells were counted and resuspended in Hepatozyme-SFM at a density of 1.39×10^7^ cells/mL for use in the RAFT system (RAFT Standard Protocol available online; TAP Biosystems). Cells embedded within 3D cultures were maintained in Hepatozyme-SFM+supplements with media changes every other day.

#### 3D-Clump culture

Cultures designated for 3D clump culture followed the 2D common progenitor protocol and 3D single cell protocol above until the 15-minute dissociation step. At this point, cells were removed from the surface in clumps using manual perturbation with a 5 mL serological pipette tip. Cells were pelleted and washed twice with Hepatozyme-SFM. Cell count was estimated using the count from the single cells, and cells were resuspended in Hepatozyme-SFM at a density of 1.39×10^7^ cells/mL for use in the RAFT system (RAFT Standard Protocol available online; TAP Biosystems). Cells embedded within 3D cultures were maintained in Hepatozyme-SFM+supplements with media changes every other day.

### Primary Human Controls

#### Adult hepatocytes

Liver samples were obtained in agreement with the rules of the hospital's ethic’s committee. None of the donors (4 men aged between 54 and 80) were regular consumers of alcohol or of other drugs and were not suspected of harboring any infectious disease. Human hepatocytes were isolated from liver biopsies (<5 g) using a two-step collagenase perfusion technique. Hepatocytes were seeded and cultured as previously described in detail [16].

#### Fetal hepatocytes

RNA isolated from human fetal liver samples relating to an approximate gestational age of 7.5 weeks was generously donated by Drs. Andrew Berry and Neil Hanley of the University of Manchester.

### Immunocytochemistry

Cells were fixed for 30 minutes at 4°C in 4% paraformaldehyde (PFA) and washed 3 times with DPBS. Cells were blocked for 1 hour with DPBS containing 1% donkey serum (Serotec Ltd.), 1% Triton X-100 (Sigma). Cells were incubated for 1 hour at room temperature with the following primary antibodies diluted in the blocking solution: A1AT (1∶100; DAKO, cat. no. A0012), AFP (1∶100; DAKO, cat. no. A0008), ALB (1∶100; DAKO, cat. no. A0008), ASGPR (1∶100; Thermo Scientific, cat. No. MA1-40244), *β*-Catenin (1∶100; Abcam, cat. No. ab32572 ), CD26 (1∶100; Abcam, cat. no. ab28340), CK18 (1∶100; SantaCruz, cat. no. sc6259), HNF4 (1∶100; SantaCruz, cat. no. sc6556), Ki-67 (1∶100; Abcam, cat. no. ab15580), MRP2 (1∶100; Abcam, cat. no. ab3373). Cells were washed three times with PBS for 30 minutes each. Cells were incubated for 1 hour at room temperature with appropriate secondary antibodies diluted in the blocking solution: Alexa Fluor 488 Series (1∶1000, Invitrogen, cat. nos. A-11055/21202/21206) and Alexa Fluor 568 Series (1∶1000, Invitrogen, cat. nos. A-10037/10042/11057). Nuclei were stained using bisbenzimide (1∶10,000 in DPBS; sigma) for 30 minutes. Cells were then washed three times with PBS for 30 minutes each and then imaged using an LSM700 laser scanning confocal microscope (Carl Zeiss).

### Periodic Acid Staining

Fixed samples were triple rinsed with deionized water and then placed in 0.5% periodic acid solution (Thermo Scientific) for 5 minutes at room temperature. Samples were then rinsed with deionized water for 5 minutes before being submerged in Schiff Reagent (Thermo Scientific) for 15 minutes. Samples were rinsed with lukewarm tap water for 10 minutes. Samples were then counterstained with Hematoxylin I (Thermo Scientific) for 1 minute. Samples were rinsed with deionized water for 30 seconds and placed in 12 mM sodium bicarbonate for 1 minute. Samples were rinsed once with deionized water and then twice with 100% ethanol for 1 minute each. Samples were triple rinsed for one minute each with 120 mM hydrochloric acid in 70% ethanol before imaging.

### Oil Red O Staining

Fixed samples were washed with tap water for 5 minutes. Samples were then rinsed with 60% isopropanol for 5 minutes. 30 mL Oil Red O Solution (0.5 g Oil Red O (Sigma) in 100 mL isopropanol) was mixed with 20 mL distilled water and filtered using a 0.24 µm vacuum filter to making a working solution. Samples were submerged in the working solution for 15 minutes and then rinsed with 60% isopropanol. Samples were counterstained with Hematoxylin I (Thermo Scientific) for 1 minute. Samples were rinsed with deionized water for 30 seconds and placed in 12 mM sodium bicarbonate for 1 minute. Samples were triple rinsed with DPBS before imaging.

### Scanning Electron Microscopy

#### Sample preparation

Incisions through areas of interest within PFA fixed 3D cultures were made manually using a scalpel and bright field microscope. Sections of interest were fixed with 1.5% glutaraldehyde (EM-grade; Sigma) in 1 M sodium cadodylate (Sigma) for 2 hours. Sections were rinsed with 50%, 90% and 100% ethanol for 5 min, 5 min, and 15 min respectively. The sample was saturated with hexamethyldisilazane (Sigma) three times for 3 minutes each and then dried overnight in a chemical safety cabinet. Samples were mounted using double-sided carbon tape using minimal force to ensure adhesion. An SC7640 sputter coater (Polaron) was used to coat the samples with Au for 90 seconds.

### Quantitative RT-PCR (qPCR)

Total RNA was extracted using GenElute Mammalian Total RNA Miniprep Kit (Sigma). 500 ng of total RNA were reverse-transcribed using Superscript II Reverse Transcriptase (Invitrogen). qPCR reaction mixtures were prepared as described (SensiMix SYBR Low-ROX Kit protocol; Bioline). The mixture was denatured at 95°C for 10 minutes, cycled 40 times (95°C for 30 seconds, 60°C for 30 seconds, 72°C for 30 seconds), followed by final extension at 72°C for 10 minutes. Primer templates can be found in the Supplementary Information. qPCR reactions were performed using a Stratagene Mx3005P in technical duplicates and biological triplicates. All genes were normalized to the geometric mean of PBDG, RPLP0, GAPDH, and HDAC and were normalized to the expression of undifferentiated IPSCs using the ΔΔCt method unless stated otherwise. HDAC was utilized due to its very stable expression upon liver differentiation. This was validated by several control experiments comparing HDAC expression to other common “housekeeping genes” (data not shown). Primer sequences used in this study can be found in **Table S1**. Statistical significance is shown in **Tables S2–S7**.

### Cytochrome P450 Activity

CYP3A4 activity for the 10 day intervals was measured using P450-Glo Assays (Promega). Cells were incubated with 3 µM luciferin-IPA in Hepatozyme-SFM for 60 minutes prior to media collection. Luminescence was measured using a GloMax 96 Microplate Luminometer (Dual Injectors; Promega) using the built-in P450-Glo acquisition protocol. CYP3A4 Activity for the D35 2D sample was also assessed by the rate of conversion of Midazolam to 1′-HO-Midazolam using HPLC-MS. HPLC-MS analysis was performed only on one sample group (2D day 35, **Figure S10**) since this analysis requires complete sacrifice of the culture. This sample was analysed first using the P450-Glo assay and then using HPLC-MS, serving as a way to link the two methods of measuring CYP3A4. This allowed us to compare the functionality of our time course experiment (completed using the P450-Glo assay) to the 35 adult primary samples, which were analysed using the HPLC-MS analysis through the InnovaLiv project, without having to sacrifice all groups for this analysis.

### Protein Quantification Assays

All protein quantification assays were performed by the Cambridge Biomedical Research Centre Core Biochemical Assay Laboratory following the protocols listed below.

#### Alpha-1-antitrypsin DELFIA

A1AT was measured using a time-resolved fluorescence immunoassay on the DELFIA assay platform. Nunc MaxiSorp plates were coated with rabbit anti-human A1AT polyclonal antibody (Siemens) diluted in bicarbonate coating buffer. The plate was incubated overnight and washed four times with DELFIA wash buffer (PerkinElmer) before blocking with 300 µL of 1% BSA in PBS for 1 hour. The plate was washed four more times with DELFIA wash buffer before use. The assay was calibrated with a human serum standard (Siemens). The standard was serial diluted in DELFIA multibuffer (PerkinElmer) to produce 9 standards with a concentration range of 500 to 3.9 ng/mL. Multibuffer was used as the zero concentration standard. 90 µL of multibuffer was added to each well of the plate followed by 10 µL of standard or unknown sample in technical duplicate. The plate was sealed with a plate sealer and incubated on a plate shaker for 2 hrs at room temperature. The plate was then washed four times with wash buffer and 100 µL of biotinylated goat anti-human AAT polyclonal antibody diluted in multibuffer was added to the plate. The plate was sealed with a plate sealer and incubated on a plate shaker for 2 hrs at room temperature. The plate was then washed four times with wash buffer and 100 µL of streptavidin-europium conjugate (PerkinElmer) diluted in multibuffer was added to the plate. The plate was sealed with a plate sealer and incubated on a plate shaker for 30 minutes at room temperature. The plate was then washed six times with wash buffer and 200 µL of enhancement solution (PerkinElmer) was added to the plate. The plate was incubated on a plate shaker for 5 minutes followed by 5 minutes on the bench before reading time-resolved fluorescence in the Victor^3^ plate reader (PerkinElmer). Results were calculated using the PerkinElmer MultiCalc software package.

#### Albumin electrochemiluminescence immunoassay

Albumin was measured using the MesoScale Discovery assay platform. Mesoscale standard bind plates were coated with a goat anti-human albumin polyclonal antibody (Bethyl laboratories) diluted in PBS. The plate was incubated overnight and washed three times with PBS/Tween wash buffer before use. The assay was calibrated with a human serum preparation (Multiqual; Biorad). The preparation was diluted in MSD Diluent 7 (MesoScale Discovery) to produce a series of 8 standards with a concentration range of 1000 to 15.6 µg/L. MSD Diluent 7 was used as the zero concentration standard. 30 µL of MSD Diluent 7 was added to each well of the plate followed by 10 µL of standard or unknown sample in technical duplicate. The plate was sealed with a plate sealer and incubated on a plate shaker for 2 hrs at room temperature. The plate was then washed three times with PBS/Tween wash buffer and 25 µL of rabbit anti-human albumin polyclonal antibody diluted in MSD Diluent 100 was added to the plate. The plate was sealed with a plate sealer and incubated on a plate shaker for 1 hr at room temperature. The plate was then washed three times with PBS/Tween wash buffer and 25 µL of goat anti-rabbit IgG-SulphoTAG diluted in MSD Diluent 100 was added to the plate. The plate wa sealed with a plate sealer and incubated on a plate shaker for 30 minutes at room temperature. The plate was then washed three times with PBS/Tween wash buffer and 150 µL of 1X Read Buffer T (MesoScale Discovery) was added to the plate. The plate was read immediately on a SECTOR Imager 6000. Results are calculated using the MSD Discovery Workbench software package.

#### Alpha-fetoprotein DELFIA

AFP was quantified using the commercially available DELFIA hAFP kit and protocol (PerkinElmer).

### Statistical Analyses

Standard error measurements and sample means were calculated for all conditions and subjected to unpaired, two-tailed, Welch’s t-tests. P-values below 0.05 were considered significant for this study. Hierarchical clustering was performed using Euclidean distances with unweighted pair-group methods using centroids.

### Calculation of Average Global Change in Fold Expression

Average change of a culture condition in fold expression for the 39 genes analyzed in aggregate compared to the 2D Progenitor Culture was calculated according to the following formula:
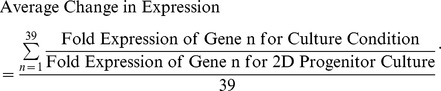



## Results and Discussion

### Cell-cell Junctions are Necessary for the Maintenance of the Hepatic Phenotype in 3D

**Figure 1:**
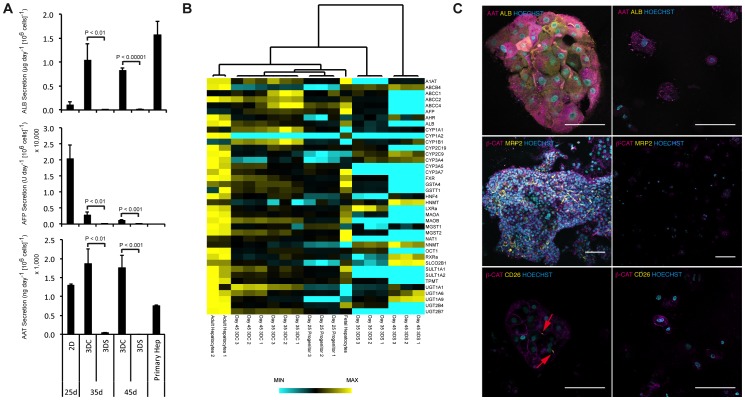
Functional and transcriptional comparison of IPSC-Hep 3D cultures plated as single cells or clumps. (**A**) Secreted albumin, alpha-fetoprotein, and alpha-1-antitrypsin levels as evaluated by immunoassays (mean ± s.d.; n = 3 biological replicates). (**B**) qPCR heatmap of 39 hepatic genes comparing the two 3D culture conditions to adult and fetal hepatocytes (range of expression shown as sample extrema for each gene; quantitative values shown in **Figures S2–S4**). (**C**) Confocal micrograph highlighting the loss of detectable albumin in 3D single cell cultures and the spontaneous polarization of IPSC-Heps within 3D clump cultures (scalebar = 100 microns).

We began by investigating the importance of cell-cell junction maintenance during the transfer of the cells from 2D to 3D culture (**Figure 1**). Media samples were taken at day 25, 35, and 45 and were subjected to immunoassays in order to quantify the secretion of human serum albumin, alpha-1-antitrypsin (A1AT), and alpha-fetoprotein (AFP), which marks specifically fetal hepatocytes. At day 45, 3D clump cultures demonstrated a 10-fold increase in albumin secretion, a 1.5-fold increase in A1AT secretion, and a 20-fold decrease in AFP secretion compared to the day 25 common progenitor (**Figure 1a**). Conversely, 3D single cell cultures demonstrated a 10-fold decrease in albumin secretion and a complete loss of detectable A1AT. Furthermore, AFP secretion decreased by 1500-fold in single cells suggesting a general decline in hepatic phenotype. Increasing the density of single cell cultures to mimic the local cell density within clumps had no significant effect on the phenotype (data not shown). Together these data show that cell-cell junction maintenance is necessary for Heps-IPSC differentiation and that 3D culture could accelerate the decrease of fetal markers such as AFP.

To confirm these observations, we compared the maturation and hepatic phenotypic profile of IPSC-Heps to that of freshly isolated adult hepatocytes by qPCR analyses of 39 hepatic genes, including multiple phase I/II/III metabolic enzymes along with several hepatic nuclear receptors. Hierarchical clustering of the profiles shows a distinct divergence in the two culture conditions that increases with time. By day 45, the 3D single cell condition lost detectable expression of 23 out of the 39 genes analyzed, with an average gene expression of 0.0001% of primary adult hepatocytes, nearly an 8,000-fold decrease from the day 25 common progenitor (**Figures 1b** **, S2a–c, S3a–c, S4a–c, S5**). In contrast, the 3D clump culture experienced an average 6-fold increase compared to the progenitor and expressed all 39 genes analyzed to varying degrees. Finally, localization and homogeneity of protein expression (AAT, ALB, β-CAT, MRP2 and CD26) were assessed by immunofluorescence. 3D clump cultures demonstrated a homogenous population of cells with protein expression and localization similar to that seen in PHHs, whereas 3D single cell cultures demonstrated significant heterogeneity in expression (**Figures 1c** **, S7, S8**). Additionally, significant irregularities in cell size and nucleus morphology along with membrane blebbing were seen in the single cell cultures. This is suggestive of contact-dependent apoptosis similar to that seen in low density PHH dedifferentiation [1], [2].

### 3D Clump Cultures Induce a more Mature Phenotype Compared to 2D

**Figure 2:**
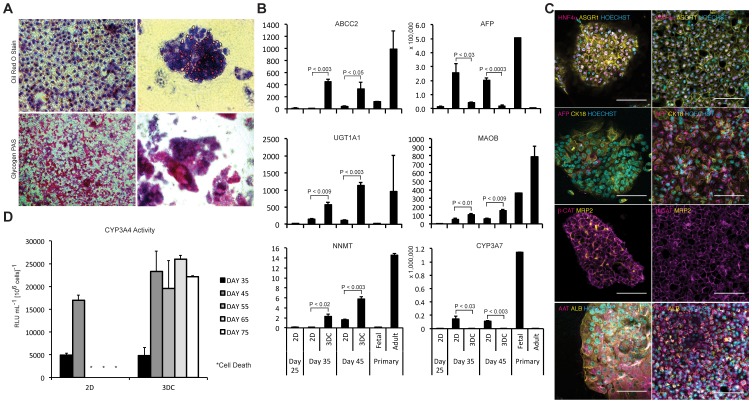
Functional comparison of IPSC-Hep 3D clump culture versus traditional 2D culture. (**A**) Oil red O and periodic acid staining demonstrating lipid storage and glycogen synthesis in both 2D and 3D clump cultures. (**B**) qPCR analysis of select phase I and phase II enzymes, hepatic transporters, and other hepatic markers demonstrating a shift towards a more mature phenotype in the 3D clump cultures (fold expression to undifferentiated IPSCs; mean ± s.d.; n = 3 biological replicates). (**C**) Confocal micrographs comparing the presence and localization of hepatic markers within the two culture systems (scale bar = 100 microns). (**D**) CYP3A4 activity of the two culture conditions measured over a period of 75 days (mean ± s.d.; n = 3 biological replicates).

Having confirmed the necessity of cell-cell junctions in phenotypic maintenance, we conducted a direct comparison of the 3D clump culture to 2D controls in order to determine the functional benefits which 3D culture could confer. We began with oil red o and periodic acid staining to determine differences in lipid storage and glycogen synthesis respectively (**Figure 2a**). Both cultures demonstrated the ability to store lipids and synthesize glycogen; however, the 3D clump culture demonstrated a significantly higher percentage of cells actively synthesizing glycogen (>95% compared to 46% in 2D).

Furthermore, qPCR analysis using the 39 gene panel described above demonstrated significant maturation events in Phase I/II/III enzymes in addition to other hepatocyte associated genes (**Figures 2b** **, **, S2a–c, S3a–c, S4a–c, S5****). *AFP* and *CYP3A7*, both markers of fetal hepatocytes [17], [18], were decreased 20-fold and 140-fold respectively in the day 45 3D clump culture compared to the day 45 2D control (**Figure 2b**). *MAOB* (Phase I), *UGT1A1* (Phase II), *NNMT* (Phase II), and *ABCC2* (Phase III) were increased 2.5-fold, 10-fold, 3.7-fold, and 7.3-fold respectively (**Figure 2b**).

The induction of *ABCC2*, a marker of hepatocyte polarity found on the apical pole and bile canalicular surfaces [19], led us to investigate cell polarity further. Immunostaining demonstrated the presence of extensive canalicular formation throughout the 3D clump cultures (as demonstrated by ABCC2 and CD26 [20]) and an absence within the 2D controls. (**Figures 1c** **,** **2c** **, S6–S9, Videos S1–S4**). The establishment and maintenance of IPSC-Hep polarity in 3D culture mediated through integrin-matrix interactions is consistent with previous findings with primary hepatocytes and has been shown to significantly decrease dedifferentiation and increase longevity in these cells [1], [2], [21], [22].

In order to assess any changes in functional longevity associated with the 3D system, CYP3A4 activity was measured every 10 days throughout the study using luciferase-based assays. No significant differences were found in CYP3A4 activity between the two culture conditions at day 35 or 45 (**Figure 2d**). However, between days 45 and 55, cells in 2D culture consistently formed large vacuoles and subsequently detached from culture surface. In contrast, cells within the 3D matrix maintained levels of CYP3A4 activity at approximately 25% of that of PHHs (**Figures 2D** and **S10**; n = 35 primary samples; range 10%–200% activity of individual primary samples; interpolated from HPLC-MS to P450-Glo) from day 45 through 75. Although no further maturation was observed during this period, we observed no significant loss in CYP3A4 activity, demonstrating that the RAFT system is conducive with long-term maintenance of cytochrome activity. Our analysis ceased at day 75; however, cells could potentially maintain functionality for even longer periods, making this method ideal for long-term experiments needed for physiologically relevant toxicology studies.

## Conclusion

In summary, we have presented a method to easily improve the maturation of current IPSC-Hep lines simply by transferring existing cells as epithelial clumps into 3D collagen matrices. We have demonstrated that transition to 3D culture while maintaining cell-junctions significantly shifts cell phenotype towards that of primary hepatocytes compared to traditional 2D culture. Additionally, 3D clump culture induces polarization and bile canaliculi formation and extends the functional lifetime of the cells to over 75 days. Although further development is needed to generate fully functional cells, our work represents a significant step for the development of 3D systems for modeling liver diseases and testing the toxic effects of various xenobiotics and suggests that this method may be widely applicable to increase IPSC-Hep maturity.

## Supporting Information

Figure S1 **Method to differentiate IPSC-Hep in 3D.** (**a**) Schematic of the RAFT process used in the maturation of IPSC-Heps. (**b**) Scanning electron micrograph of 3D clump culture (scalebar = 5 microns). (**c**) Outline of the experiment used to probe the effects of the three culture conditions on the maturation of IPSC-Heps.
(TIF)

Figure S2 **qPCR analysis for BOB5 SC.** (**a–c**) Fold expression to undifferentiated IPSCs; mean ± s.d.; n = 3 biological replicates.
(PDF)

Figure S3 **qPCR analysis for BOB7 RM.** (**a–c**) Fold expression to undifferentiated IPSCs; mean ± s.d.; n = 3 biological replicates.
(PDF)

Figure S4 **qPCR analysis for BBHX8.** (**a–c**) Fold expression to undifferentiated IPSCs; mean ± s.d.; n = 3 biological replicates.
(PDF)

Figure S5 **qPCR heatmap.** Heatmap of 39 hepatic genes comparing all conditions and lines investigated in this study (fold expression normalized to adult hepatocytes).
(TIFF)

Figure S6 **Canalicular structures.** Orthogonal analysis of 3D clump cultures demonstrating the presence of canalicular buds (green – ASGPR, Red – HNF4a, blue Hoechst).
(TIFF)

Figure S7 **Confocal micrographs.** Single channel and merged confocal micrographs of images shown in **Figure 1** (scalebar = 100 microns).
(TIFF)

Figure S8 **Confocal micrographs.** Single channel and merged confocal micrographs of images shown in **Figure 2** (scalebar = 100 microns).
(TIFF)

Figure S9 **Canalicular structures.** Confocal micrographs demonstrating the presence and localization of bile canaliculi and canalicular buds within the 3D clump cultures (scalebar = 100 microns).
(TIF)

Figure S10 **Cytochrome P450 Activity.** CYP3A4 activity of the 2D progenitor as assessed by the rate of conversion of Midazolam to 1′-HO-Midazolam using HPLC-MS (n = 35 primary samples; range 10%–200% activity of individual primary samples).
(PDF)

Table S1 List of primer sequences used for qPCR analyses.
(PDF)

Table S2 P-Values for Welch’s T-test used to determine significance of BBHX8 qPCR analyses.
(PDF)

Table S3 P-Values for Welch’s T-test used to determine significance of BOB5 SC qPCR analyses.
(PDF)

Table S4 P-Values for Welch’s T-test used to determine significance of BOB7 RM qPCR analyses.
(PDF)

Table S5 Significance of BBHX8 qPCR analyses by Welch’s T-test.
(PDF)

Table S6 Significance of BOB5 SC qPCR analyses by Welch’s T-test.
(PDF)

Table S7 Significance of BOB7 RM qPCR analyses by Welch’s T-test.
(PDF)

Video S1 **3D Canalicular structure.** Z-stack of a 3-D clump culture demonstrating the presence of canalicular bud formation (scalebar = 100 microns; green – ASGPR, Red – HNF4a, blue Hoechst).
(MOV)

Video S2 **3D Canalicular structure.** Z-stack of a 3-D clump culture demonstrating the presence and localization of bile canaliculi and canalicular buds (green – MRP2, Red – *β* -Cat, blue Hoechst).
(MOV)

Video S3 **3D Canalicular structure.** Z-stack of a 3-D clump culture demonstrating the presence and localization of bile canaliculi and canalicular buds (scalebar = 50 microns; green – MRP2, blue Hoechst).
(MOV)

Video S4 **3D Canalicular structure.** Z-stack of a 3-D clump culture demonstrating the presence and localization of bile canaliculi and canalicular buds (scalebar = 50 microns; green – MRP2, blue Hoechst).
(MOV)
