# Supplementary figures and images for: Maturation of Induced Pluripotent Stem Cell Derived Hepatocytes by 3D-Culture
Source: PLoS One. 2014 Jan 22;9(1):e86372. doi: 10.1371/journal.pone.0086372 (PMC3899231; doi:10.1371/journal.pone.0086372)

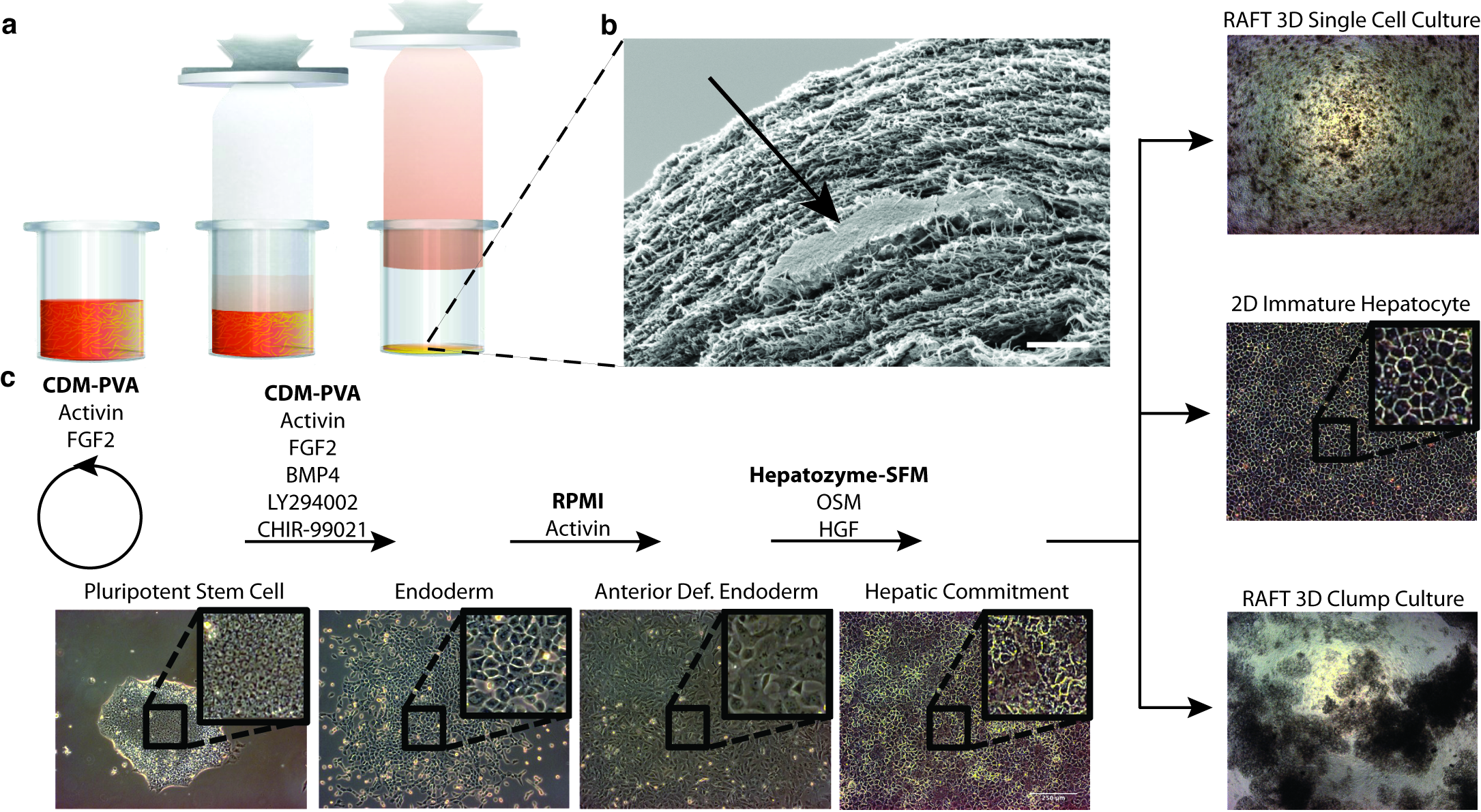

Supplement: Figure S1 — Method to differentiate IPSC-Hep in 3D. (a) Schematic of the RAFT process used in the maturation of IPSC-Heps. (b) Scanning electron micrograph of 3D clump culture (scalebar = 5 microns). (c) Outline of the experiment used to probe the effects of the three culture conditions on the maturation of IPSC-Heps. (TIF) [file pone.0086372.s001.tif]

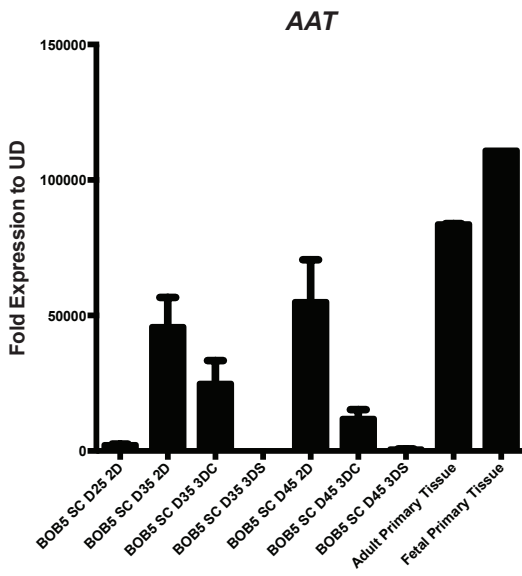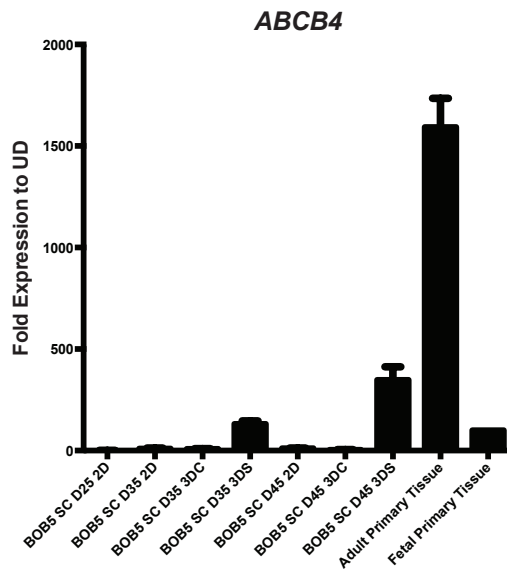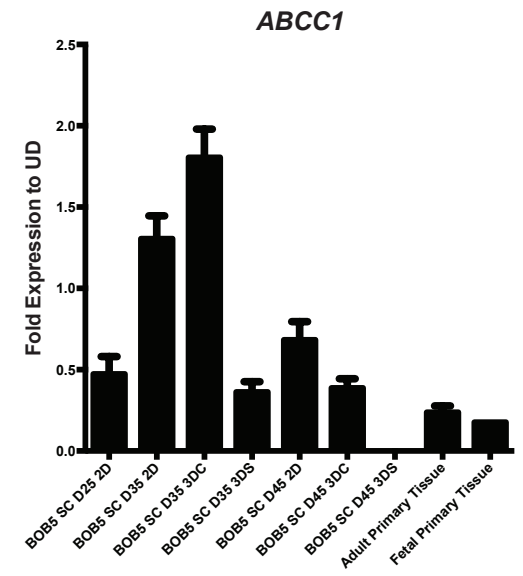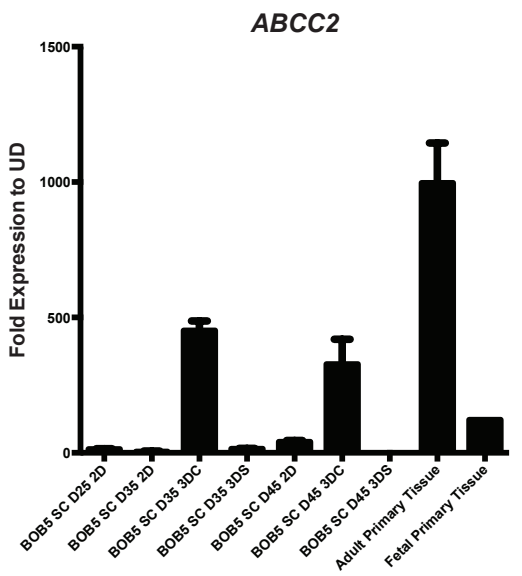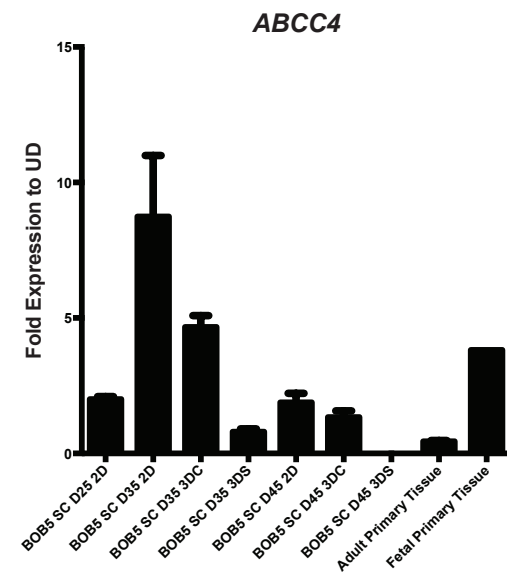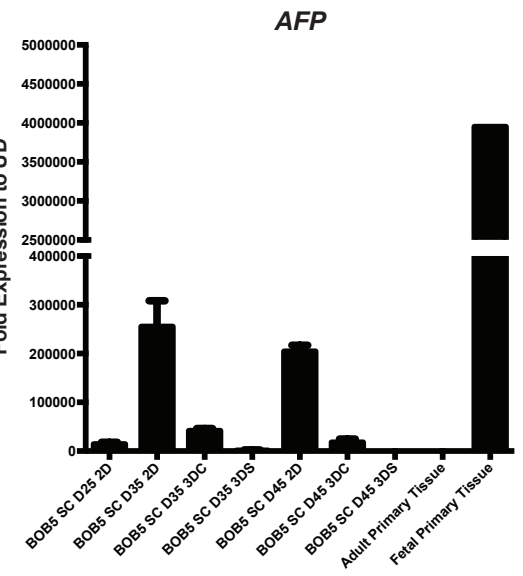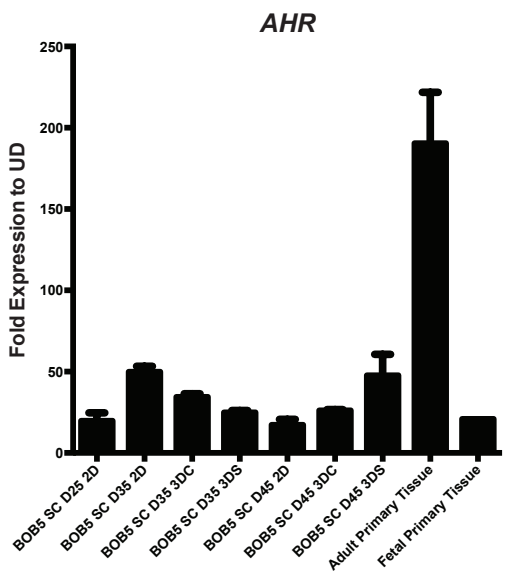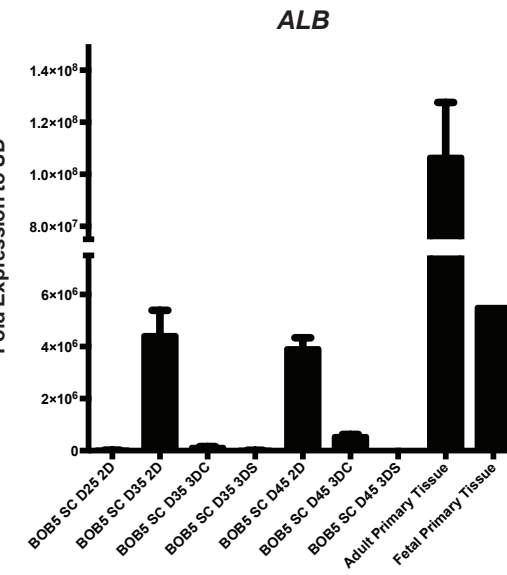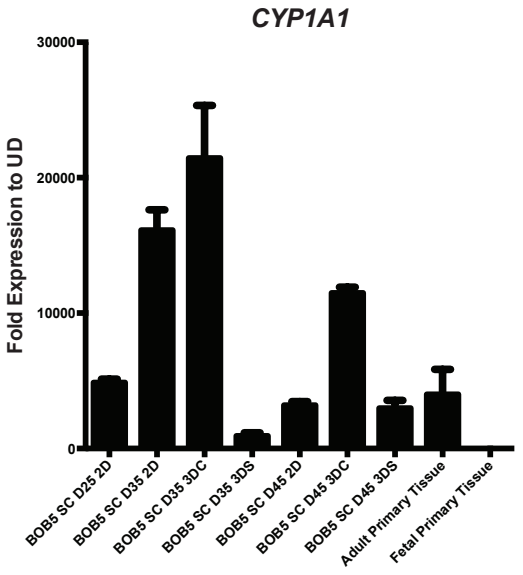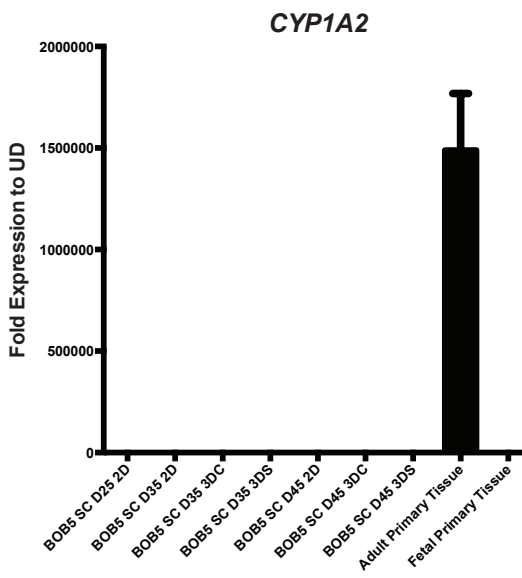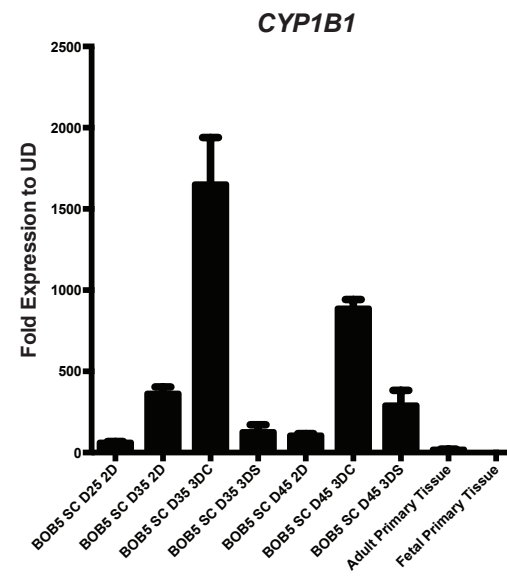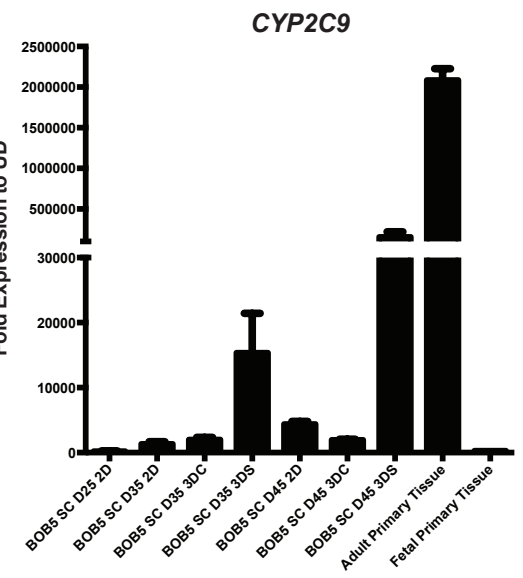

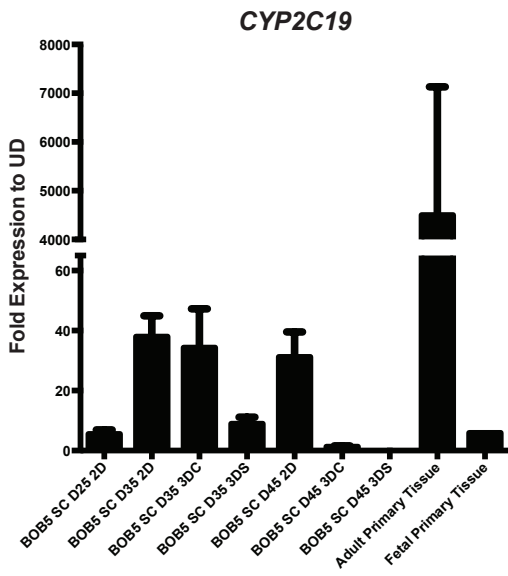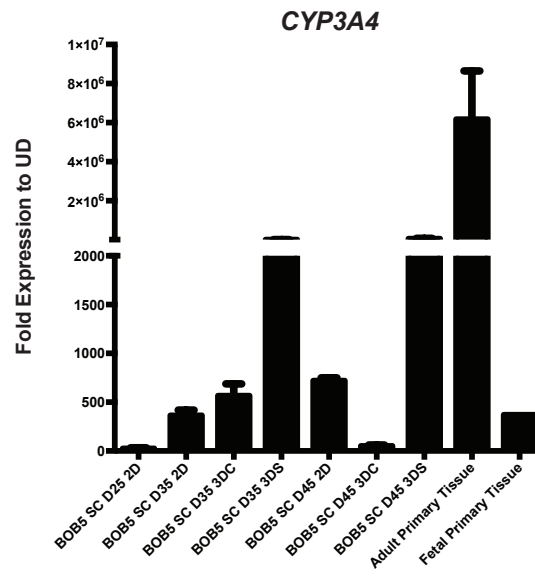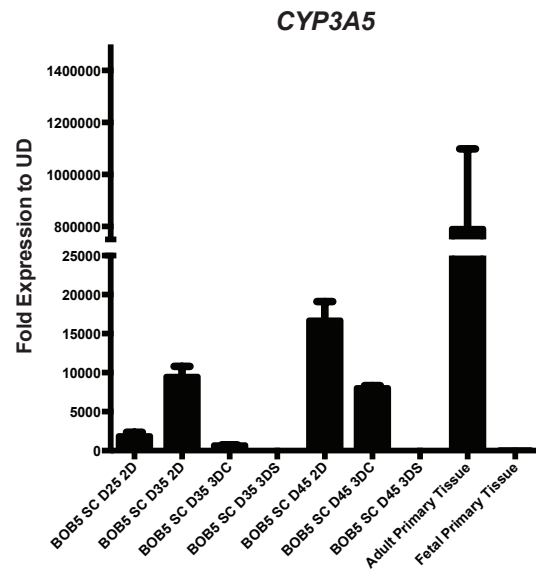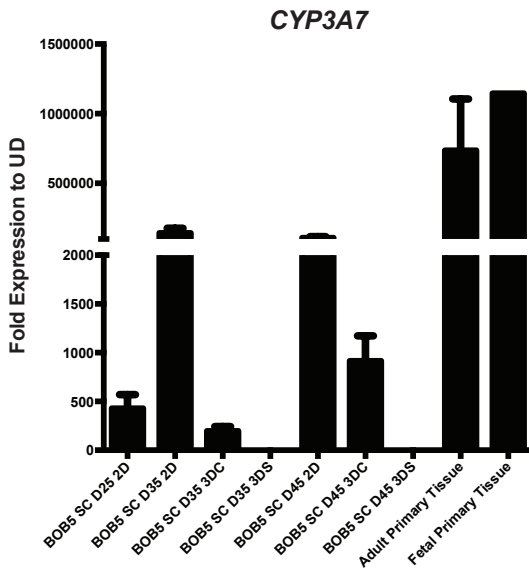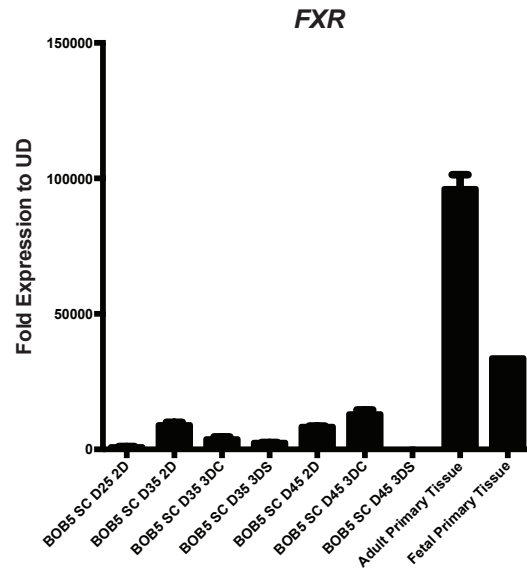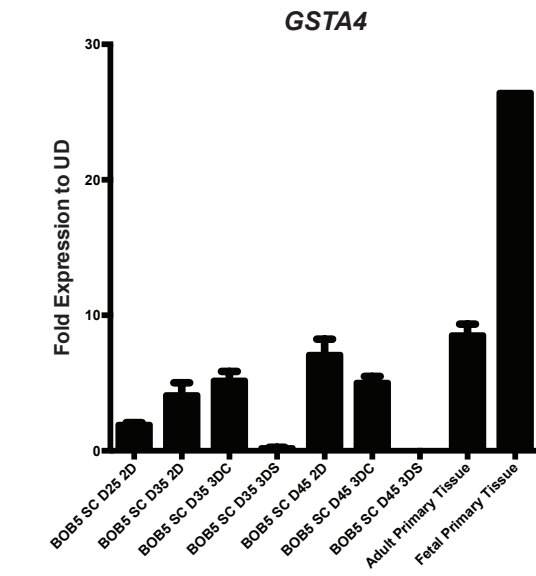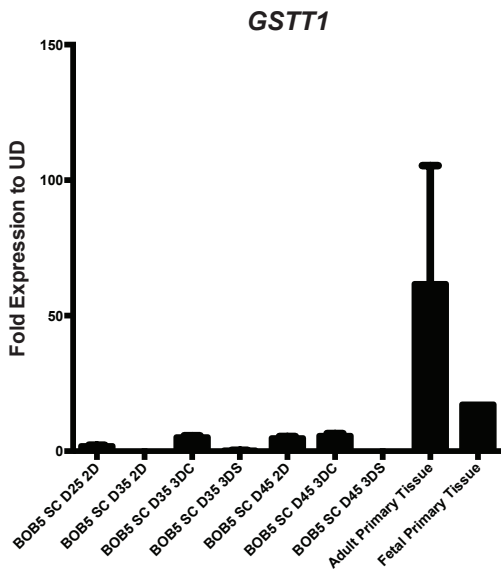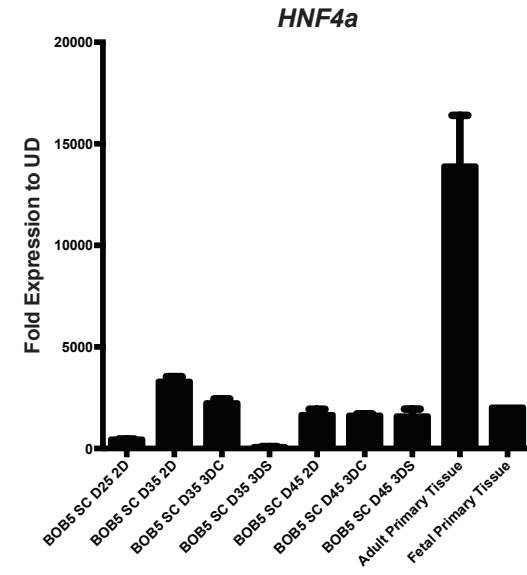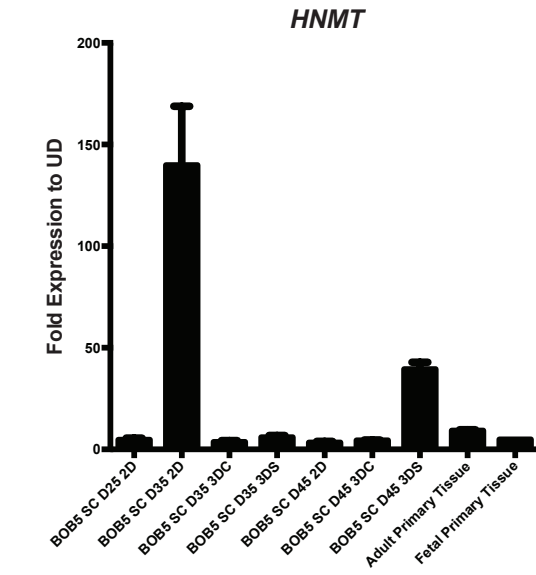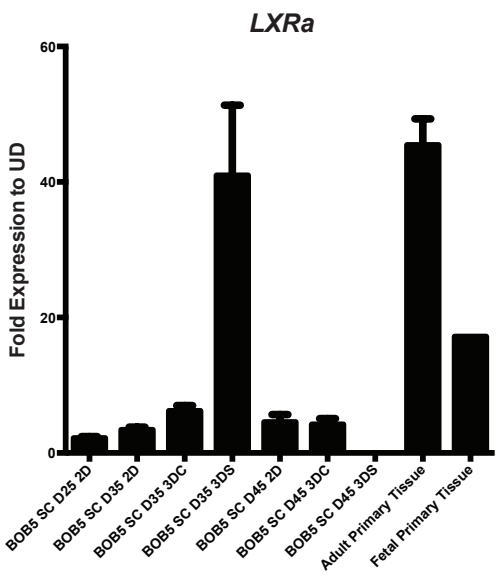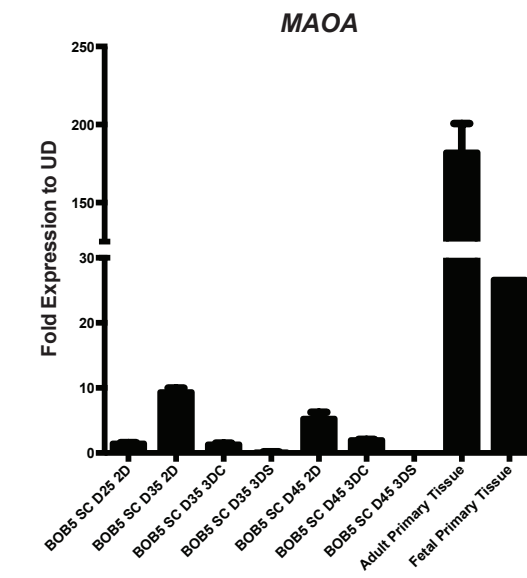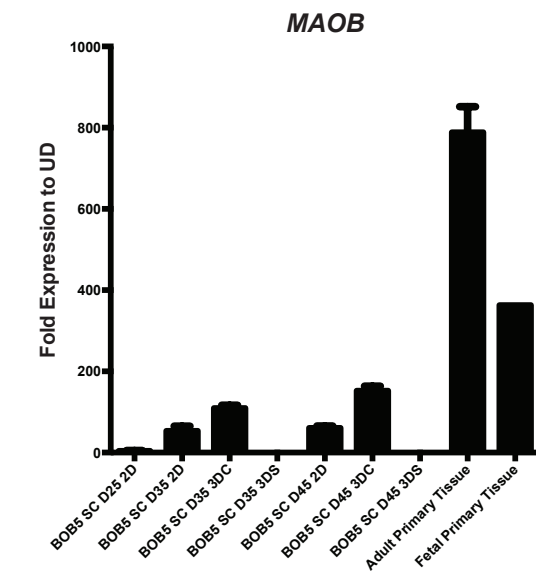

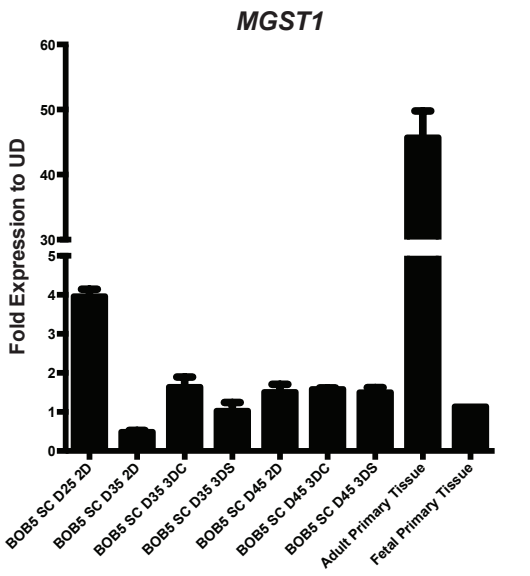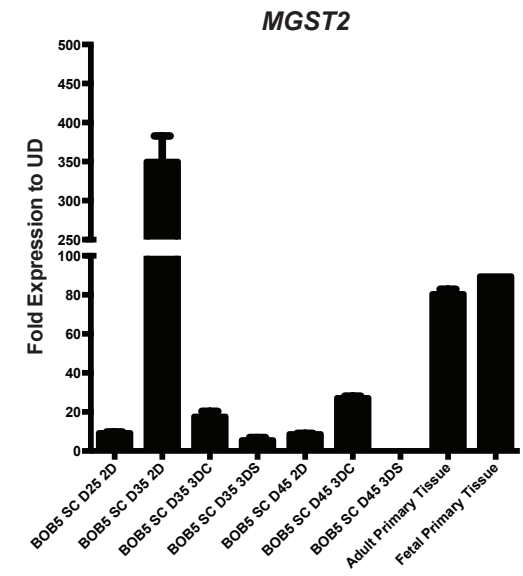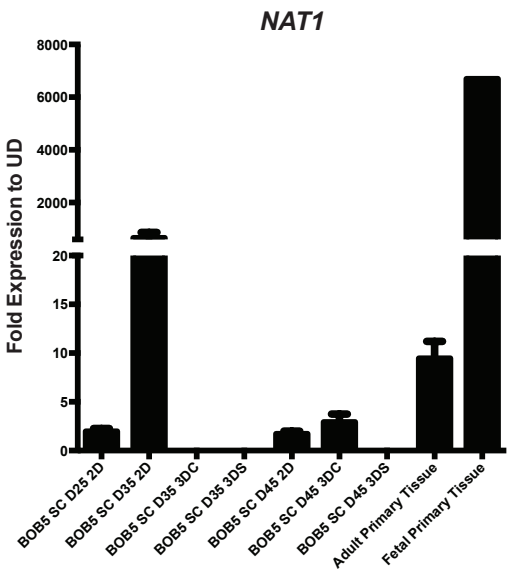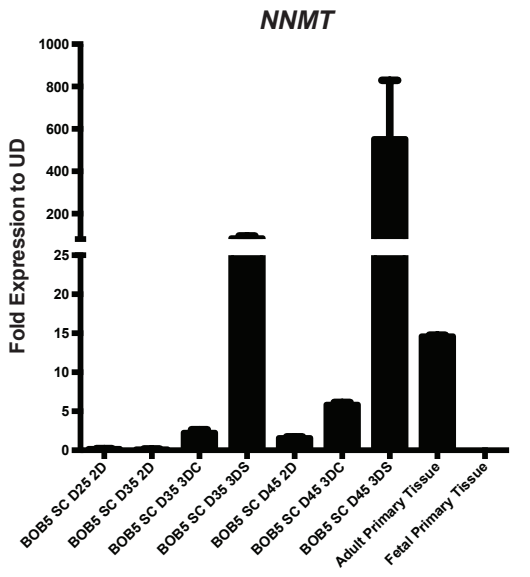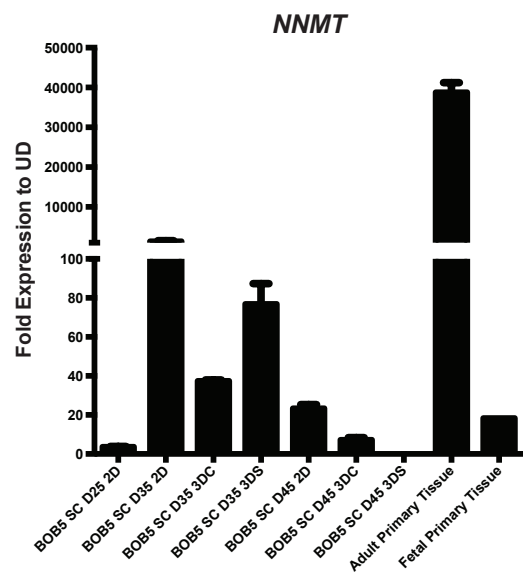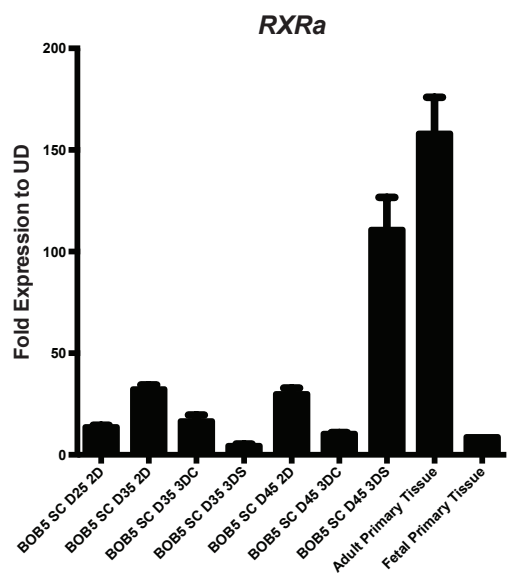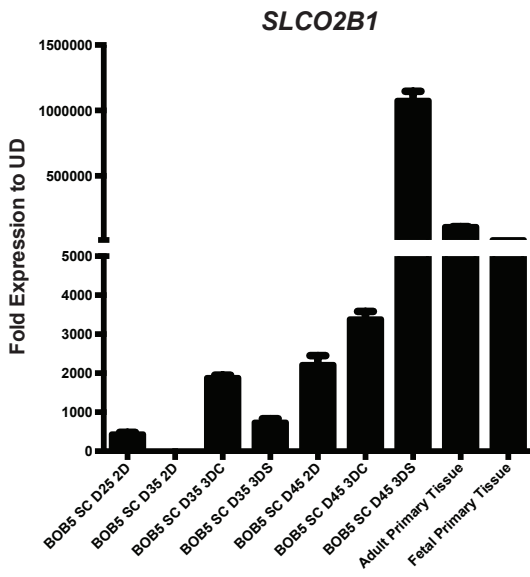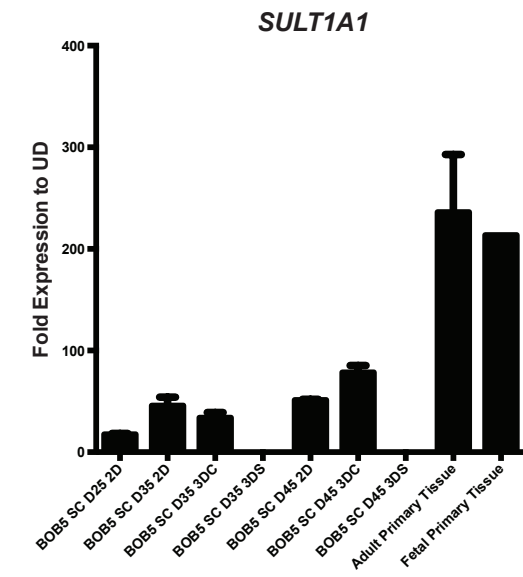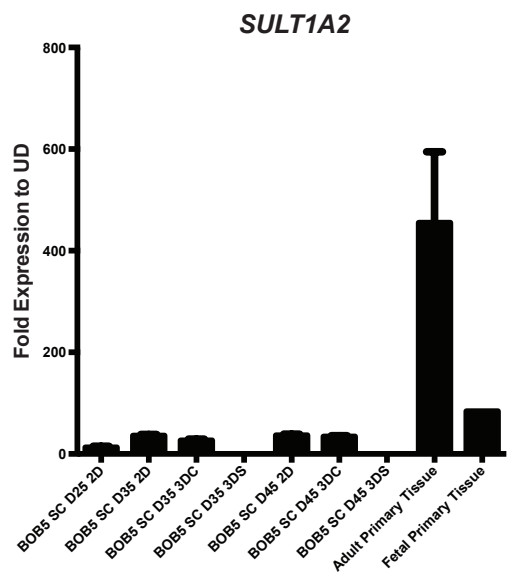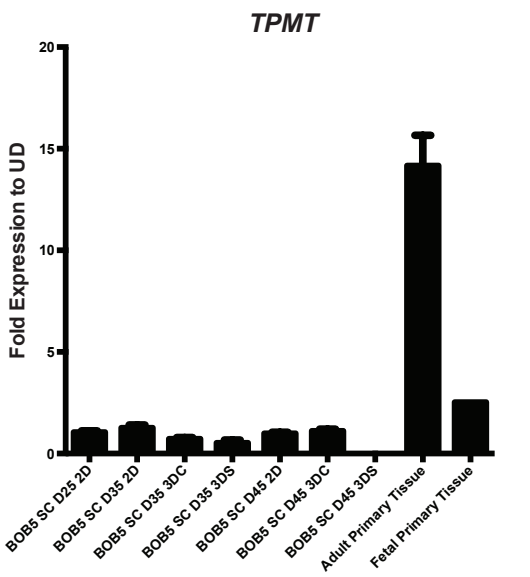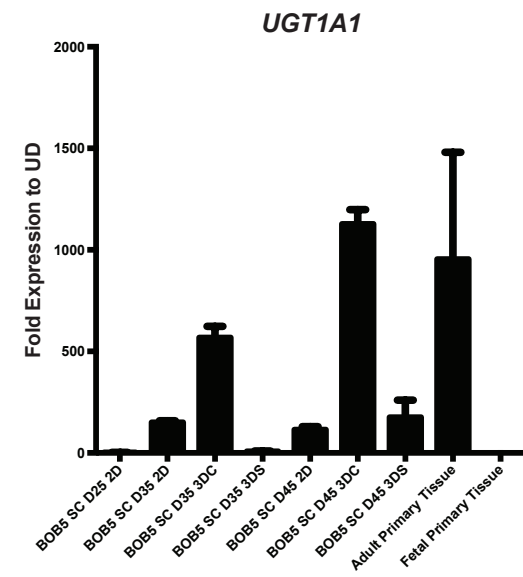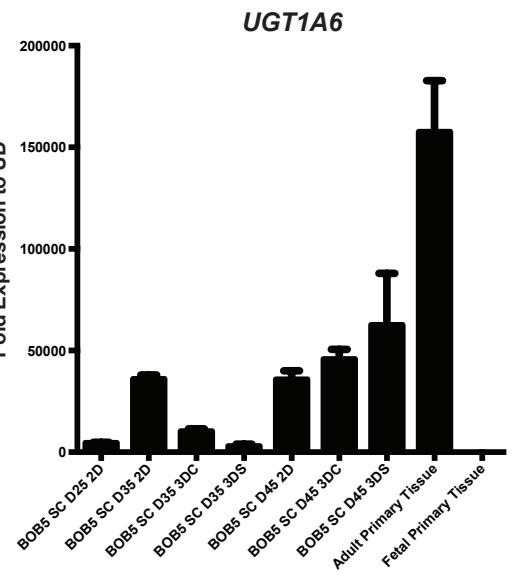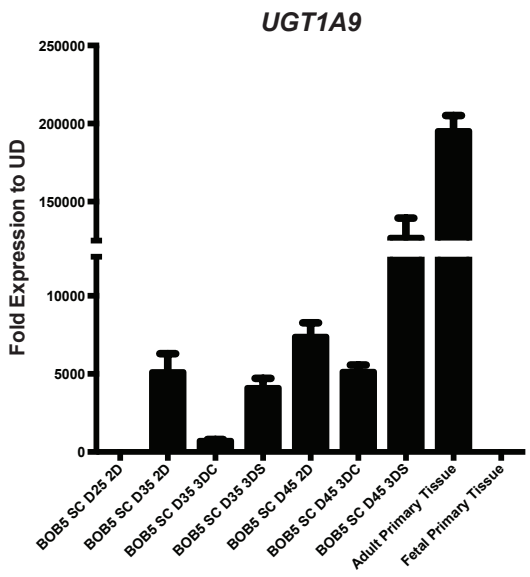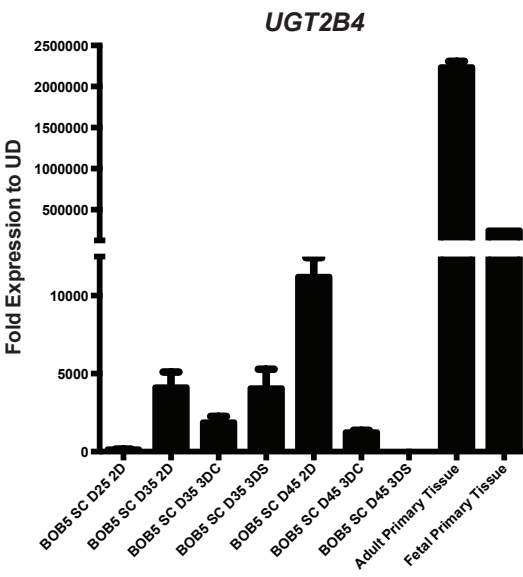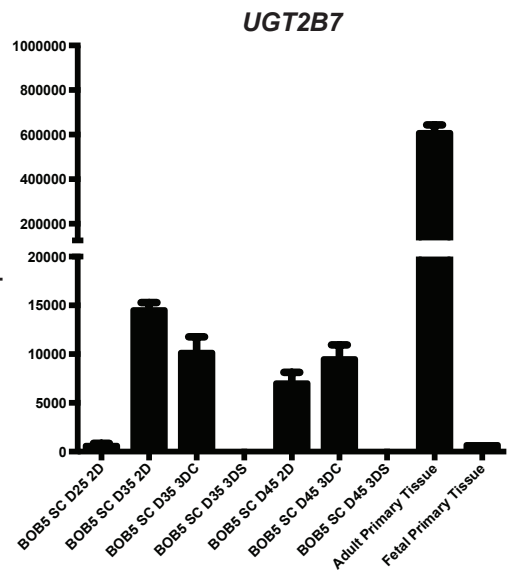

Supplement: Figure S2 — qPCR analysis for BOB5 SC. (a–c) Fold expression to undifferentiated IPSCs; mean ± s.d.; n = 3 biological replicates. (PDF) [file pone.0086372.s002.pdf]

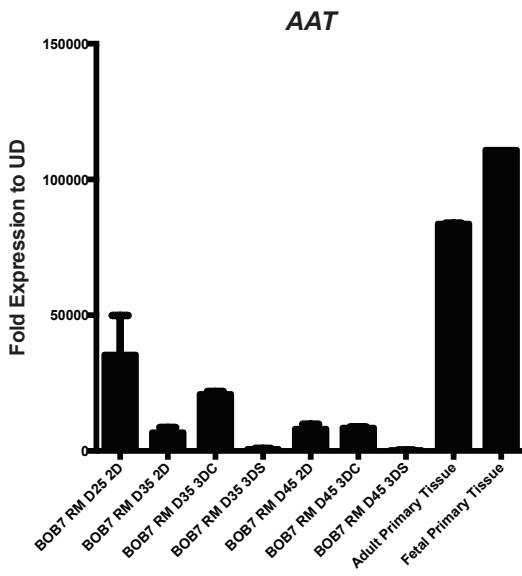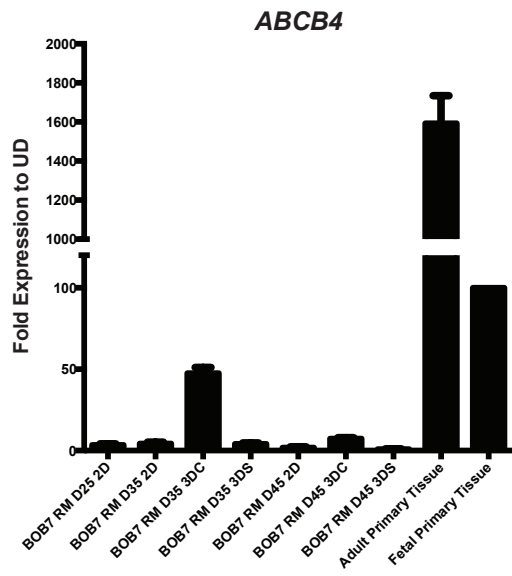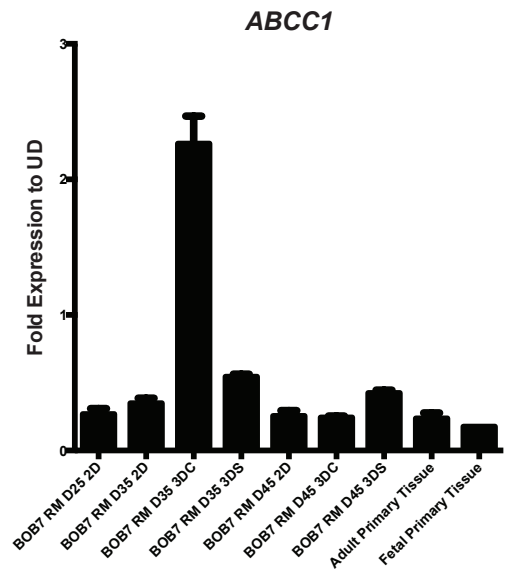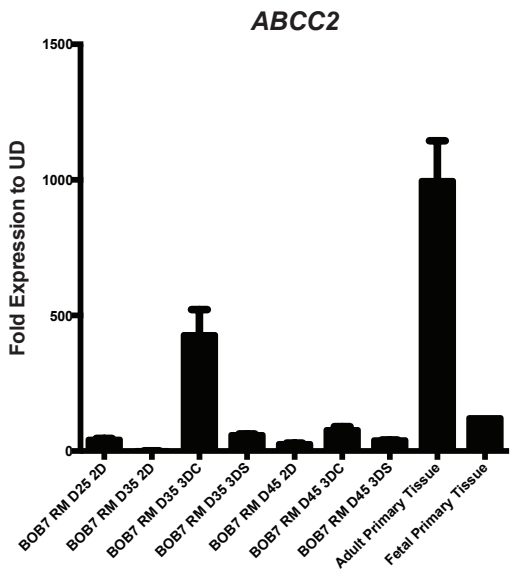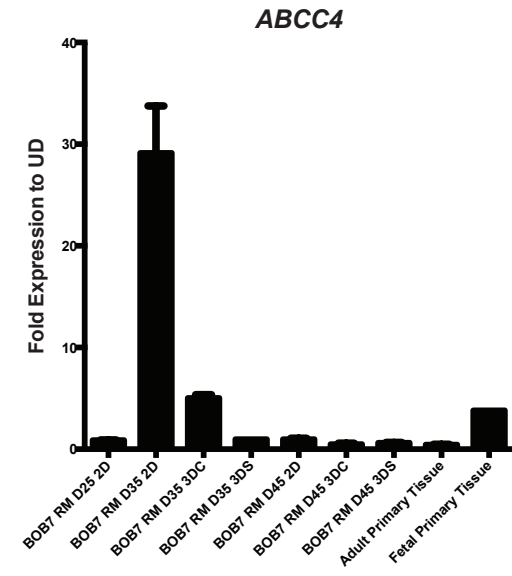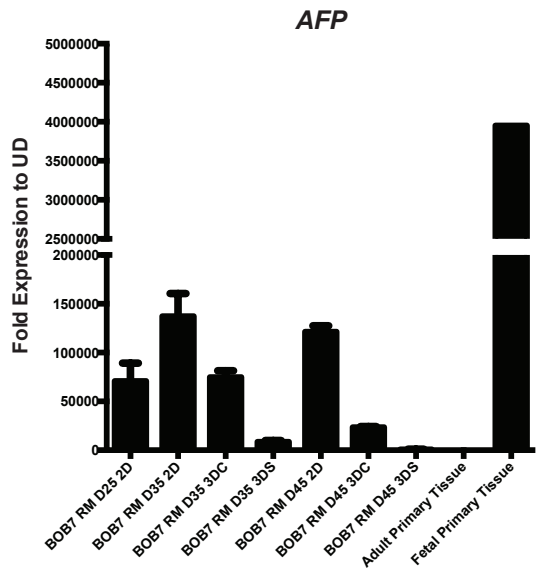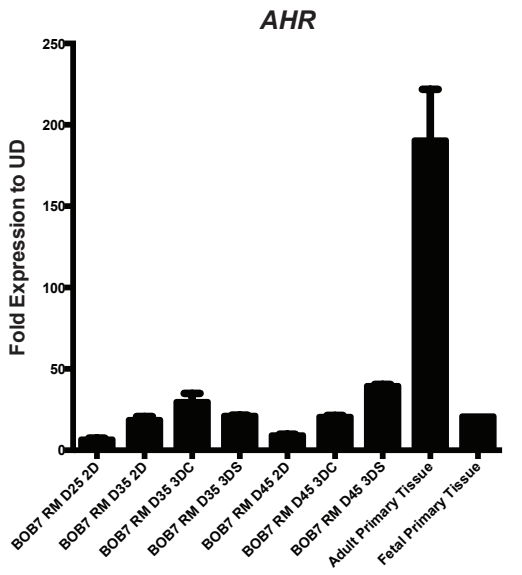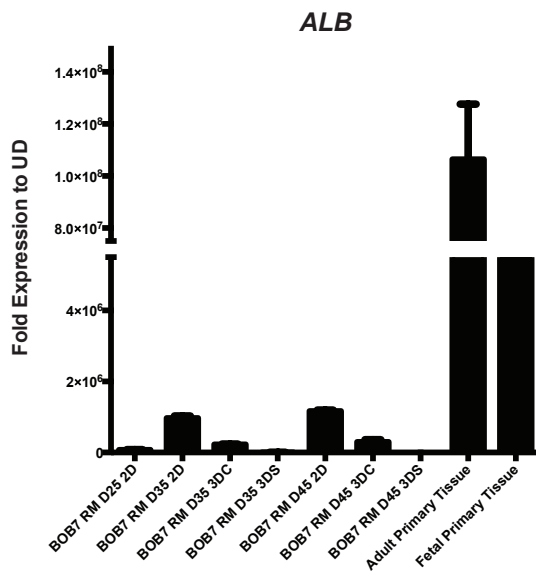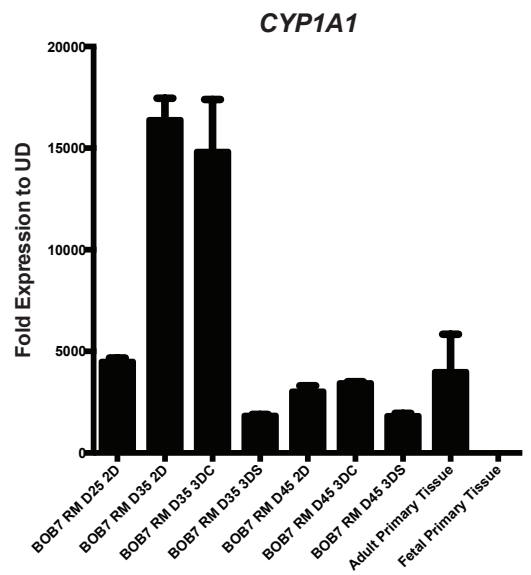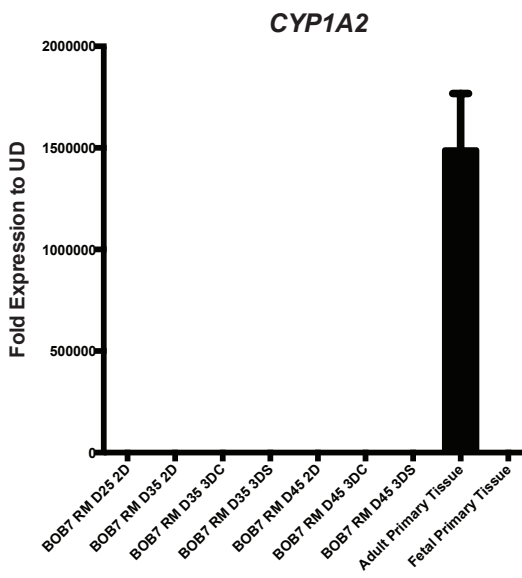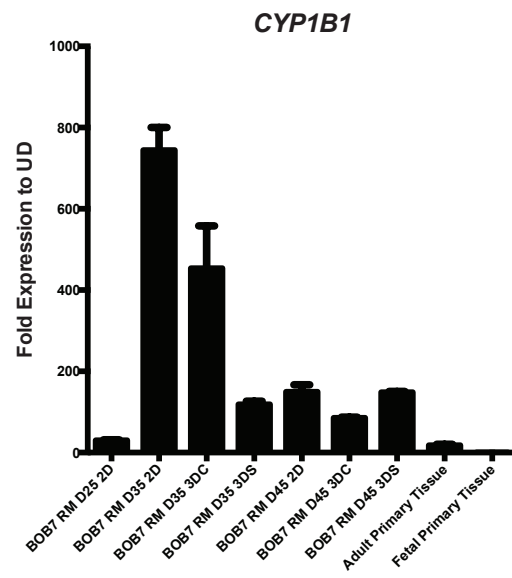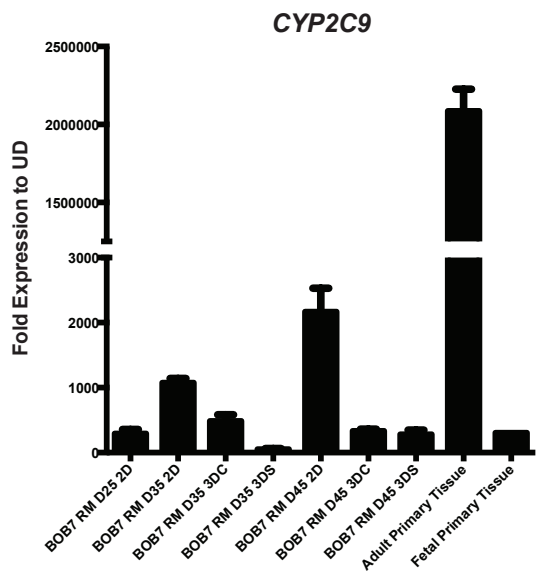

**CYP2C19**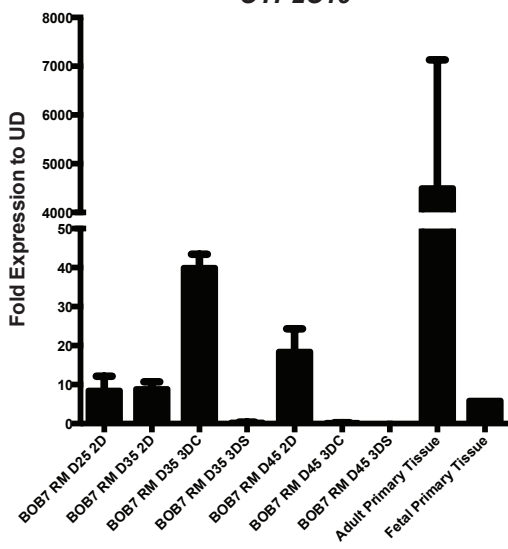**CYP3A4**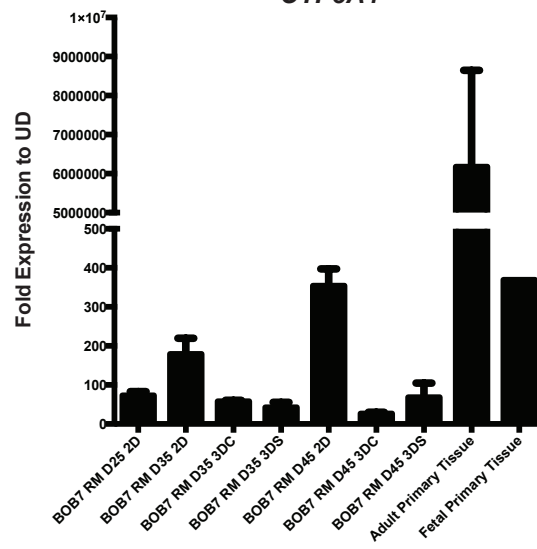**CYP3A5**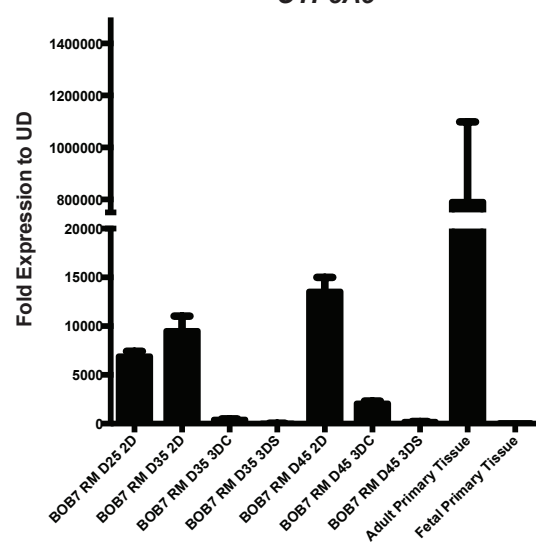**CYP3A7**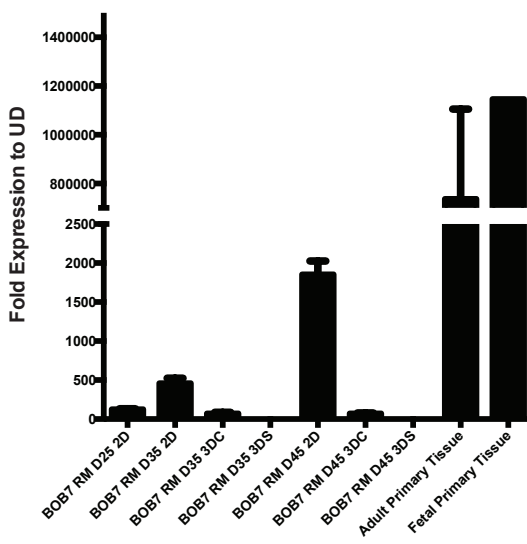**FXR**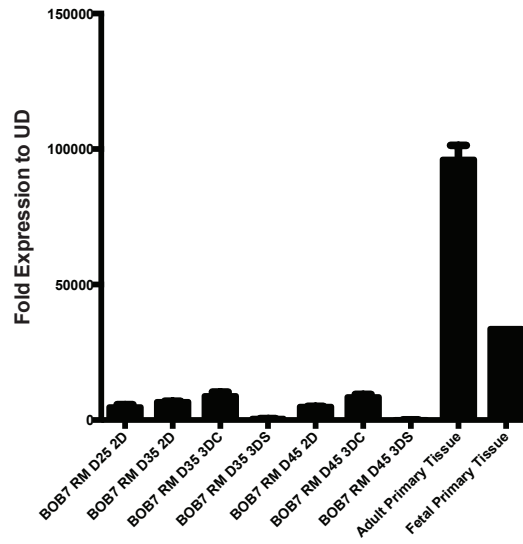**GSTA4**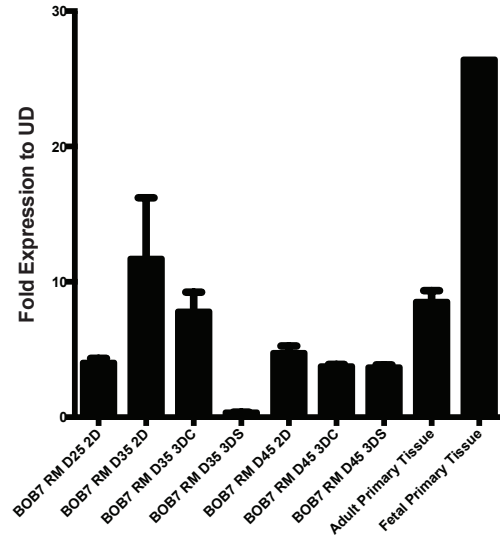**GSTT1**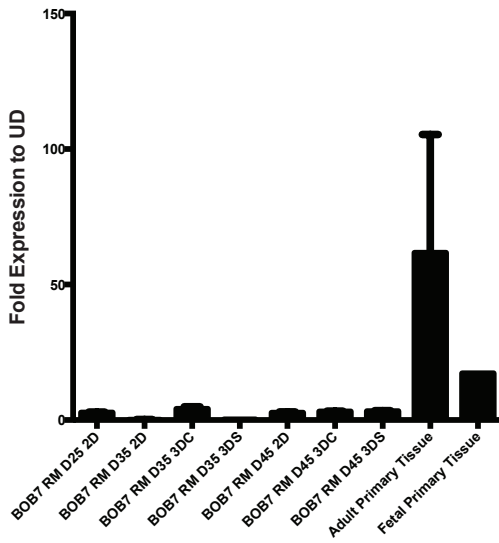**HNF4a**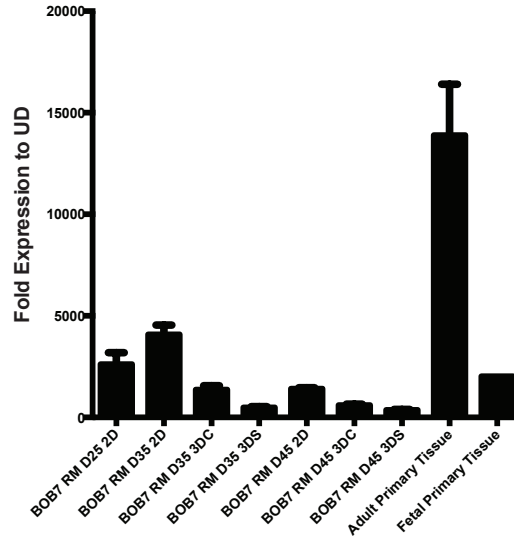**HNMT**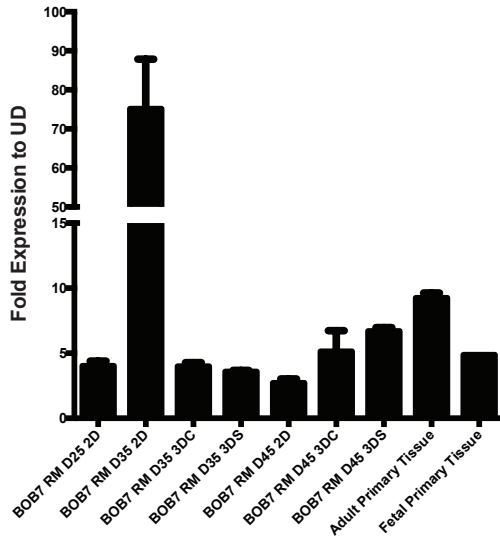**LXRa**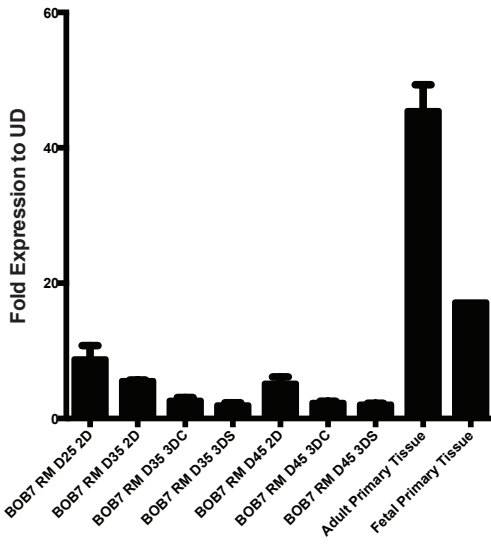**MAOA**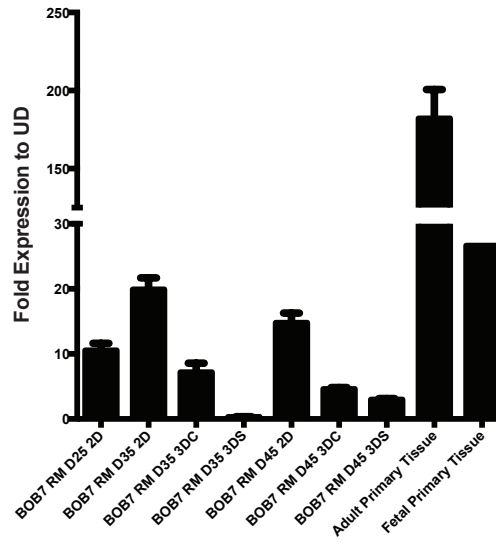**MAOB**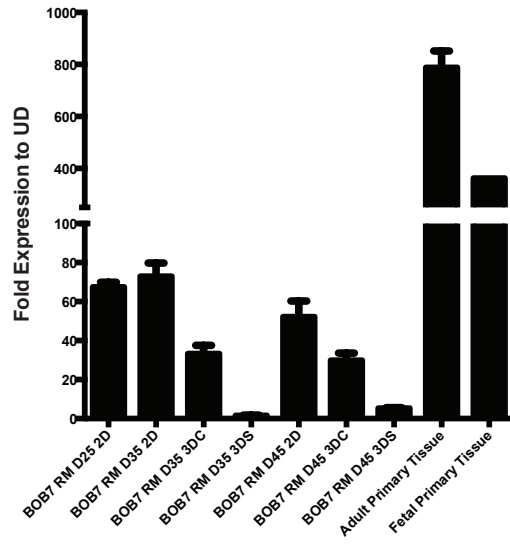

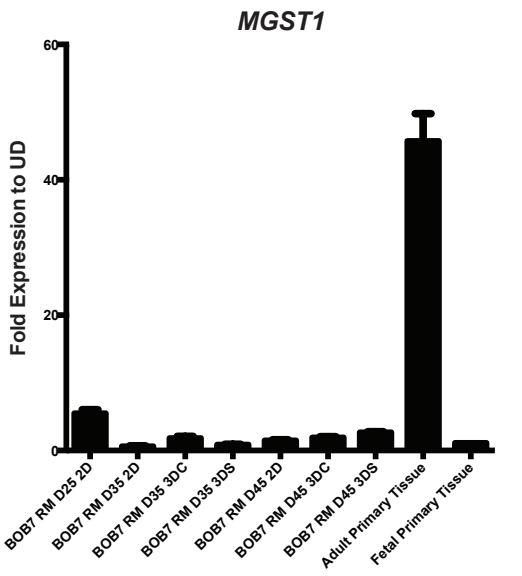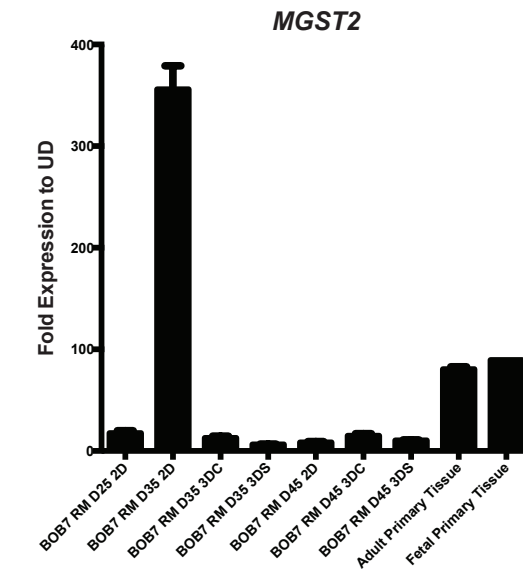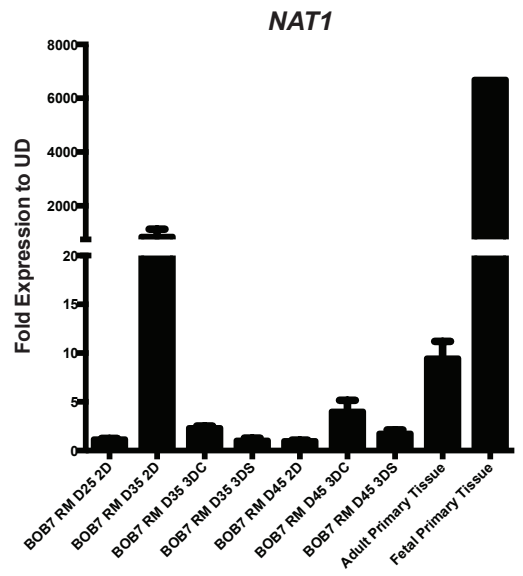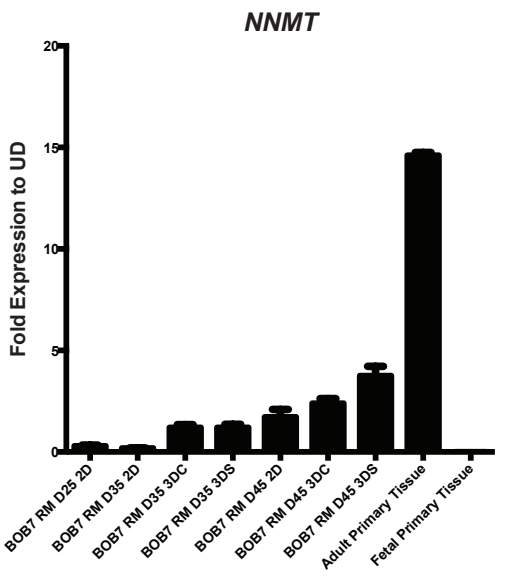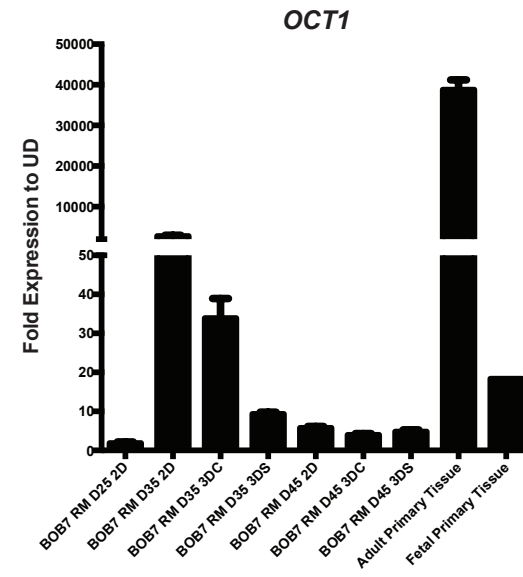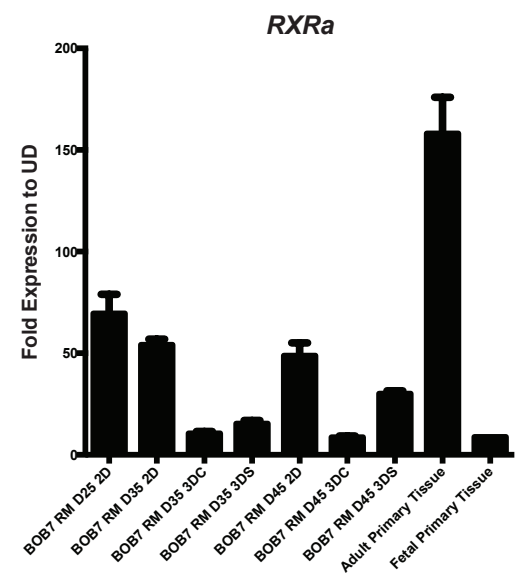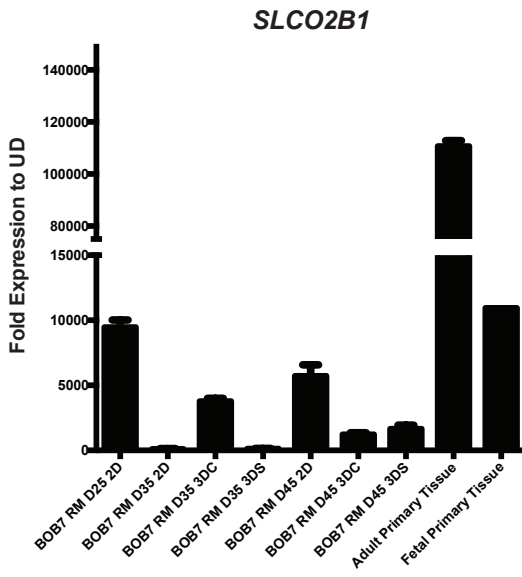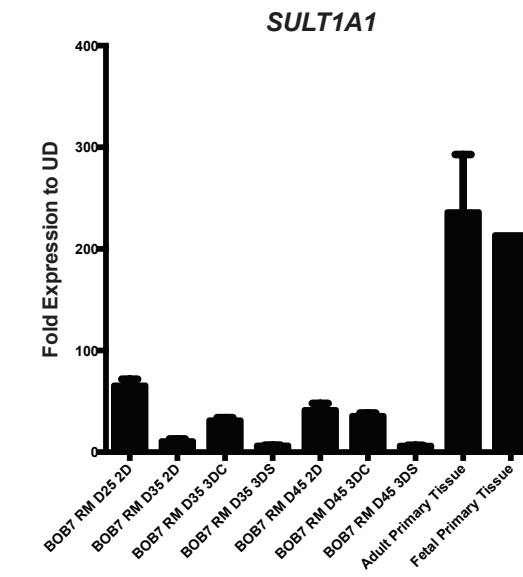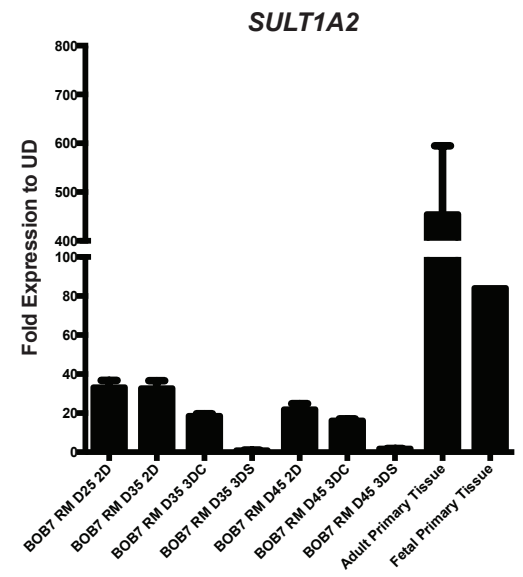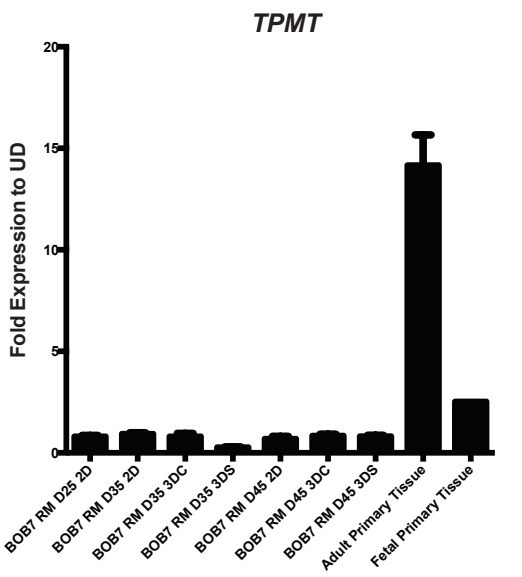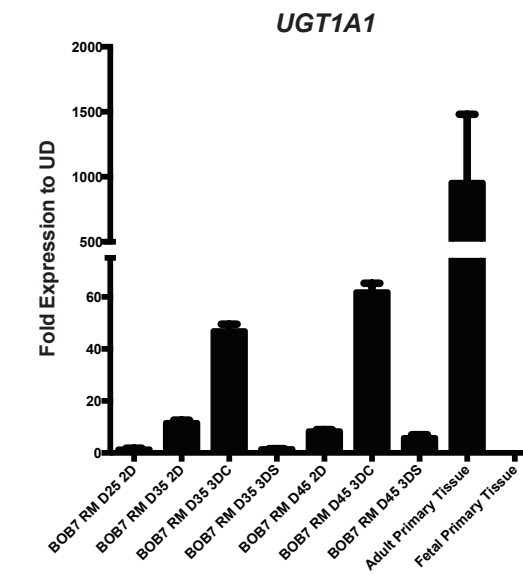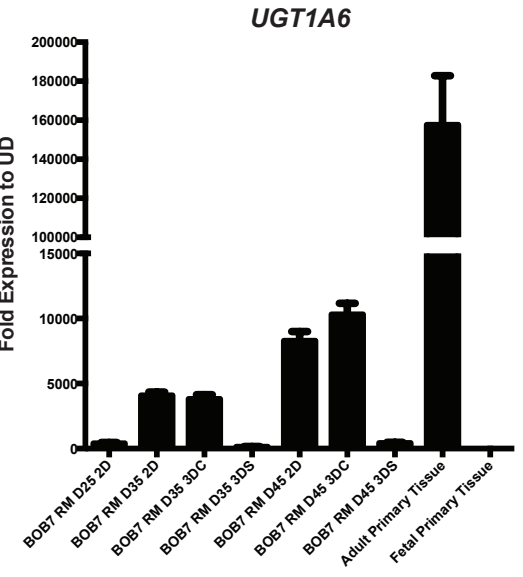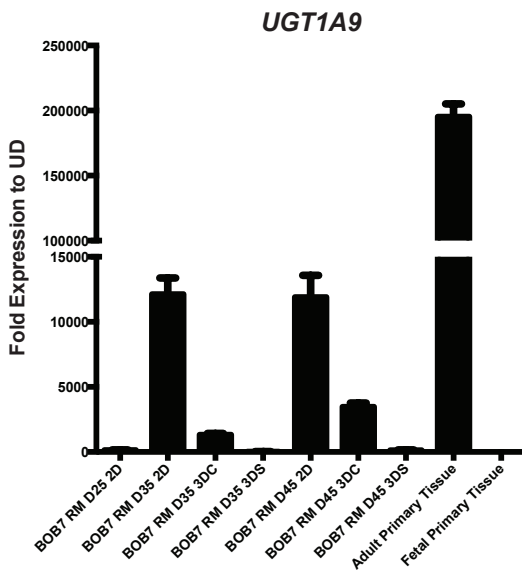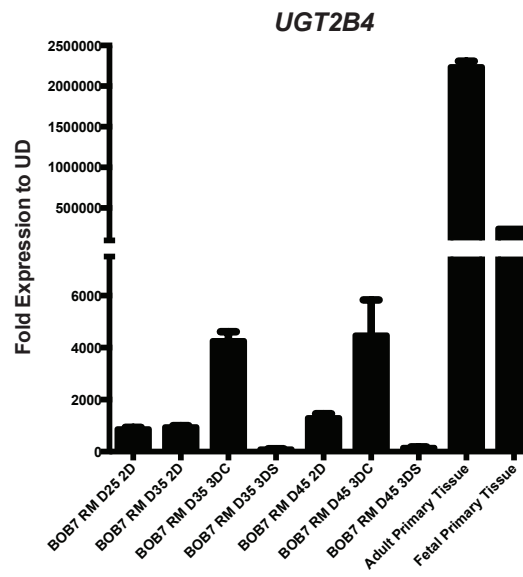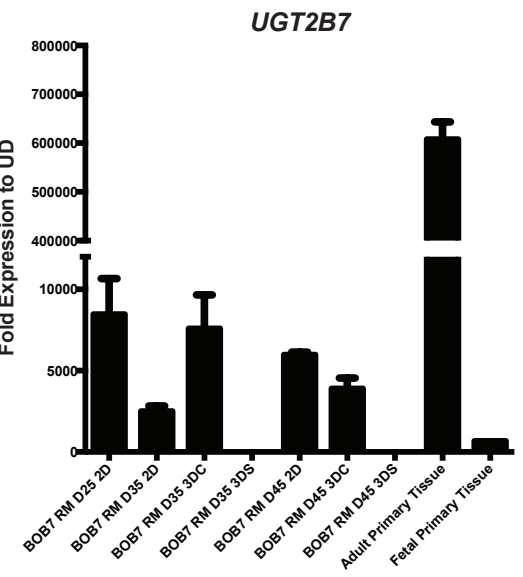

Supplement: Figure S3 — qPCR analysis for BOB7 RM. (a–c) Fold expression to undifferentiated IPSCs; mean ± s.d.; n = 3 biological replicates. (PDF) [file pone.0086372.s003.pdf]

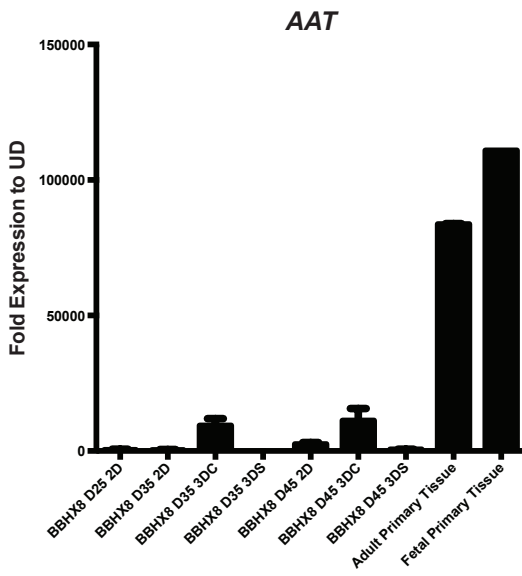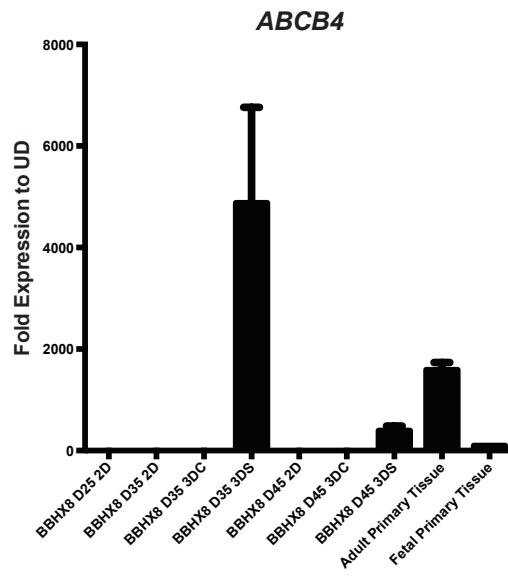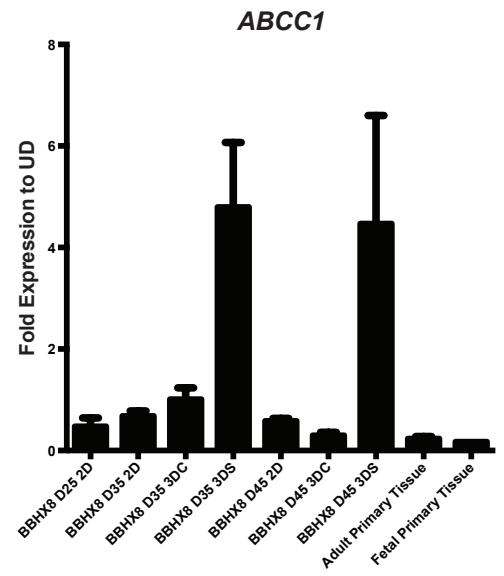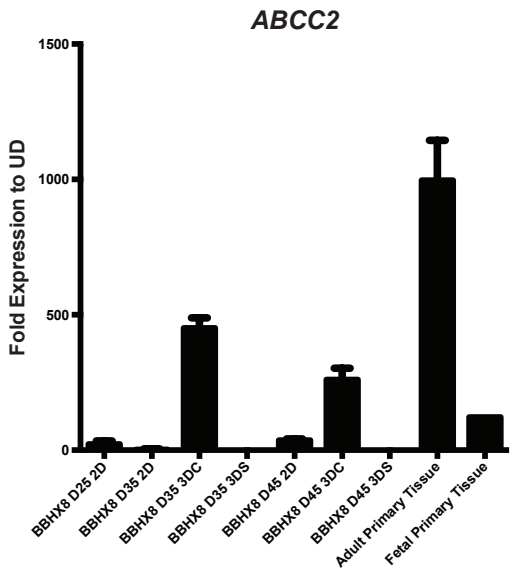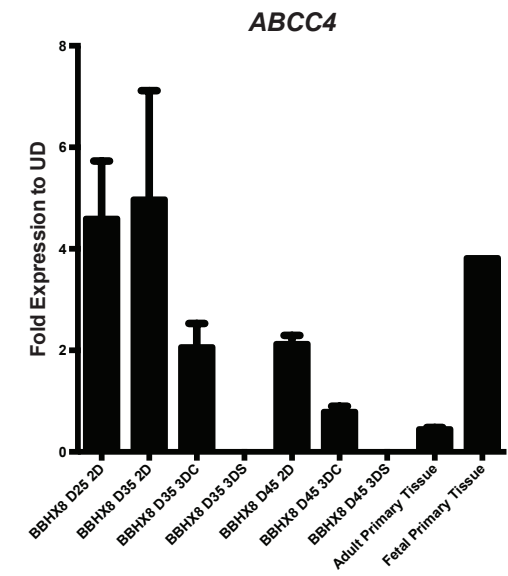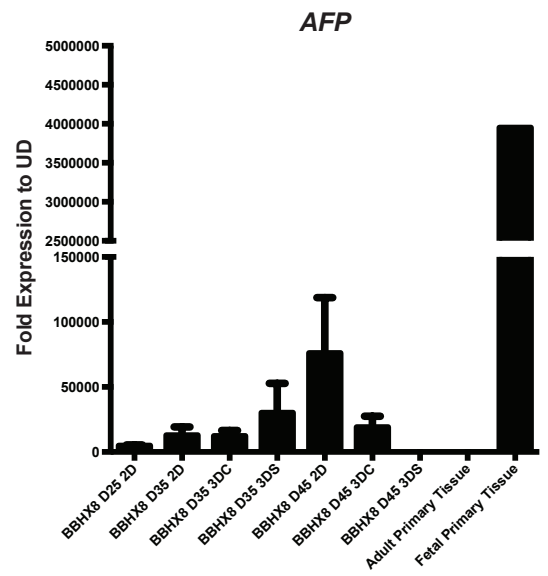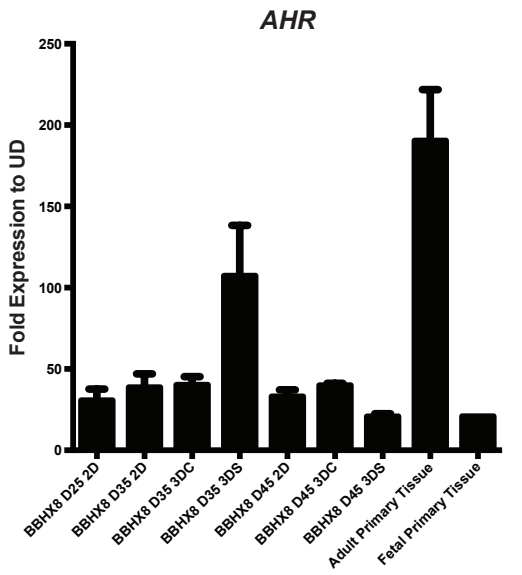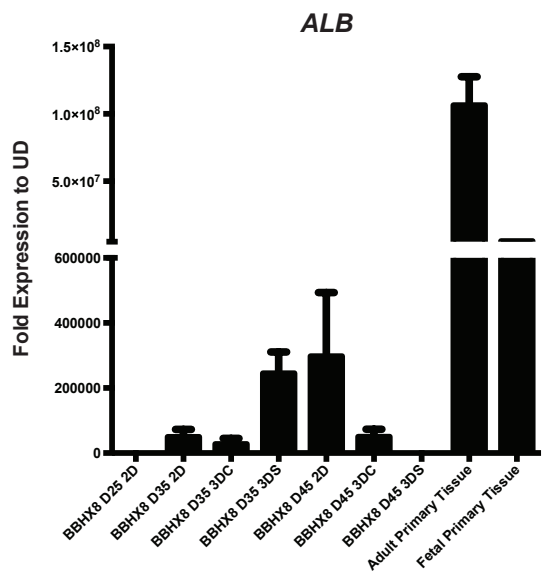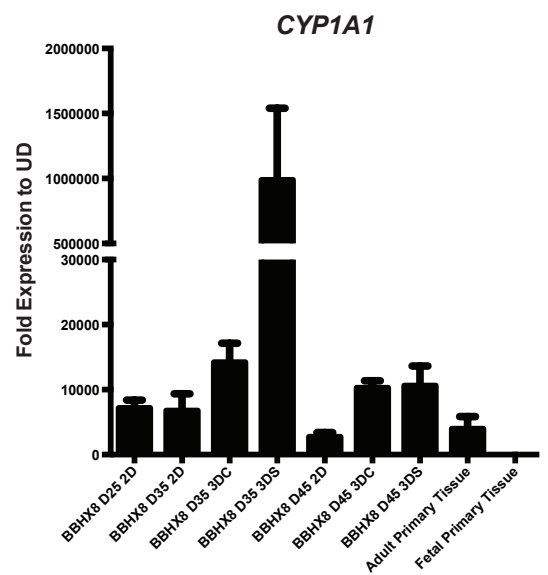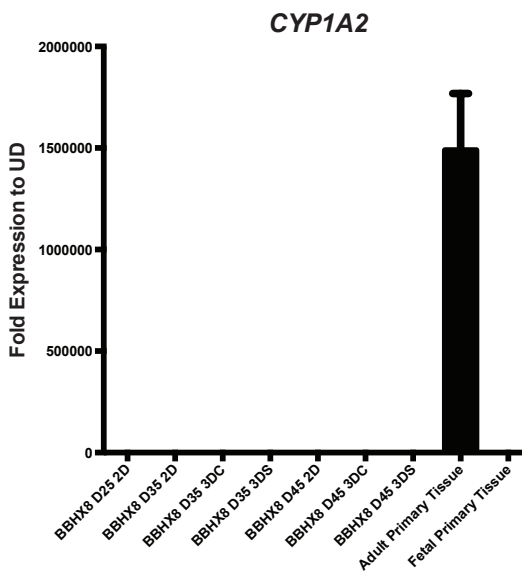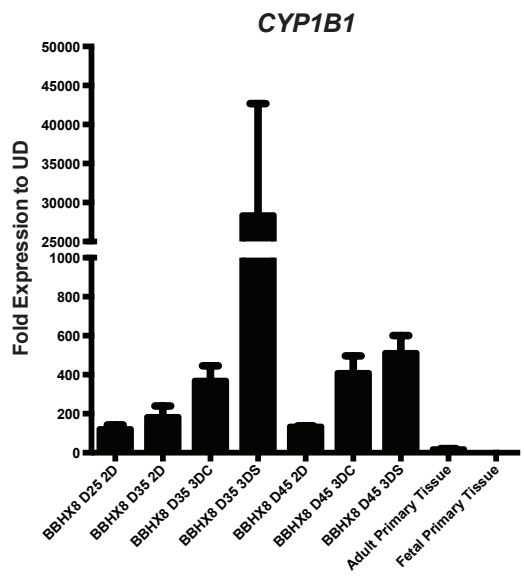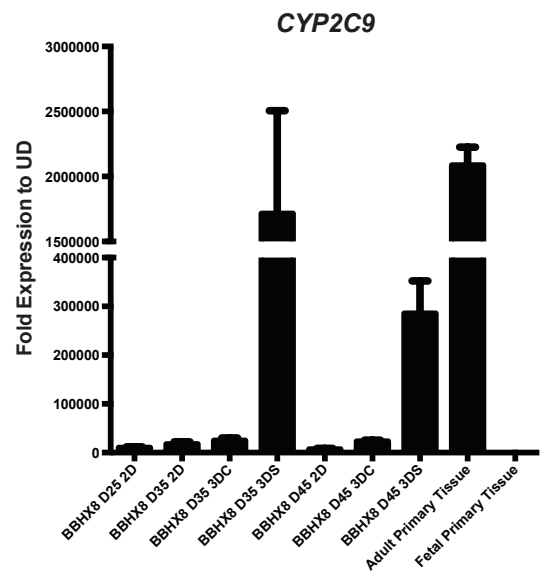

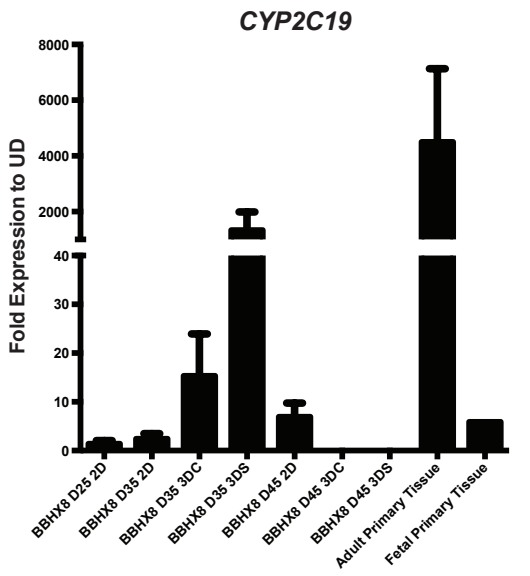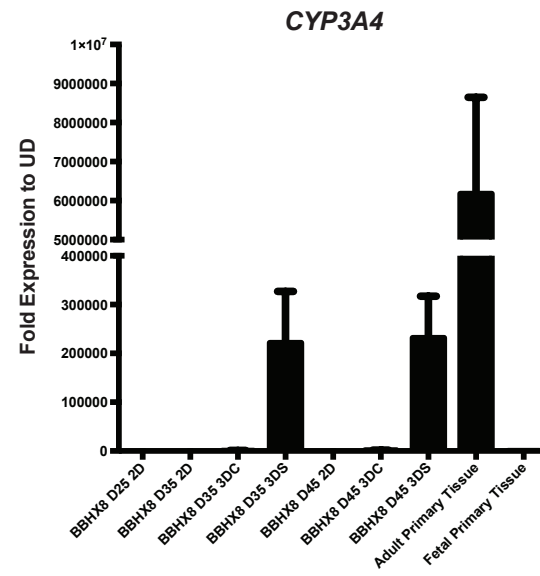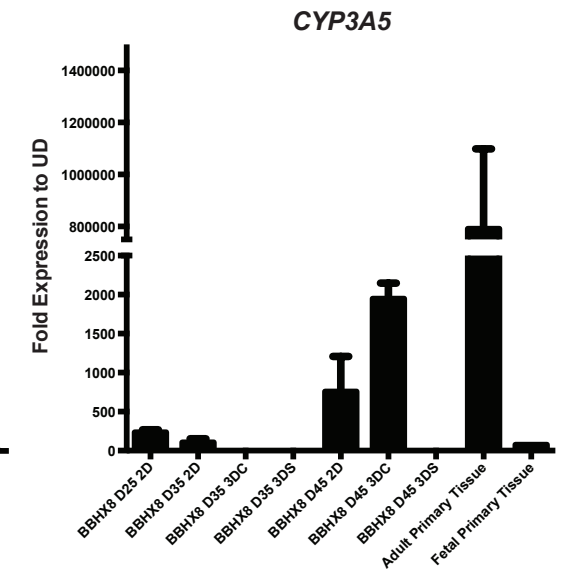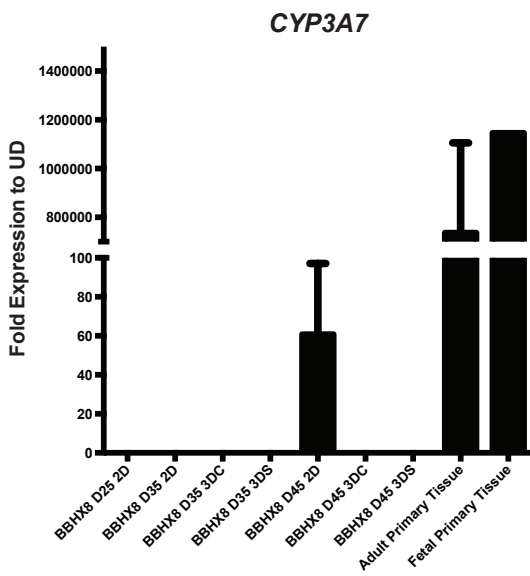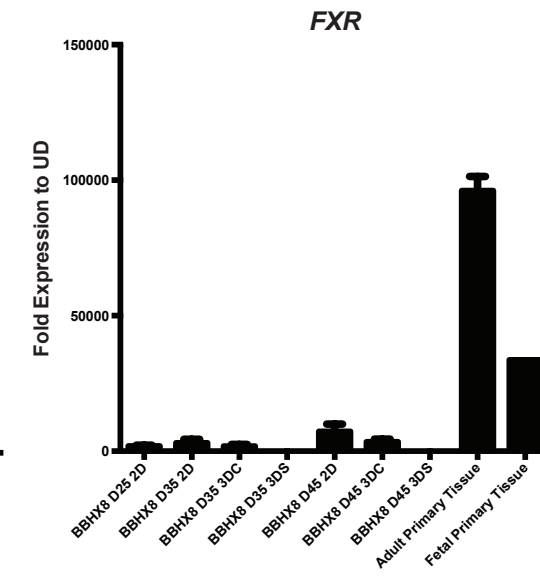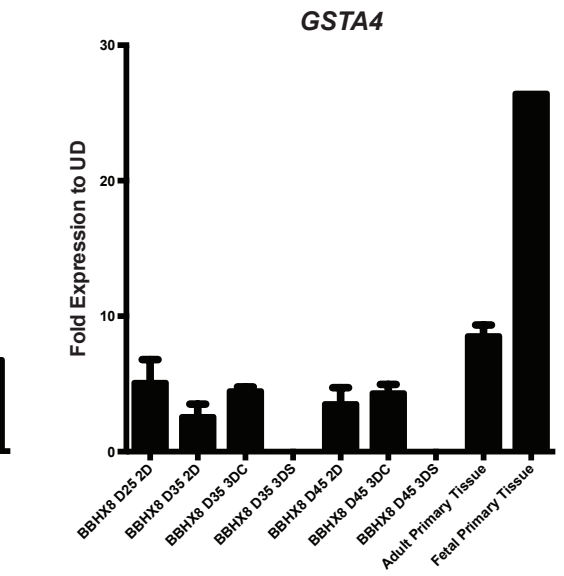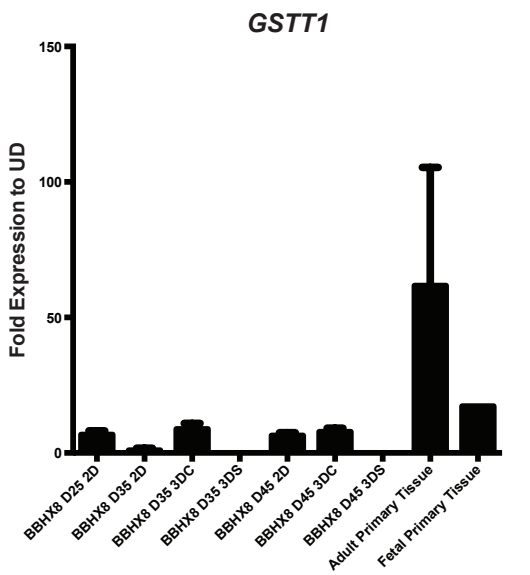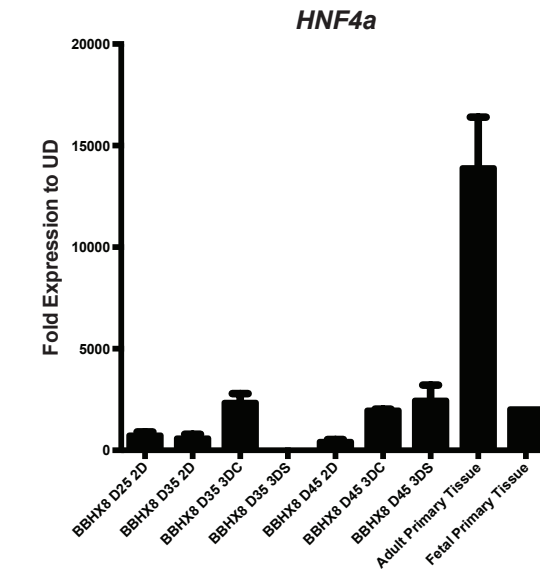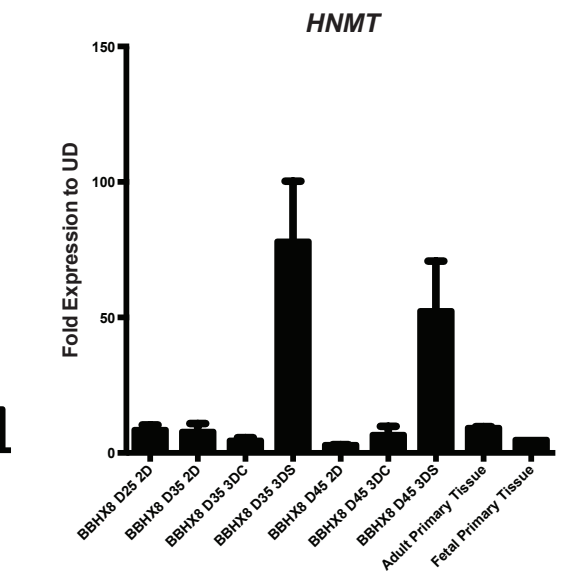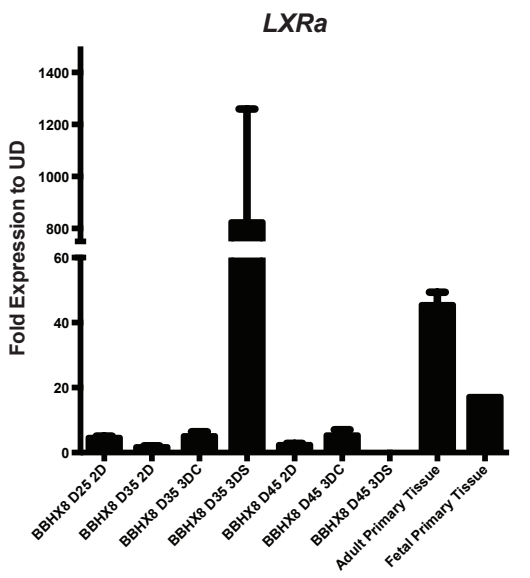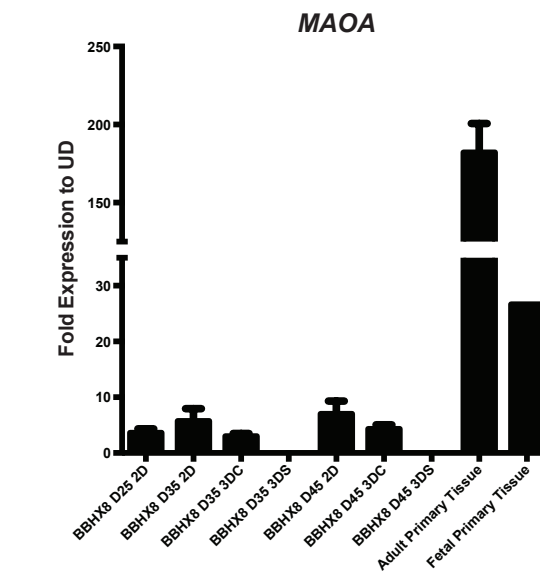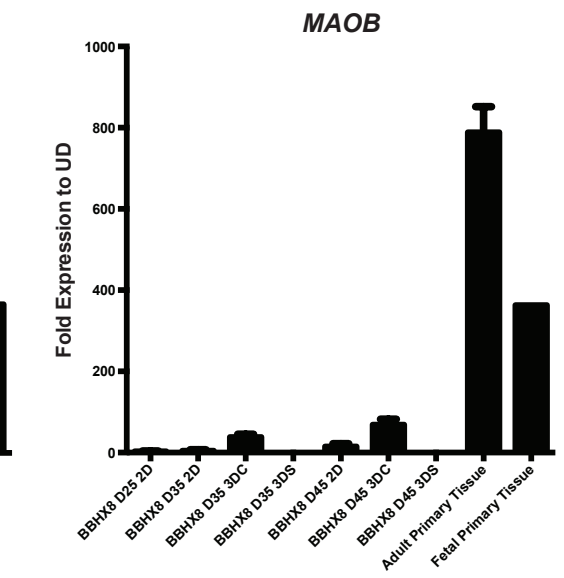

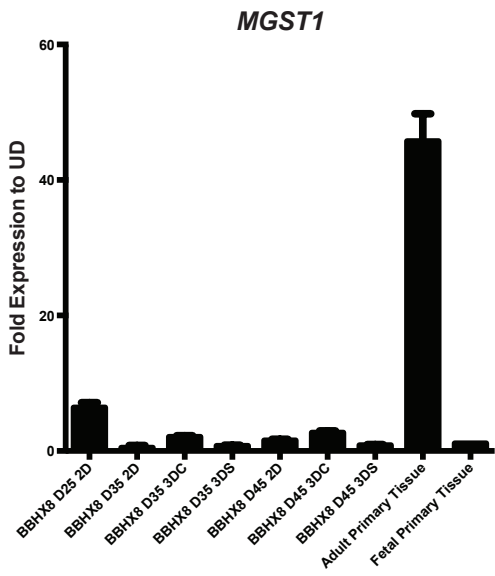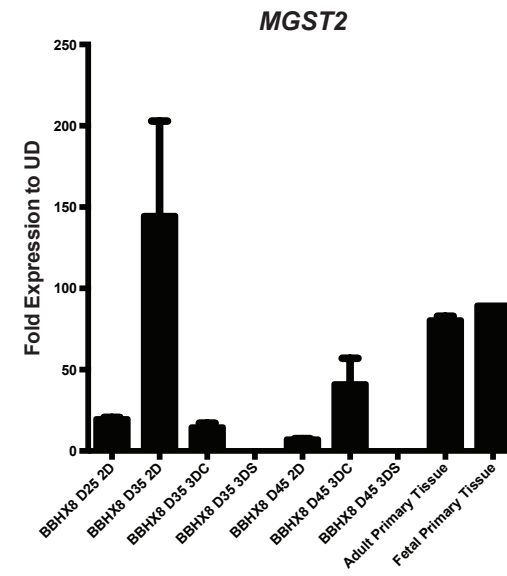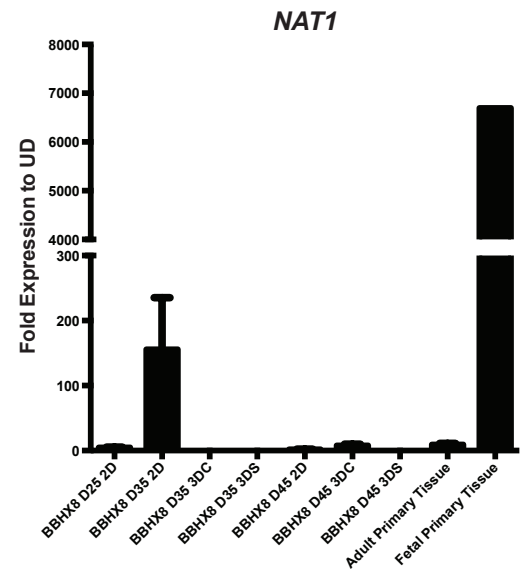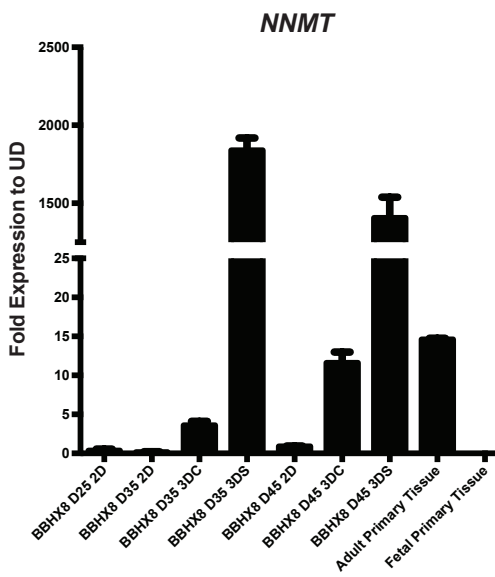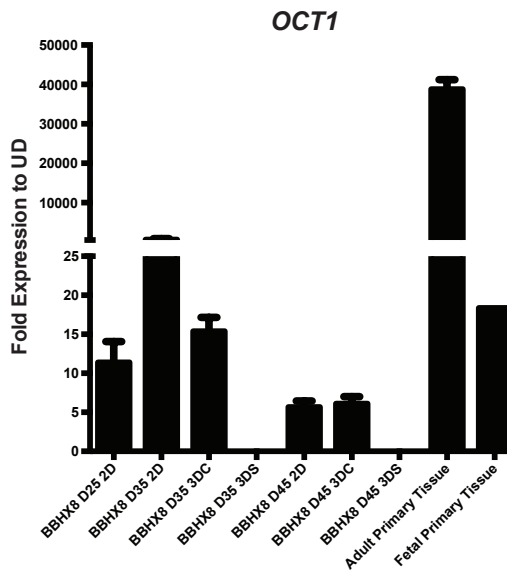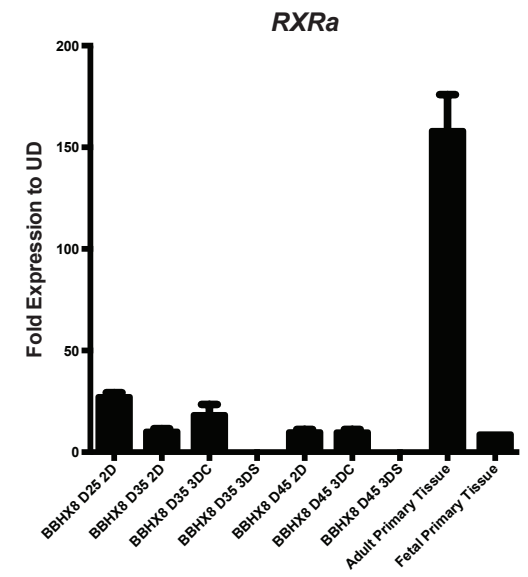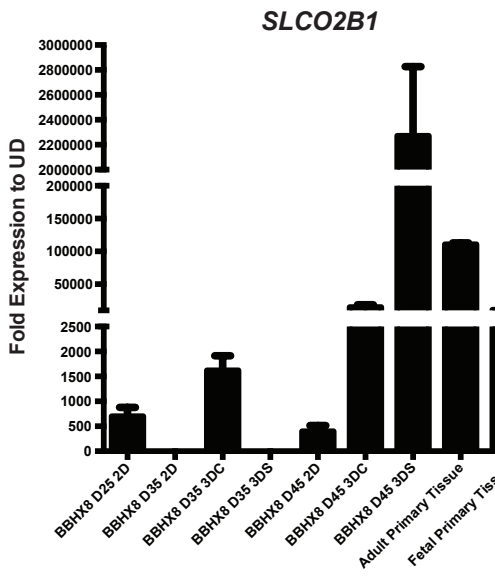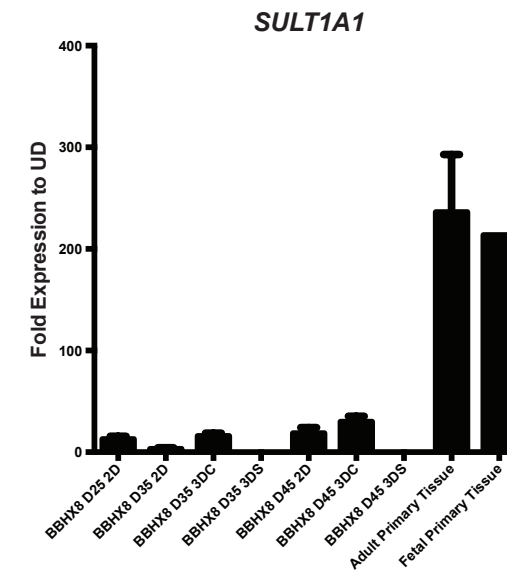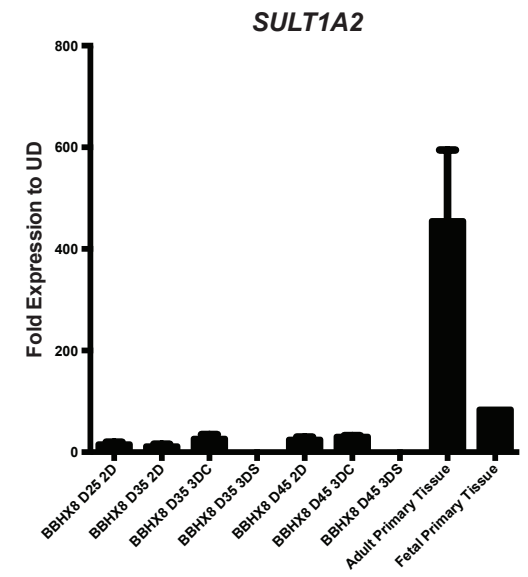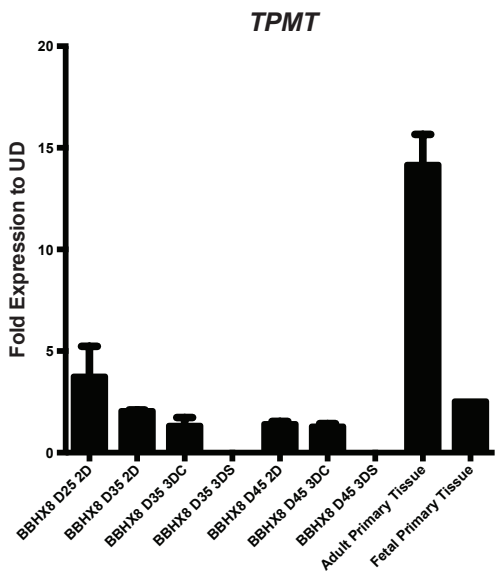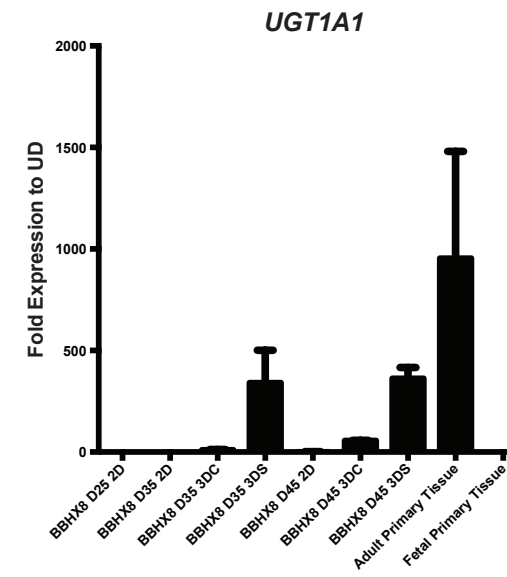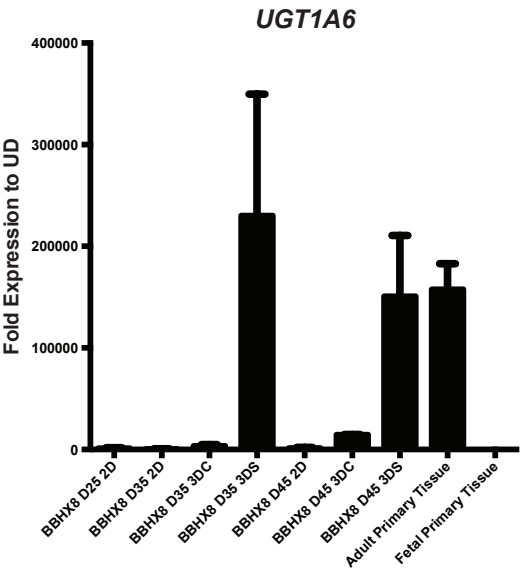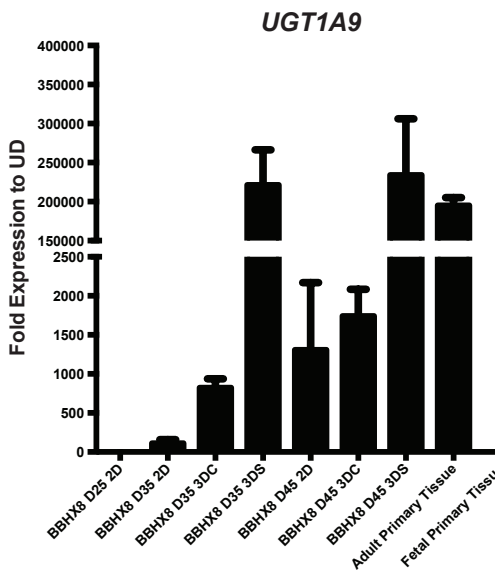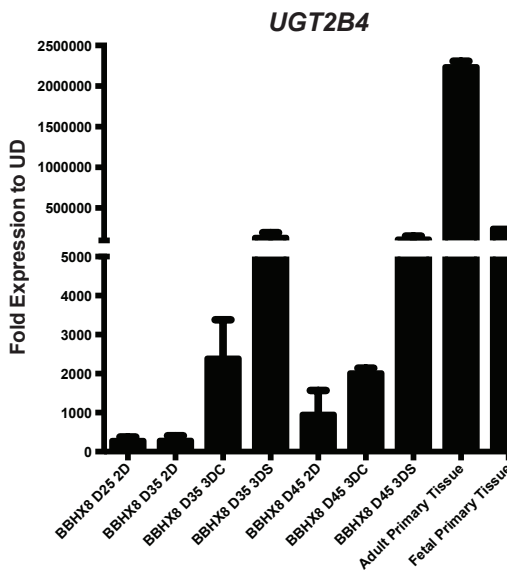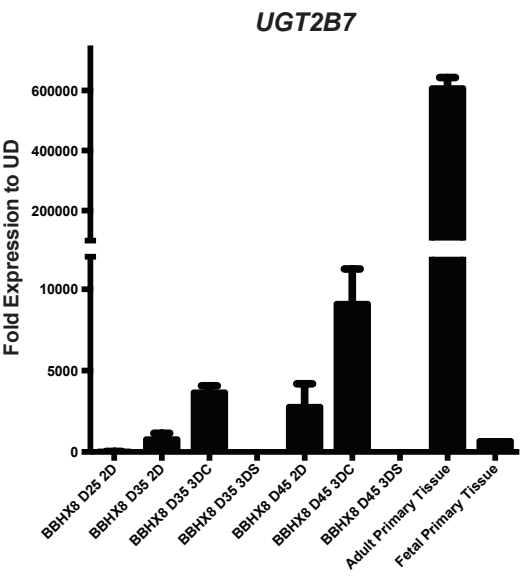

Supplement: Figure S4 — qPCR analysis for BBHX8. (a–c) Fold expression to undifferentiated IPSCs; mean ± s.d.; n = 3 biological replicates. (PDF) [file pone.0086372.s004.pdf]

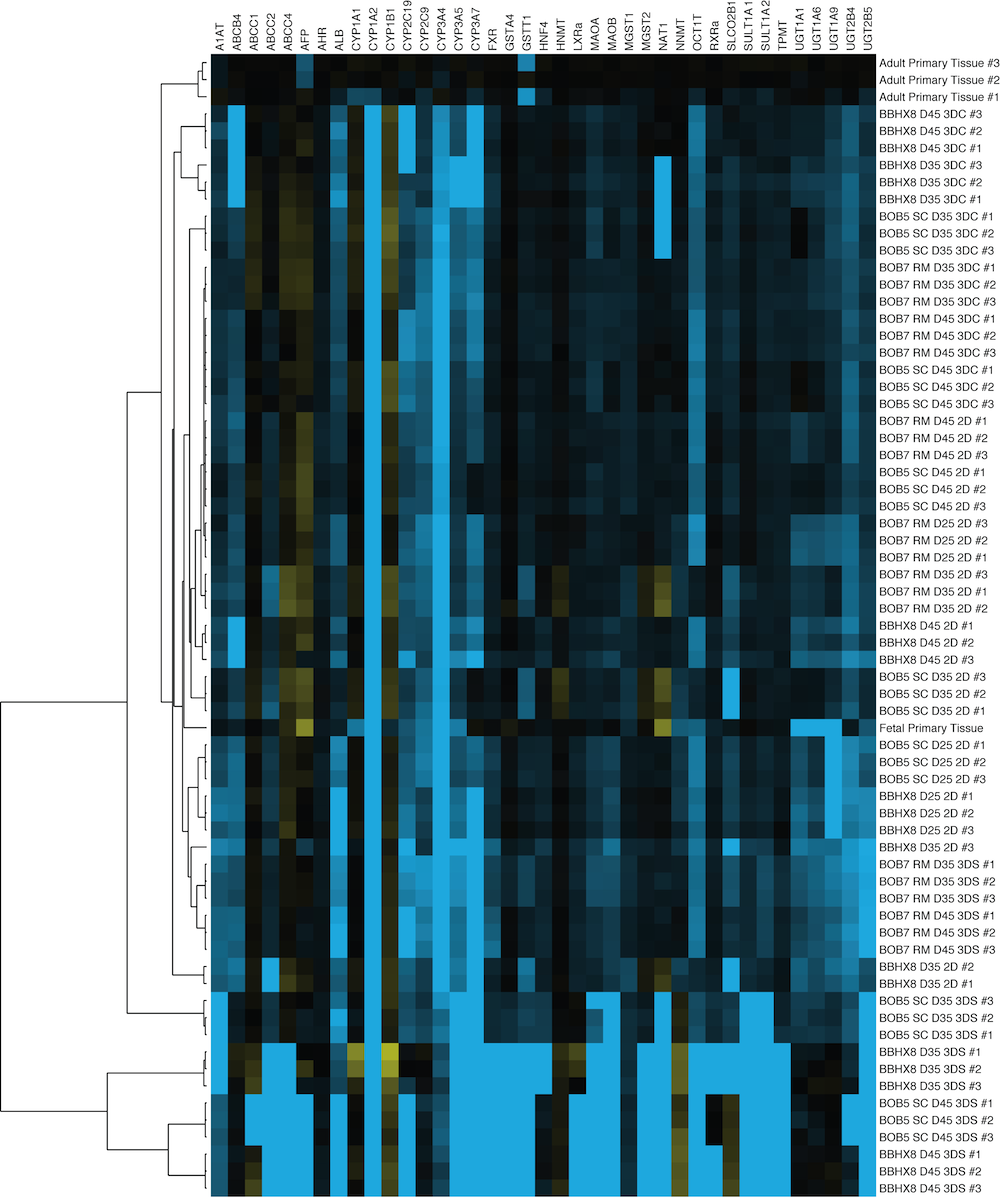

Supplement: Figure S5 — qPCR heatmap. Heatmap of 39 hepatic genes comparing all conditions and lines investigated in this study (fold expression normalized to adult hepatocytes). (TIFF) [file pone.0086372.s005.tiff]

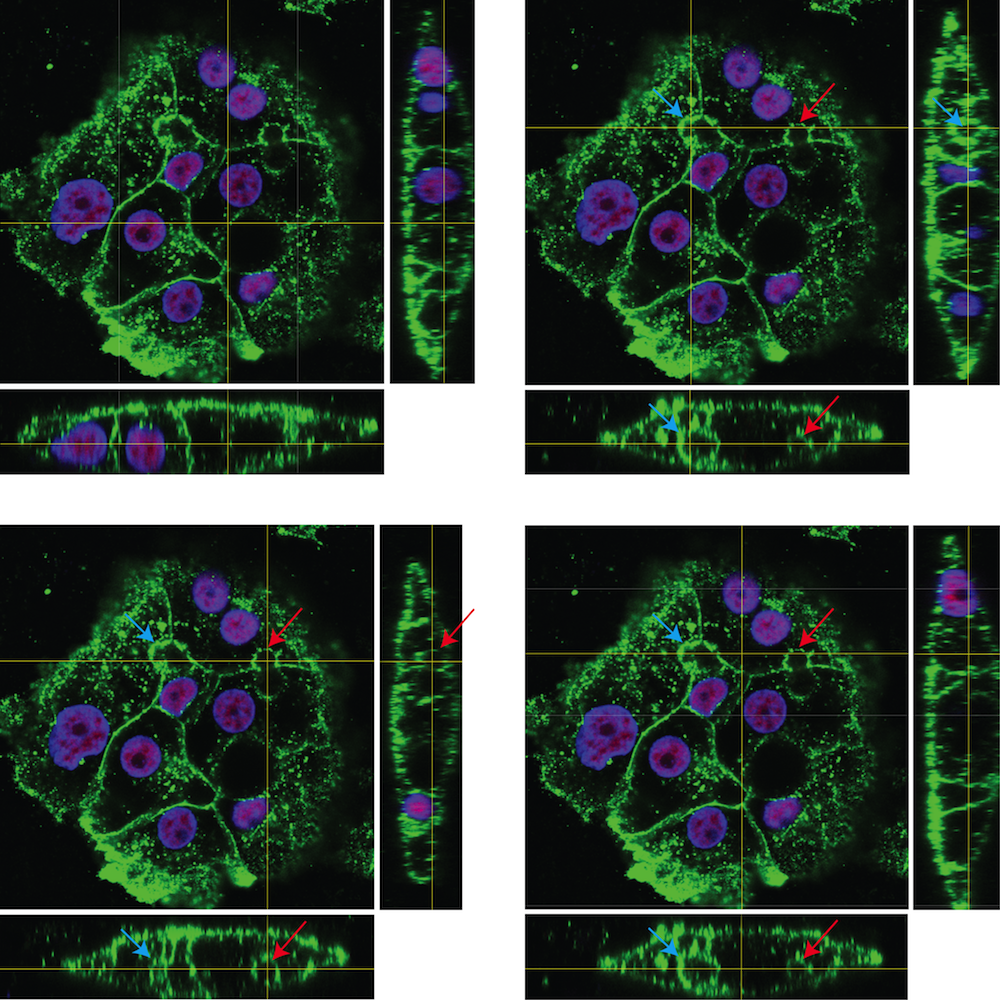

Supplement: Figure S6 — Canalicular structures. Orthogonal analysis of 3D clump cultures demonstrating the presence of canalicular buds (green – ASGPR, Red – HNF4a, blue Hoechst). (TIFF) [file pone.0086372.s006.tiff]

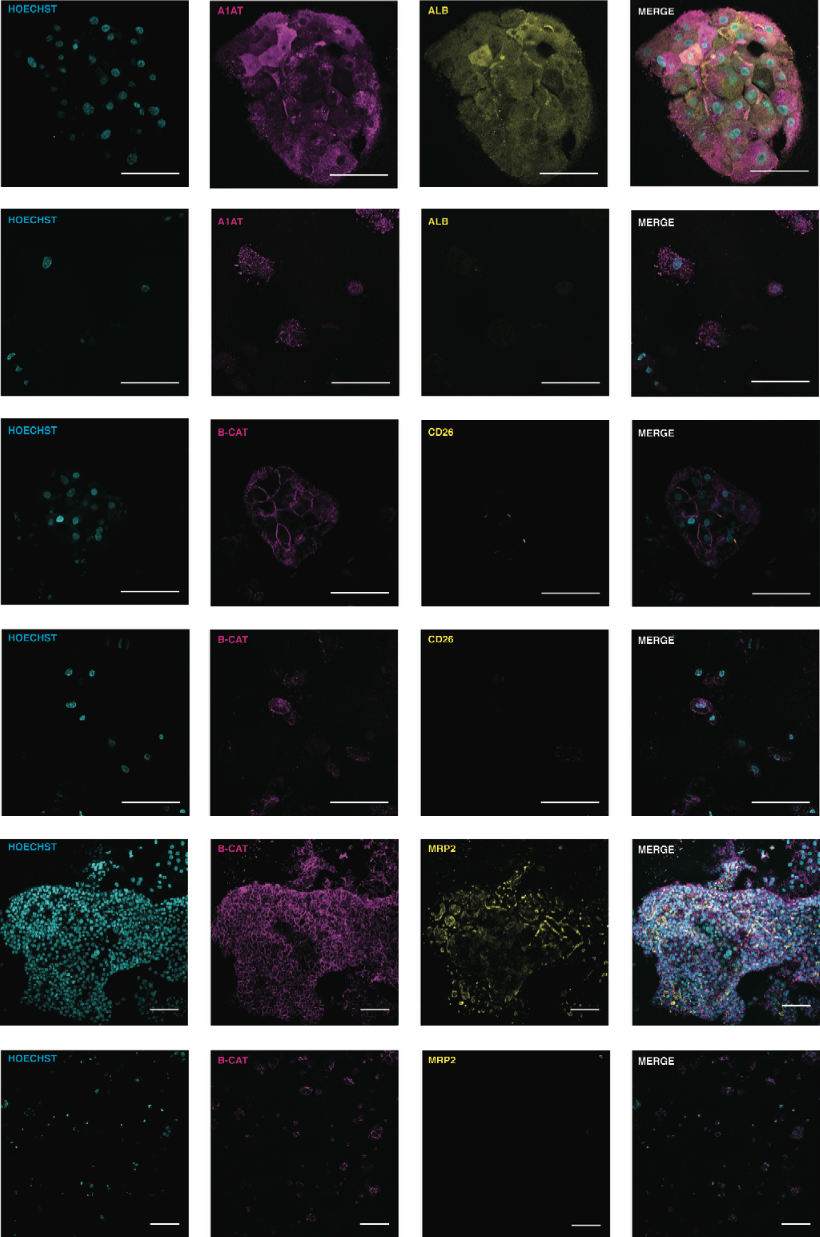

Supplement: Figure S7 — Confocal micrographs. Single channel and merged confocal micrographs of images shown in Figure 1 (scalebar = 100 microns). (TIFF) [file pone.0086372.s007.tiff]

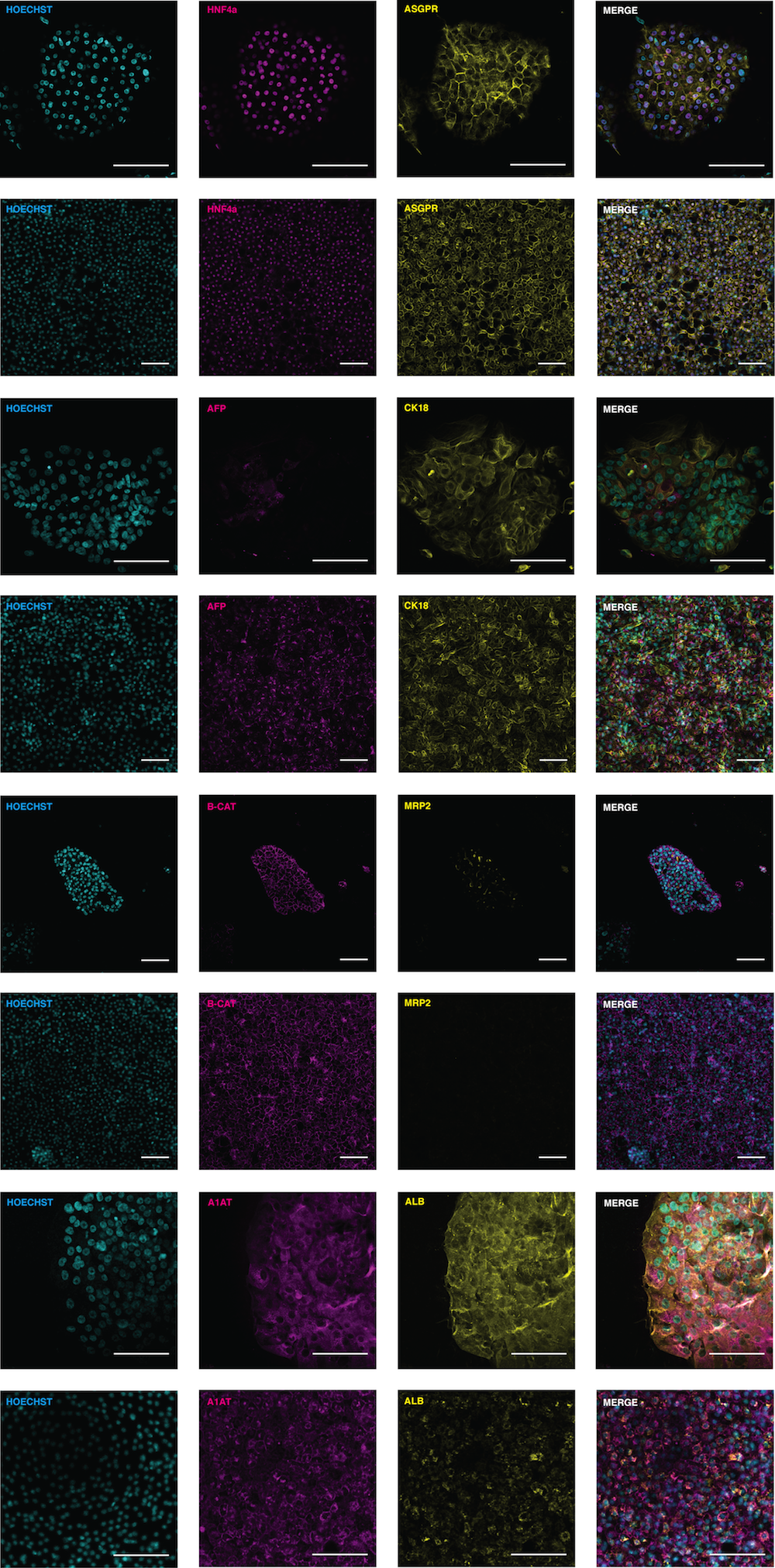

Supplement: Figure S8 — Confocal micrographs. Single channel and merged confocal micrographs of images shown in Figure 2 (scalebar = 100 microns). (TIFF) [file pone.0086372.s008.tiff]

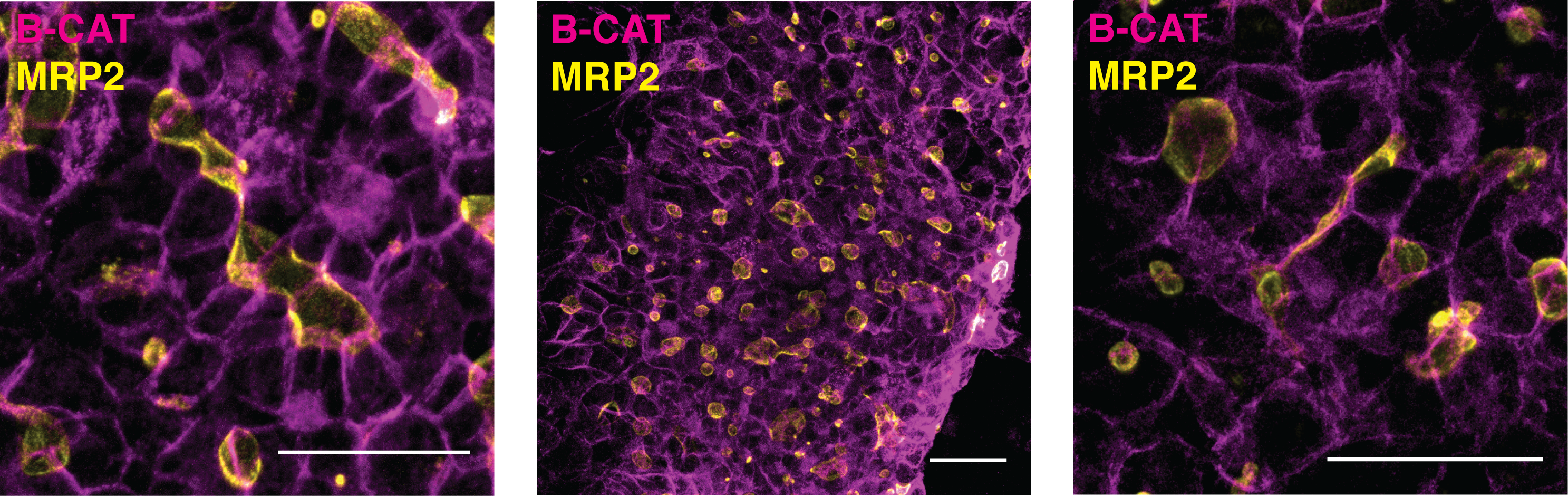

Supplement: Figure S9 — Canalicular structures. Confocal micrographs demonstrating the presence and localization of bile canaliculi and canalicular buds within the 3D clump cultures (scalebar = 100 microns). (TIF) [file pone.0086372.s009.tif]

# CYP3A4 Activity

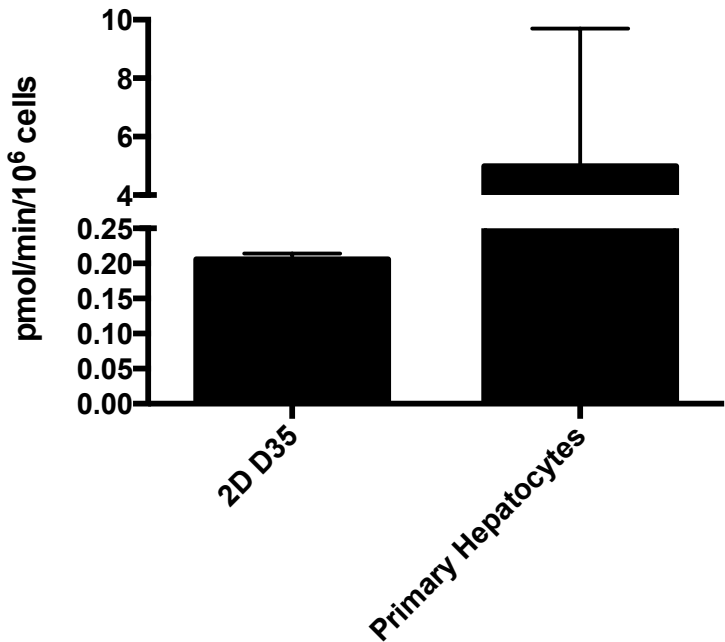

Supplement: Figure S10 — Cytochrome P450 Activity. CYP3A4 activity of the 2D progenitor as assessed by the rate of conversion of Midazolam to 1′-HO-Midazolam using HPLC-MS (n = 35 primary samples; range 10%–200% activity of individual primary samples). (PDF) [file pone.0086372.s010.pdf]
