# Supplementary material for: Maturation of Induced Pluripotent Stem Cell Derived Hepatocytes by 3D-Culture
Source: PLoS One. 2014 Jan 22;9(1):e86372. doi: 10.1371/journal.pone.0086372 (PMC3899231; doi:10.1371/journal.pone.0086372)
Supplement: Table S1 — List of primer sequences used for qPCR analyses. (PDF) [file pone.0086372.s011.pdf]

Gene	Full Name	Accession	Forward
ABCB4	Homo sapiens ATP-binding cassette, sub-family B (MDR/TAP), member 4 (ABCB4), transcript variant A, mRNA	NM_000443.3	CGGGGACAGTGCTTCTCGAT
ABCC1	Homo sapiens ATP-binding cassette, sub-family C (CFTR/MRP), member 1 (ABCC1), mRNA	NM_004996.3	ATCGCTCACCCCTGTTCTCG
ABCC2	Homo sapiens ATP-binding cassette, sub-family C (CFTR/MRP), member 2 (ABCC2), mRNA	NM_000392.3	AGAGTCTTCGTTCCAGACGCA
ABCC4	Homo sapiens ATP-binding cassette, sub-family C (CFTR/MRP), member 4 (ABCC4), transcript variant 1, mRNA	NM_005845.3	TTGCTGCCGCTGACGTTTTT
AHR	Homo sapiens aryl hydrocarbon receptor (AHR), mRNA	NM_001621.4	TGTGCCGAGTCCCATATCCG
CYP2C9	Homo sapiens cytochrome P450, family 2, subfamily C, polypeptide 9 (CYP2C9), mRNA	NM_000771.3	GCCGGCATGGAGCTGTTTTTAT
FXR	Homo sapiens nuclear receptor subfamily 1, group H, member 4 (NR1H4), transcript variant 1, mRNA	NM_001206979.1	GCAAAGAGATGGGAATGTTGGCT
GSTA4	Homo sapiens glutathione S-transferase alpha 4 (GSTA4), mRNA	NM_001512.3	GATGGGTTTTAGCTGCCGCC
GSTT1	Homo sapiens glutathione S-transferase theta 1 (GSTT1), mRNA	NM_000853.2	GTCGGTCGGTCCCCACTATG
HNMT1	Homo sapiens N-myristoyltransferase 1 (NMT1), mRNA	NM_021079.3	ACAATATCCGCCAGGAGCCC
LXRα	Homo sapiens nuclear receptor subfamily 1, group H, member 3 (NR1H3), transcript variant 1, mRNA	NM_005693.3	GTCCCTCCCCTCAGCCTTTC
MAOA	Homo sapiens monoamine oxidase A (MAOA), nuclear gene encoding mitochondrial protein, transcript variant 1, mRNA	NM_000240.3	CGGAGGTGGCATTTCAGGACT
MAOB	Homo sapiens monoamine oxidase B (MAOB), nuclear gene encoding mitochondrial protein, mRNA	NM_000898.4	ACAAATGCGACGTGGTCGTG
MGST1	Homo sapiens microsomal glutathione S-transferase 1 (MGST1), transcript variant 5, mRNA	NM_001260511.1	TCGGCCTCACCACCAAAATTGA
MGST2	Homo sapiens microsomal glutathione S-transferase 2 (MGST2), transcript variant 1, mRNA	NM_002413.4	ATTGTGGATGGCTGGGTGGT
NAT1	Homo sapiens N-acetyltransferase 1 (arylamine N-acetyltransferase) (NAT1), transcript variant 1, mRNA	NM_001160170.1	GGCCCAACAGGCTTTCTACC
NNMT	Homo sapiens nicotinamide N-methyltransferase (NNMT), mRNA	NM_006169.2	GTCTACAATCCCTGGCGGCT
OCT1	Homo sapiens solute carrier family 22 (organic cation transporter), member 1 (SLC22A1), transcript variant 1, mRNA	NM_003057.2	GGTGTGTTCCTCCCTGTGTGA
RXRα	Homo sapiens retinoid X receptor, alpha (RXRA), mRNA	NM_002957.4	GTCGCAGACATGGACACCAA
SLCO2B1	Homo sapiens solute carrier organic anion transporter family, member 2B1 (SLCO2B1), transcript variant 2, mRNA	NM_001145211.2	TCAGGGAGAGCCAGAGGTGA
SULT1A1	Homo sapiens sulfotransferase family, cytosolic, 1A, phenol-preferring, member 1 (SULT1A1), transcript variant 1, mRNA	NM_001055.3	CTCAGAGAACAACCCTGCAT
SULT1A2	Homo sapiens sulfotransferase family, cytosolic, 1A, phenol-preferring, member 2 (SULT1A2), transcript variant 1, mRNA	NM_001054.3	TTCCCCACACAACACCCACA
TPMT	Homo sapiens thiopurine S-methyltransferase (TPMT), mRNA	NM_000367.2	AACCAGCTGTAAGCGAGGCA
UGT1A1	Homo sapiens UDP glucuronosyltransferase 1 family, polypeptide A1 (UGT1A1), mRNA	NM_000463.2	TGATCCCAGTGGATGGCAGC
UGT1A6	Homo sapiens UDP glucuronosyltransferase 1 family, polypeptide A6 (UGT1A6), transcript variant 1, mRNA	NM_001072.3	GGAGCCCTGTGATTTGGAGAGT
UGT1A9	Homo sapiens UDP glucuronosyltransferase 1 family, polypeptide A9 (UGT1A9), mRNA	NM_021027.2	AGGAACATTTATTATGCCACCGTTT
UGT2B4	Homo sapiens UDP glucuronosyltransferase 2 family, polypeptide B4 (UGT2B4), mRNA	NM_021139.2	AAAGGAGCAGCAACTGGAAAACA
UGTB7	Homo sapiens UDP glucuronosyltransferase 2 family, polypeptide B7 (UGT2B7), mRNA	NM_001074.2	TCCCAACAACTCATCCGCTC
AAT	Homo sapiens serpin peptidase inhibitor, clade A (alpha-1 antiproteinase, antitrypsin), member 1, mRNA	NM_000295.4	CCATTGCTGAAGACCTTAGTGATG
AFP	Homo sapiens alpha-fetoprotein (AFP), mRNA	NM_001134.2	AGAACCTGTCACAAGCTGTG
ALB	Homo sapiens albumin (ALB), mRNA	NM_000477.5	CCTTTGGCACAATGAAGTGGGTAACC
CYP1A1	Homo sapiens cytochrome P450, family 1, subfamily A, polypeptide 1 (CYP1A1), mRNA	NM_000499.3	AAACAGGGCCACATAGATGC
CYP1A2	Homo sapiens cytochrome P450, family 1, subfamily A, polypeptide 2 (CYP1A2), mRNA	NM_000761.3	AGGTCAACCATGACCCAGAG
CYP1B1	Homo sapiens cytochrome P450, family 1, subfamily B, polypeptide 1 (CYP1B1), mRNA	NM_000104.3	CACCAAGGCTGAGACAGTGA
CYP2C19	Homo sapiens cytochrome P450, family 2, subfamily C, polypeptide 19 (CYP2C19), mRNA	NM_000769.1	AACTCCCTCCTGGCCCCACT
CYP3A4	Homo sapiens cytochrome P450, family 3, subfamily A, polypeptide 4 (CYP3A4), transcript variant 1, mRNA	NM_017460.5	TGTGCCTGAGAACACCAGAG
CYP3A5	Homo sapiens cytochrome P450, family 3, subfamily A, polypeptide 5 (CYP3A5), mRNA	NM_000777.2	CCCCTTTGTGGAGAGCACTA
CYP3A7	Homo sapiens cytochrome P450, family 3, subfamily A, polypeptide 7 (CYP3A7), mRNA	NM_000765	ATTCCAAGCTATGTTCTTCATCAT
HNF4	Homo sapiens hepatocyte nuclear factor 4, alpha (HNF4A), transcript variant 1, mRNA	NM_178849.2	CATGGCCAAGATTGACAACCT
PBDG	Homo sapiens hydroxymethylbilane synthase (HMBS), transcript variant 1, mRNA	NM_000190.3	GGAGCCATGTCTGGTAACGG
RPLP0	Homo sapiens ribosomal protein, large, P0 (RPLP0), transcript variant 1, mRNA	NM_001002.3	GGCGTCCTCGTGGAAGTGAC
HDAC	Homo sapiens histone deacetylase 2 (HDAC2), transcript variant 1, mRNA	NM_001527.3	AGTCAAGGAGGCGGCAAAA
GAPDH	Homo sapiens glyceraldehyde-3-phosphate dehydrogenase (GAPDH), transcript variant 1, mRNA	NM_002046.4	GAGTCAACGGATTTGGTCGT
