# Supplementary material for: Maturation of Induced Pluripotent Stem Cell Derived Hepatocytes by 3D-Culture
Source: PLoS One. 2014 Jan 22;9(1):e86372. doi: 10.1371/journal.pone.0086372 (PMC3899231; doi:10.1371/journal.pone.0086372)
Supplement: Table S5 — Significance of BBHX8 qPCR analyses by Welch’s T-test. (PDF) [file pone.0086372.s015.pdf]

Gene	BBHX8 D35 2D vs. BBHX8 D25 2D	BBHX8 D35 3DC vs. BBHX8 D25 2D	BBHX8 D35 3DC vs. BBHX8 D35 2D	BBHX8 D35 3DS vs. BBHX8 D25 2D	BBHX8 D35 3DS vs. BBHX8 D35 2D	BBHX8 D35 3DS vs. BBHX8 D35 3DC	BBHX8 D45 2D vs. BBHX8 D25 2D	BBHX8 D45 2D vs. BBHX8 D35 2D	BBHX8 D45 2D vs. BBHX8 D35 3DC	BBHX8 D45 2D vs. BBHX8 D35 3DS	BBHX8 D45 3DC vs. BBHX8 D25 2D	BBHX8 D45 3DC vs. BBHX8 D35 2D	BBHX8 D45 3DC vs. BBHX8 D35 3DC	BBHX8 D45 3DC vs. BBHX8 D35 3DS
A1AT	n.s.	*	*	G.N.E.	G.N.E.	G.N.E.	*	*	n.s.	G.N.E.	n.s.	n.s.	n.s.	G.N.E.
ABCB4	n.s.	G.N.E.	G.N.E.	n.s.	n.s.	G.N.E.	G.N.E.	G.N.E.	G.N.E.	G.N.E.	G.N.E.	G.N.E.	G.N.E.	G.N.E.
ABCC1	n.s.	n.s.	n.s.	*	*	n.s.	n.s.	n.s.	n.s.	*	n.s.	*	*	*
ABCC2	G.N.E.	**	G.N.E.	G.N.E.	G.N.E.	G.N.E.	n.s.	G.N.E.	**	G.N.E.	*	G.N.E.	*	G.N.E.
ABCC4	n.s.	n.s.	n.s.	G.N.E.	G.N.E.	G.N.E.	n.s.	n.s.	n.s.	G.N.E.	*	n.s.	**	G.N.E.
AFP	n.s.	n.s.	n.s.	n.s.	n.s.	n.s.	n.s.	n.s.	n.s.	n.s.	n.s.	n.s.	n.s.	n.s.
AHR	n.s.	n.s.	n.s.	n.s.	n.s.	n.s.	n.s.	n.s.	n.s.	n.s.	n.s.	n.s.	***	n.s.
ALB	n.s.	n.s.	n.s.	*	n.s.	*	n.s.	n.s.	n.s.	n.s.	n.s.	n.s.	n.s.	n.s.
CYP1A1	n.s.	n.s.	n.s.	n.s.	n.s.	n.s.	*	n.s.	*	n.s.	n.s.	n.s.	**	n.s.
CYP1A2	G.N.E.	G.N.E.	G.N.E.	G.N.E.	G.N.E.	G.N.E.	G.N.E.	G.N.E.	G.N.E.	G.N.E.	G.N.E.	G.N.E.	G.N.E.	G.N.E.
CYP1B1	n.s.	*	n.s.	n.s.	n.s.	n.s.	n.s.	n.s.	*	n.s.	*	n.s.	*	n.s.
CYP2C19	G.N.E.	G.N.E.	G.N.E.	n.s.	G.N.E.	G.N.E.	n.s.	G.N.E.	G.N.E.	n.s.	G.N.E.	G.N.E.	G.N.E.	G.N.E.
CYP2C9	n.s.	n.s.	n.s.	n.s.	n.s.	n.s.	n.s.	n.s.	*	n.s.	**	n.s.	**	n.s.
CYP3A4	G.N.E.	*	G.N.E.	n.s.	G.N.E.	n.s.	n.s.	G.N.E.	**	n.s.	*	G.N.E.	**	n.s.
CYP3A5	*	G.N.E.	G.N.E.	G.N.E.	G.N.E.	G.N.E.	n.s.	n.s.	G.N.E.	G.N.E.	**	**	**	G.N.E.
CYP3A7	G.N.E.	G.N.E.	G.N.E.	G.N.E.	G.N.E.	G.N.E.	G.N.E.	G.N.E.	G.N.E.	G.N.E.	G.N.E.	G.N.E.	G.N.E.	G.N.E.
FXR	n.s.	n.s.	n.s.	G.N.E.	G.N.E.	G.N.E.	n.s.	n.s.	n.s.	G.N.E.	n.s.	n.s.	*	G.N.E.
GSTA4	n.s.	n.s.	n.s.	G.N.E.	G.N.E.	G.N.E.	n.s.	n.s.	n.s.	G.N.E.	n.s.	n.s.	*	G.N.E.
GSTT1	*	n.s.	*	G.N.E.	G.N.E.	G.N.E.	n.s.	**	n.s.	G.N.E.	n.s.	**	*	G.N.E.
HNF4	n.s.	*	*	G.N.E.	G.N.E.	G.N.E.	n.s.	n.s.	*	G.N.E.	*	*	***	G.N.E.
HNMT	n.s.	n.s.	n.s.	*	*	*	*	n.s.	n.s.	*	n.s.	n.s.	n.s.	*
LXRa	**	n.s.	n.s.	n.s.	n.s.	n.s.	*	n.s.	n.s.	n.s.	n.s.	n.s.	*	n.s.
MAOA	n.s.	n.s.	n.s.	G.N.E.	G.N.E.	G.N.E.	n.s.	n.s.	n.s.	G.N.E.	n.s.	n.s.	*	G.N.E.
MAOB	n.s.	*	*	G.N.E.	G.N.E.	G.N.E.	n.s.	n.s.	*	G.N.E.	*	*	*	G.N.E.
MGST1	**	*	**	**	n.s.	**	*	*	*	**	*	**	**	*
MGST2	n.s.	n.s.	n.s.	G.N.E.	G.N.E.	G.N.E.	**	n.s.	n.s.	G.N.E.	n.s.	n.s.	n.s.	G.N.E.
NAT1	n.s.	G.N.E.	G.N.E.	G.N.E.	G.N.E.	G.N.E.	**	n.s.	G.N.E.	G.N.E.	n.s.	n.s.	*	G.N.E.
NNMT	n.s.	*	*	***	***	***	n.s.	***	*	***	**	**	**	***
OCT1T	n.s.	n.s.	n.s.	G.N.E.	G.N.E.	G.N.E.	n.s.	n.s.	*	G.N.E.	n.s.	n.s.	*	G.N.E.
RXRa	**	n.s.	n.s.	G.N.E.	G.N.E.	G.N.E.	**	n.s.	n.s.	G.N.E.	**	n.s.	*	G.N.E.
SLCO2B1	G.N.E.	*	G.N.E.	G.N.E.	G.N.E.	G.N.E.	n.s.	G.N.E.	*	G.N.E.	*	G.N.E.	*	G.N.E.
SULT1A1	*	n.s.	*	G.N.E.	G.N.E.	G.N.E.	n.s.	n.s.	n.s.	G.N.E.	n.s.	*	*	G.N.E.
SULT1A2	n.s.	n.s.	n.s.	G.N.E.	G.N.E.	G.N.E.	n.s.	n.s.	n.s.	G.N.E.	*	*	**	G.N.E.
TPMT	n.s.	n.s.	n.s.	G.N.E.	G.N.E.	G.N.E.	n.s.	*	n.s.	G.N.E.	n.s.	*	**	G.N.E.
UGT1A1	n.s.	*	*	n.s.	n.s.	n.s.	n.s.	n.s.	*	n.s.	***	***	***	n.s.
UGT1A6	n.s.	n.s.	n.s.	n.s.	n.s.	n.s.	n.s.	n.s.	n.s.	n.s.	***	****	****	n.s.
UGT1A9	G.N.E.	G.N.E.	*	G.N.E.	*	*	G.N.E.	n.s.	n.s.	*	G.N.E.	*	*	*
UGT2B4	n.s.	n.s.	n.s.	n.s.	n.s.	n.s.	n.s.	n.s.	n.s.	n.s.	***	***	**	n.s.
UGT2B7	G.N.E.	**	G.N.E.	G.N.E.	G.N.E.	G.N.E.	n.s.	G.N.E.	n.s.	G.N.E.	*	G.N.E.	*	G.N.E.

Gene	BBHX8 D45 3DC vs. BBHX8 D45 2D	BBHX8 D45 3DS vs. BBHX8 D25 2D	BBHX8 D45 3DS vs. BBHX8 D35 2D	BBHX8 D45 3DS vs. BBHX8 D35 3DC	BBHX8 D45 3DS vs. BBHX8 D35 3DS	BBHX8 D45 3DS vs. BBHX8 D45 2D	BBHX8 D45 3DS vs. BBHX8 D45 3DC	Primary Adult vs. BBHX8 D25 2D	Primary Adult vs. BBHX8 D35 2D	Primary Adult vs. BBHX8 D35 3DC	Primary Adult vs. BBHX8 D35 3DS	Primary Adult vs. BBHX8 D45 2D	Primary Adult vs. BBHX8 D45 3DC	Primary Adult vs. BBHX8 D45 3DS
A1AT	n.s.	n.s.	n.s.	*	G.N.E.	*	n.s.	****	****	****	G.N.E.	****	****	****
ABCB4	G.N.E.	*	*	G.N.E.	n.s.	G.N.E.	G.N.E.	*	*	G.N.E.	n.s.	G.N.E.	G.N.E.	n.s.
ABCC1	*	n.s.	n.s.	n.s.	n.s.	n.s.	n.s.	n.s.	*	*	*	*	n.s.	n.s.
ABCC2	*	G.N.E.	G.N.E.	G.N.E.	G.N.E.	G.N.E.	G.N.E.	*	G.N.E.	n.s.	G.N.E.	*	n.s.	G.N.E.
ABCC4	**	G.N.E.	G.N.E.	G.N.E.	G.N.E.	G.N.E.	G.N.E.	*	n.s.	*	G.N.E.	**	*	G.N.E.
AFP	n.s.	G.N.E.	G.N.E.	G.N.E.	G.N.E.	G.N.E.	G.N.E.	n.s.	n.s.	n.s.	n.s.	n.s.	n.s.	G.N.E.
AHR	n.s.	n.s.	n.s.	*	n.s.	n.s.	***	n.s.	n.s.	n.s.	n.s.	n.s.	n.s.	n.s.
ALB	n.s.	G.N.E.	G.N.E.	G.N.E.	G.N.E.	G.N.E.	G.N.E.	n.s.	n.s.	n.s.	n.s.	n.s.	n.s.	G.N.E.
CYP1A1	**	n.s.	n.s.	n.s.	n.s.	n.s.	n.s.	n.s.	n.s.	*	n.s.	n.s.	n.s.	n.s.
CYP1A2	G.N.E.	G.N.E.	G.N.E.	G.N.E.	G.N.E.	G.N.E.	G.N.E.	G.N.E.	G.N.E.	G.N.E.	G.N.E.	G.N.E.	G.N.E.	G.N.E.
CYP1B1	*	*	*	n.s.	n.s.	*	n.s.	*	*	*	n.s.	****	*	*
CYP2C19	G.N.E.	G.N.E.	G.N.E.	G.N.E.	G.N.E.	G.N.E.	G.N.E.	n.s.	G.N.E.	G.N.E.	n.s.	n.s.	G.N.E.	G.N.E.
CYP2C9	**	*	*	*	n.s.	*	*	*	*	*	n.s.	*	*	*
CYP3A4	*	n.s.	G.N.E.	n.s.	n.s.	n.s.	n.s.	**	G.N.E.	**	**	**	**	**
CYP3A5	n.s.	G.N.E.	G.N.E.	G.N.E.	G.N.E.	G.N.E.	G.N.E.	n.s.	n.s.	G.N.E.	G.N.E.	n.s.	n.s.	G.N.E.
CYP3A7	G.N.E.	G.N.E.	G.N.E.	G.N.E.	G.N.E.	G.N.E.	G.N.E.	G.N.E.	G.N.E.	G.N.E.	G.N.E.	n.s.	G.N.E.	G.N.E.
FXR	n.s.	G.N.E.	G.N.E.	G.N.E.	G.N.E.	G.N.E.	G.N.E.	*	*	*	G.N.E.	*	*	G.N.E.
GSTA4	n.s.	G.N.E.	G.N.E.	G.N.E.	G.N.E.	G.N.E.	G.N.E.	n.s.	*	n.s.	G.N.E.	n.s.	n.s.	G.N.E.
GSTT1	n.s.	G.N.E.	G.N.E.	G.N.E.	G.N.E.	G.N.E.	G.N.E.	n.s.	n.s.	n.s.	G.N.E.	n.s.	n.s.	G.N.E.
HNF4	***	n.s.	n.s.	n.s.	G.N.E.	n.s.	n.s.	n.s.	n.s.	n.s.	G.N.E.	n.s.	n.s.	n.s.
HNMT	n.s.	n.s.	n.s.	n.s.	n.s.	n.s.	n.s.	n.s.	n.s.	*	*	**	n.s.	n.s.
LXRa	n.s.	G.N.E.	G.N.E.	G.N.E.	G.N.E.	G.N.E.	G.N.E.	*	*	*	n.s.	*	*	G.N.E.
MAOA	n.s.	G.N.E.	G.N.E.	G.N.E.	G.N.E.	G.N.E.	G.N.E.	*	*	*	G.N.E.	*	*	G.N.E.
MAOB	*	G.N.E.	G.N.E.	G.N.E.	G.N.E.	G.N.E.	G.N.E.	*	*	*	G.N.E.	*	*	G.N.E.
MGST1	*	**	n.s.	**	n.s.	*	*	*	*	*	*	*	*	*
MGST2	n.s.	G.N.E.	G.N.E.	G.N.E.	G.N.E.	G.N.E.	G.N.E.	**	n.s.	**	G.N.E.	**	n.s.	G.N.E.
NAT1	*	G.N.E.	G.N.E.	G.N.E.	G.N.E.	G.N.E.	G.N.E.	n.s.	n.s.	G.N.E.	G.N.E.	n.s.	n.s.	G.N.E.
NNMT	**	**	**	**	*	**	**	****	***	***	***	***	n.s.	**
OCT1T	n.s.	G.N.E.	G.N.E.	G.N.E.	G.N.E.	G.N.E.	G.N.E.	*	*	*	G.N.E.	*	*	G.N.E.
RXRa	n.s.	G.N.E.	G.N.E.	G.N.E.	G.N.E.	G.N.E.	G.N.E.	*	*	*	G.N.E.	*	*	G.N.E.
SLCO2B1	*	*	G.N.E.	*	G.N.E.	*	*	**	G.N.E.	**	G.N.E.	**	***	*
SULT1A1	n.s.	G.N.E.	G.N.E.	G.N.E.	G.N.E.	G.N.E.	G.N.E.	n.s.	n.s.	n.s.	G.N.E.	n.s.	n.s.	G.N.E.
SULT1A2	n.s.	G.N.E.	G.N.E.	G.N.E.	G.N.E.	G.N.E.	G.N.E.	n.s.	n.s.	n.s.	G.N.E.	n.s.	n.s.	G.N.E.
TPMT	n.s.	G.N.E.	G.N.E.	G.N.E.	G.N.E.	G.N.E.	G.N.E.	n.s.	n.s.	*	G.N.E.	*	*	G.N.E.
UGT1A1	****	*	*	*	n.s.	*	*	n.s.	n.s.	n.s.	n.s.	n.s.	n.s.	n.s.
UGT1A6	**	n.s.	n.s.	n.s.	n.s.	n.s.	n.s.	*	*	*	n.s.	*	*	n.s.
UGT1A9	n.s.	G.N.E.	*	*	n.s.	*	*	G.N.E.	*	*	n.s.	*	*	n.s.
UGT2B4	n.s.	n.s.	n.s.	n.s.	n.s.	n.s.	n.s.	**	**	**	**	**	**	**
UGT2B7	*	G.N.E.	G.N.E.	G.N.E.	G.N.E.	G.N.E.	G.N.E.	*	G.N.E.	*	G.N.E.	*	*	G.N.E.

Key	
G.N.E	Gene not expressed in one or both
n.s.	P > 0.05
*	P ≤ 0.05
**	P < 0.01
***	P < 0.001
****	P < 0.0001
