# Supplementary material for: Maturation of Induced Pluripotent Stem Cell Derived Hepatocytes by 3D-Culture
Source: PLoS One. 2014 Jan 22;9(1):e86372. doi: 10.1371/journal.pone.0086372 (PMC3899231; doi:10.1371/journal.pone.0086372)
Supplement: Table S6 — Significance of BOB5 SC qPCR analyses by Welch’s T-test. (PDF) [file pone.0086372.s016.pdf]

Gene	BOB5 SC D35 2D vs. BOB5 SC D25 2D	BOB5 SC D35 3DC vs. BOB5 SC D25 2D	BOB5 SC D35 3DC vs. BOB5 SC D35 2D	BOB5 SC D35 3DS vs. BOB5 SC D25 2D	BOB5 SC D35 3DS vs. BOB5 SC D35 2D	BOB5 SC D35 3DS vs. BOB5 SC D35 3DC	BOB5 SC D45 2D vs. BOB5 SC D25 2D	BOB5 SC D45 2D vs. BOB5 SC D35 2D	BOB5 SC D45 2D vs. BOB5 SC D35 3DC	BOB5 SC D45 2D vs. BOB5 SC D35 3DS	BOB5 SC D45 3DC vs. BOB5 SC D25 2D	BOB5 SC D45 3DC vs. BOB5 SC D35 2D	BOB5 SC D45 3DC vs. BOB5 SC D35 3DC	BOB5 SC D45 3DC vs. BOB5 SC D35 3DS	BOB5 SC D45 3DC vs. BOB5 SC D45 2D	BOB5 SC D45 3DS vs. BOB5 SC D25 2D	BOB5 SC D45 3DS vs. BOB5 SC D35 2D	BOB5 SC D45 3DS vs. BOB5 SC D35 3DC	BOB5 SC D45 3DS vs. BOB5 SC D35 3DS	BOB5 SC D45 3DS vs. BOB5 SC D45 2D	BOB5 SC D45 3DS vs. BOB5 SC D45 3DC	Primary Adult vs. BOB5 SC D25 2D	Primary Adult vs. BOB5 SC D35 2D	Primary Adult vs. BOB5 SC D35 3DC	Primary Adult vs. BOB5 SC D35 3DS	Primary Adult vs. BOB5 SC D45 2D	Primary Adult vs. BOB5 SC D45 3DC	Primary Adult vs. BOB5 SC D45 3DS
A1AT	*	n.s.	n.s.	G.N.E.	G.N.E.	G.N.E.	*	n.s.	n.s.	G.N.E.	n.s.	n.s.	*	G.N.E.	n.s.	*	*	n.s.	G.N.E.	*	*	****	***	***	G.N.E.	***	****	****
ABCB4	*	**	n.s.	**	**	**	**	n.s.	n.s.	**	*	n.s.	*	**	**	*	*	*	*	*	*	*	*	*	*	*	*	n.s.
ABCC1	**	**	n.s.	n.s.	*	**	n.s.	*	**	*	n.s.	*	*	n.s.	n.s.	G.N.E.	G.N.E.	G.N.E.	G.N.E.	G.N.E.	G.N.E.	n.s.	**	**	n.s.	*	n.s.	G.N.E.
ABCC2	*	**	**	n.s.	**	**	*	**	**	*	*	*	*	*	*	G.N.E.	G.N.E.	G.N.E.	G.N.E.	G.N.E.	G.N.E.	*	*	n.s.	*	*	n.s.	G.N.E.
ABCC4	n.s.	*	n.s.	**	*	**	n.s.	n.s.	**	*	n.s.	*	*	n.s.	n.s.	G.N.E.	G.N.E.	G.N.E.	G.N.E.	G.N.E.	G.N.E.	***	*	**	*	*	*	G.N.E.
AFP	*	**	*	*	*	**	**	n.s.	**	**	n.s.	*	n.s.	n.s.	***	G.N.E.	G.N.E.	G.N.E.	G.N.E.	G.N.E.	G.N.E.	n.s.	*	**	*	**	n.s.	G.N.E.
AHR	**	n.s.	*	n.s.	**	**	n.s.	**	**	n.s.	n.s.	**	***	n.s.	n.s.	n.s.	n.s.	n.s.	n.s.	n.s.	n.s.	n.s.	n.s.	n.s.	n.s.	n.s.	n.s.	n.s.
ALB	*	n.s.	*	n.s.	*	n.s.	**	n.s.	**	**	*	*	*	*	**	G.N.E.	G.N.E.	G.N.E.	G.N.E.	G.N.E.	G.N.E.	n.s.	n.s.	n.s.	n.s.	n.s.	n.s.	G.N.E.
CYP1A1	**	*	n.s.	***	**	*	**	**	*	**	***	n.s.	***	****	***	n.s.	**	*	*	n.s.	***	n.s.	*	*	n.s.	n.s.	n.s.	n.s.
CYP1A2	G.N.E.	G.N.E.	G.N.E.	G.N.E.	G.N.E.	G.N.E.	G.N.E.	G.N.E.	G.N.E.	G.N.E.	G.N.E.	G.N.E.	G.N.E.	G.N.E.	G.N.E.	G.N.E.	G.N.E.	G.N.E.	G.N.E.	G.N.E.	G.N.E.	G.N.E.	G.N.E.	G.N.E.	G.N.E.	G.N.E.	G.N.E.	G.N.E.
CYP1B1	**	*	*	n.s.	*	*	*	*	*	n.s.	**	**	**	***	**	n.s.	n.s.	*	n.s.	n.s.	**	**	**	*	n.s.	**	**	*
CYP2C19	*	n.s.	n.s.	n.s.	*	n.s.	n.s.	n.s.	n.s.	n.s.	*	*	*	*	*	G.N.E.	G.N.E.	G.N.E.	G.N.E.	G.N.E.	G.N.E.	n.s.	n.s.	n.s.	n.s.	n.s.	n.s.	G.N.E.
CYP2C9	*	*	n.s.	n.s.	n.s.	n.s.	**	**	**	n.s.	**	n.s.	**	n.s.	*	n.s.	n.s.	n.s.	n.s.	n.s.	n.s.	*	*	*	*	*	*	*
CYP3A4	*	*	n.s.	n.s.	n.s.	n.s.	***	**	n.s.	n.s.	n.s.	*	*	n.s.	**	*	*	*	n.s.	*	*	**	**	**	**	**	**	**
CYP3A5	*	n.s.	*	G.N.E.	G.N.E.	G.N.E.	*	*	*	G.N.E.	***	n.s.	***	G.N.E.	*	G.N.E.	G.N.E.	G.N.E.	G.N.E.	G.N.E.	G.N.E.	n.s.	n.s.	n.s.	G.N.E.	n.s.	n.s.	G.N.E.
CYP3A7	*	n.s.	*	G.N.E.	G.N.E.	G.N.E.	**	n.s.	**	G.N.E.	n.s.	*	*	G.N.E.	**	G.N.E.	G.N.E.	G.N.E.	G.N.E.	G.N.E.	G.N.E.	n.s.	n.s.	n.s.	G.N.E.	n.s.	n.s.	G.N.E.
FXR	**	*	*	**	*	n.s.	****	n.s.	*	**	**	n.s.	**	*	n.s.	G.N.E.	G.N.E.	G.N.E.	G.N.E.	G.N.E.	G.N.E.	*	*	*	*	*	*	G.N.E.
GSTA4	n.s.	*	n.s.	**	*	**	*	n.s.	n.s.	*	*	n.s.	**	**	n.s.	G.N.E.	G.N.E.	G.N.E.	G.N.E.	G.N.E.	G.N.E.	*	n.s.	n.s.	*	n.s.	n.s.	G.N.E.
GSTT1	*	**	**	*	*	**	**	**	n.s.	**	*	*	**	*	n.s.	G.N.E.	G.N.E.	G.N.E.	G.N.E.	G.N.E.	G.N.E.	n.s.	n.s.	n.s.	n.s.	n.s.	n.s.	G.N.E.
HNF4	**	**	*	**	**	**	*	**	n.s.	*	**	*	**	**	n.s.	*	*	n.s.	*	n.s.	n.s.	n.s.	n.s.	n.s.	n.s.	n.s.	n.s.	n.s.
HNMT	*	n.s.	*	n.s.	*	n.s.	n.s.	*	n.s.	*	n.s.	*	***	n.s.	n.s.	**	*	**	**	**	**	**	*	**	*	**	**	**
LXRa	*	*	*	*	*	*	n.s.	n.s.	n.s.	*	n.s.	n.s.	*	*	n.s.	G.N.E.	G.N.E.	G.N.E.	G.N.E.	G.N.E.	G.N.E.	*	*	*	n.s.	*	*	G.N.E.
MAOA	**	n.s.	**	**	**	*	*	*	*	*	*	**	***	***	*	G.N.E.	G.N.E.	G.N.E.	G.N.E.	G.N.E.	G.N.E.	*	*	*	*	*	*	G.N.E.
MAOB	*	**	**	G.N.E.	G.N.E.	G.N.E.	**	n.s.	**	G.N.E.	**	**	**	G.N.E.	**	G.N.E.	G.N.E.	G.N.E.	G.N.E.	G.N.E.	G.N.E.	*	*	*	G.N.E.	*	*	G.N.E.
MGST1	**	**	*	***	n.s.	n.s.	***	*	n.s.	n.s.	**	****	***	n.s.	n.s.	***	**	n.s.	n.s.	n.s.	n.s.	*	*	*	*	*	*	*
MGST2	**	*	**	n.s.	**	*	n.s.	**	*	n.s.	***	**	***	***	**	G.N.E.	G.N.E.	G.N.E.	G.N.E.	G.N.E.	G.N.E.	**	**	**	**	**	*	G.N.E.
NAT1	*	G.N.E.	G.N.E.	G.N.E.	G.N.E.	G.N.E.	n.s.	*	G.N.E.	G.N.E.	n.s.	*	*	G.N.E.	n.s.	G.N.E.	G.N.E.	G.N.E.	G.N.E.	G.N.E.	G.N.E.	n.s.	*	G.N.E.	G.N.E.	n.s.	n.s.	G.N.E.
NNMT	n.s.	*	*	**	**	**	**	**	n.s.	**	**	**	**	**	**	n.s.	n.s.	n.s.	n.s.	n.s.	n.s.	***	***	****	**	***	***	n.s.
OCT1T	**	***	**	*	**	*	**	**	**	*	*	**	*	*	**	G.N.E.	G.N.E.	G.N.E.	G.N.E.	G.N.E.	G.N.E.	*	*	*	*	*	*	G.N.E.
RXRa	**	n.s.	**	**	**	*	*	n.s.	*	**	*	**	**	**	*	*	*	*	*	*	*	*	*	*	*	*	*	n.s.
SLCO2B1	G.N.E.	***	G.N.E.	n.s.	G.N.E.	***	**	G.N.E.	n.s.	*	**	G.N.E.	**	**	*	**	G.N.E.	**	**	**	**	**	G.N.E.	**	**	**	**	**
SULT1A1	*	*	n.s.	G.N.E.	G.N.E.	G.N.E.	****	n.s.	*	G.N.E.	**	*	**	G.N.E.	*	G.N.E.	G.N.E.	G.N.E.	G.N.E.	G.N.E.	G.N.E.	n.s.	n.s.	n.s.	G.N.E.	n.s.	n.s.	G.N.E.
SULT1A2	**	*	*	G.N.E.	G.N.E.	G.N.E.	**	n.s.	*	G.N.E.	**	n.s.	***	G.N.E.	n.s.	G.N.E.	G.N.E.	G.N.E.	G.N.E.	G.N.E.	G.N.E.	n.s.	n.s.	n.s.	G.N.E.	n.s.	n.s.	G.N.E.
TPMT	n.s.	*	*	*	*	n.s.	n.s.	n.s.	*	*	n.s.	n.s.	**	*	n.s.	G.N.E.	G.N.E.	G.N.E.	G.N.E.	G.N.E.	G.N.E.	*	*	*	*	*	*	G.N.E.
UGT1A1	**	**	**	*	**	**	**	n.s.	**	**	**	**	**	**	**	n.s.	n.s.	*	n.s.	n.s.	**	n.s.	n.s.	n.s.	n.s.	n.s.	n.s.	n.s.
UGT1A6	**	*	***	n.s.	**	**	**	n.s.	*	**	**	n.s.	**	**	n.s.	n.s.	n.s.	n.s.	n.s.	n.s.	n.s.	*	*	*	*	*	n.s.	n.s.
UGT1A9	G.N.E.	G.N.E.	*	G.N.E.	n.s.	*	G.N.E.	n.s.	**	*	G.N.E.	n.s.	**	n.s.	n.s.	G.N.E.	**	**	**	**	**	G.N.E.	*	*	*	*	*	n.s.
UGT2B4	*	*	n.s.	*	n.s.	n.s.	**	**	**	**	**	n.s.	**	n.s.	**	G.N.E.	G.N.E.	G.N.E.	G.N.E.	G.N.E.	G.N.E.	**	**	**	**	**	**	G.N.E.
UGT2B7	**	*	n.s.	G.N.E.	G.N.E.	G.N.E.	*	**	n.s.	G.N.E.	*	*	*	G.N.E.	n.s.	G.N.E.	G.N.E.	G.N.E.	G.N.E.	G.N.E.	G.N.E.	*	*	*	G.N.E.	*	*	G.N.E.

Key	
G.N.E	Gene not expressed in one or both conditions
n.s.	P > 0.05
*	P ≤ 0.05
**	P < 0.01
***	P < 0.001
****	P < 0.0001
