# Supplementary material for: Maturation of Induced Pluripotent Stem Cell Derived Hepatocytes by 3D-Culture
Source: PLoS One. 2014 Jan 22;9(1):e86372. doi: 10.1371/journal.pone.0086372 (PMC3899231; doi:10.1371/journal.pone.0086372)
Supplement: Table S7 — Significance of BOB7 RM qPCR analyses by Welch’s T-test. (PDF) [file pone.0086372.s017.pdf]

Gene	BOB7 RM D35 2D vs. BOB7 RM D25 2D	BOB7 RM D35 3DC vs. BOB7 RM D25 2D	BOB7 RM D35 3DC vs. BOB7 RM D35 2D	BOB7 RM D35 3DS vs. BOB7 RM D25 2D	BOB7 RM D35 3DS vs. BOB7 RM D35 2D	BOB7 RM D35 3DS vs. BOB7 RM D35 3DC	BOB7 RM D45 2D vs. BOB7 RM D25 2D	BOB7 RM D45 2D vs. BOB7 RM D35 2D	BOB7 RM D45 2D vs. BOB7 RM D35 3DC	BOB7 RM D45 2D vs. BOB7 RM D35 3DS	BOB7 RM D45 3DC vs. BOB7 RM D25 2D	BOB7 RM D45 3DC vs. BOB7 RM D35 2D	BOB7 RM D45 3DC vs. BOB7 RM D35 3DC	BOB7 RM D45 3DC vs. BOB7 RM D35 3DS
A1AT	n.s.	n.s.	**	n.s.	*	**	n.s.	n.s.	**	*	n.s.	n.s.	n.s.	****
ABCB4	n.s.	**	**	n.s.	n.s.	**	n.s.	*	**	*	**	*	n.s.	*
ABCC1	n.s.	**	**	*	*	**	n.s.	n.s.	**	*	n.s.	n.s.	**	***
ABCC2	**	*	*	*	**	*	*	**	*	**	n.s.	*	**	n.s.
ABCC4	*	**	*	*	*	**	n.s.	*	**	n.s.	*	*	**	*
AFP	*	n.s.	n.s.	*	*	**	n.s.	n.s.	**	**	n.s.	*	*	**
AHR	*	*	n.s.	**	n.s.	n.s.	*	*	*	***	***	n.s.	*	n.s.
ALB	**	**	**	**	**	***	***	*	***	***	*	**	*	*
CYP1A1	**	*	n.s.	**	**	*	**	**	*	*	*	**	*	****
CYP1A2	G.N.E.	G.N.E.	G.N.E.	G.N.E.	G.N.E.	G.N.E.	G.N.E.	G.N.E.	G.N.E.	G.N.E.	G.N.E.	G.N.E.	G.N.E.	G.N.E.
CYP1B1	**	*	*	**	**	*	*	**	*	n.s.	n.s.	n.s.	*	n.s.
CYP2C19	n.s.	**	**	n.s.	*	**	n.s.	n.s.	*	*	n.s.	*	n.s.	n.s.
CYP2C9	**	n.s.	**	*	**	*	*	n.s.	*	*	n.s.	**	*	**
CYP3A4	n.s.	n.s.	n.s.	n.s.	*	n.s.	*	*	*	*	*	*	*	n.s.
CYP3A5	n.s.	**	*	**	*	**	*	n.s.	**	**	**	*	*	**
CYP3A7	*	*	*	G.N.E.	G.N.E.	G.N.E.	**	**	**	G.N.E.	**	*	n.s.	G.N.E.
FXR	n.s.	*	n.s.	*	***	*	n.s.	**	n.s.	***	*	n.s.	*	**
GSTA4	n.s.	n.s.	n.s.	**	n.s.	*	n.s.	n.s.	n.s.	**	n.s.	n.s.	n.s.	***
GSTT1	**	n.s.	*	**	n.s.	*	n.s.	**	n.s.	**	n.s.	**	*	**
HNF4	n.s.	n.s.	*	*	**	*	n.s.	*	n.s.	***	*	**	**	n.s.
HNMT	*	n.s.	*	n.s.	*	n.s.	*	*	*	n.s.	n.s.	*	n.s.	n.s.
LXRa	n.s.	n.s.	*	*	**	n.s.	n.s.	n.s.	n.s.	*	*	***	*	n.s.
MAOA	**	n.s.	**	**	**	*	*	n.s.	*	**	*	**	***	**
MAOB	n.s.	**	**	***	**	**	n.s.	n.s.	n.s.	n.s.	n.s.	n.s.	n.s.	n.s.
MGST1	**	*	*	**	*	*	**	**	n.s.	*	*	***	n.s.	**
MGST2	**	n.s.	**	*	**	*	*	**	n.s.	n.s.	n.s.	**	n.s.	*
NAT1	n.s.	*	n.s.	n.s.	n.s.	*	n.s.	n.s.	*	n.s.	n.s.	n.s.	G.N.E.	n.s.
NNMT	*	*	**	*	*	n.s.	*	*	n.s.	n.s.	**	**	n.s.	**
OCT1T	**	*	**	***	**	*	**	**	*	**	**	**	****	***
RXRa	n.s.	*	**	*	***	*	n.s.	n.s.	*	*	*	**	n.s.	*
SLCO2B1	**	**	**	**	n.s.	**	*	*	n.s.	*	**	**	**	**
SULT1A1	**	*	**	**	n.s.	**	*	*	n.s.	*	*	**	n.s.	**
SULT1A2	n.s.	*	*	**	**	**	*	*	n.s.	**	*	*	*	**
TPMT	n.s.	n.s.	n.s.	**	**	*	n.s.	n.s.	n.s.	*	n.s.	n.s.	n.s.	**
UGT1A1	**	**	**	n.s.	**	**	***	*	**	**	**	**	**	**
UGT1A6	**	**	n.s.	*	**	**	**	*	*	**	**	**	n.s.	**
UGT1A9	**	**	**	**	**	**	n.s.	*	n.s.	n.s.	n.s.	**	n.s.	n.s.
UGT2B4	n.s.	**	**	**	**	**	n.s.	n.s.	**	**	n.s.	n.s.	n.s.	*
UGT2B7	n.s.	n.s.	n.s.	*	**	*	n.s.	**	n.s.	***	n.s.	n.s.	*	*

Gene	BOB7 RM D45 3DC vs. BOB7 RM D45 2D	BOB7 RM D45 3DS vs. BOB7 RM D25 2D	BOB7 RM D45 3DS vs. BOB7 RM D35 2D	BOB7 RM D45 3DS vs. BOB7 RM D35 3DC	BOB7 RM D45 3DS vs. BOB7 RM D35 3DS	BOB7 RM D45 3DS vs. BOB7 RM D45 2D	BOB7 RM D45 3DS vs. BOB7 RM D45 3DC	Primary Adult vs. BOB7 RM D25 2D	Primary Adult vs. BOB7 RM D35 2D	Primary Adult vs. BOB7 RM D35 3DC	Primary Adult vs. BOB7 RM D35 3DS	Primary Adult vs. BOB7 RM D45 2D	Primary Adult vs. BOB7 RM D45 3DC	Primary Adult vs. BOB7 RM D45 3DS
A1AT	n.s.	n.s.	*	**	n.s.	*	***	***	****	****	****	****	****	****
ABCB4	**	*	*	**	*	n.s.	**	*	*	*	*	*	*	*
ABCC1	n.s.	*	n.s.	**	**	*	**	n.s.	n.s.	**	*	n.s.	n.s.	n.s.
ABCC2	*	n.s.	****	*	*	*	*	*	*	n.s.	*	*	*	*
ABCC4	*	**	*	**	**	*	n.s.	**	*	**	**	**	n.s.	*
AFP	**	*	*	**	*	**	***	*	*	**	n.s.	**	***	***
AHR	***	****	**	n.s.	**	****	***	n.s.	n.s.	n.s.	n.s.	n.s.	n.s.	n.s.
ALB	**	**	**	***	*	***	*	n.s.	n.s.	n.s.	n.s.	n.s.	n.s.	n.s.
CYP1A1	n.s.	***	**	*	n.s.	*	**	n.s.	*	*	n.s.	n.s.	n.s.	n.s.
CYP1A2	G.N.E.	G.N.E.	G.N.E.	G.N.E.	G.N.E.	G.N.E.	G.N.E.	G.N.E.	G.N.E.	G.N.E.	G.N.E.	G.N.E.	G.N.E.	G.N.E.
CYP1B1	n.s.	n.s.	*	n.s.	n.s.	n.s.	n.s.	*	**	*	***	*	n.s.	n.s.
CYP2C19	*	G.N.E.	G.N.E.	G.N.E.	G.N.E.	G.N.E.	G.N.E.	n.s.	n.s.	n.s.	n.s.	n.s.	n.s.	G.N.E.
CYP2C9	*	n.s.	**	n.s.	*	*	n.s.	*	*	*	*	*	*	*
CYP3A4	**	n.s.	n.s.	n.s.	n.s.	**	n.s.	**	**	**	**	**	**	**
CYP3A5	**	**	*	*	**	**	**	n.s.	n.s.	n.s.	n.s.	n.s.	n.s.	n.s.
CYP3A7	**	G.N.E.	G.N.E.	G.N.E.	G.N.E.	G.N.E.	G.N.E.	n.s.	n.s.	n.s.	G.N.E.	n.s.	n.s.	G.N.E.
FXR	*	*	***	*	**	***	**	*	*	*	*	*	*	**
GSTA4	n.s.	n.s.	n.s.	n.s.	**	n.s.	n.s.	n.s.	n.s.	n.s.	*	n.s.	n.s.	n.s.
GSTT1	n.s.	n.s.	**	n.s.	**	n.s.	n.s.	n.s.	n.s.	n.s.	n.s.	n.s.	n.s.	n.s.
HNF4	***	*	**	*	*	***	*	n.s.	n.s.	n.s.	n.s.	n.s.	n.s.	n.s.
HNMT	n.s.	**	*	**	**	***	n.s.	*	*	**	**	**	n.s.	*
LXRa	n.s.	*	***	n.s.	n.s.	*	n.s.	*	*	*	*	*	*	*
MAOA	*	**	**	n.s.	**	**	**	*	*	*	*	*	*	*
MAOB	n.s.	G.N.E.	G.N.E.	G.N.E.	G.N.E.	G.N.E.	G.N.E.	*	*	*	*	*	*	G.N.E.
MGST1	*	*	***	*	**	**	**	*	*	*	*	*	*	*
MGST2	*	n.s.	**	n.s.	**	n.s.	n.s.	**	**	**	**	**	**	**
NAT1	n.s.	n.s.	n.s.	n.s.	n.s.	n.s.	n.s.	n.s.	n.s.	n.s.	n.s.	n.s.	n.s.	n.s.
NNMT	n.s.	**	**	*	*	*	n.s.	***	***	***	****	****	****	***
OCT1T	*	**	**	*	**	n.s.	n.s.	*	*	*	*	*	*	*
RXRa	*	*	**	***	**	n.s.	**	n.s.	n.s.	*	*	n.s.	*	*
SLCO2B1	*	**	*	**	*	*	n.s.	**	**	**	**	**	**	**
SULT1A1	n.s.	**	n.s.	**	n.s.	*	**	n.s.	n.s.	n.s.	n.s.	n.s.	n.s.	n.s.
SULT1A2	n.s.	**	**	**	**	*	**	n.s.	n.s.	n.s.	n.s.	n.s.	n.s.	n.s.
TPMT	n.s.	n.s.	n.s.	n.s.	**	n.s.	n.s.	*	*	*	*	*	*	*
UGT1A1	**	*	*	**	*	n.s.	**	n.s.	n.s.	n.s.	n.s.	n.s.	n.s.	n.s.
UGT1A6	n.s.	n.s.	**	**	*	**	**	*	*	*	*	*	*	*
UGT1A9	n.s.	n.s.	n.s.	n.s.	n.s.	n.s.	n.s.	*	*	*	*	*	*	n.s.
UGT2B4	n.s.	**	***	**	n.s.	**	*	**	**	**	**	**	**	**
UGT2B7	*	G.N.E.	G.N.E.	G.N.E.	G.N.E.	G.N.E.	G.N.E.	*	*	*	*	*	*	G.N.E.

Key	
G.N.E	Gene not expressed in one or both
n.s.	P > 0.05
*	P ≤ 0.05
**	P < 0.01
***	P < 0.001
****	P < 0.0001
